# Syntheses of fluorooxindole and 2-fluoro-2-arylacetic acid derivatives from diethyl 2-fluoromalonate ester

**DOI:** 10.3762/bjoc.10.119

**Published:** 2014-05-22

**Authors:** Antal Harsanyi, Graham Sandford, Dmitri S Yufit, Judith AK Howard

**Affiliations:** 1 Department of Chemistry, Durham University, South Road, Durham, DH1 3LE, UK; 2 Chemical Crystallography, Department of Chemistry, Durham University, South Road, Durham, DH1 3LE, UK

**Keywords:** fluorinated building blocks, fluoroarylacetic acid, fluoromalonate, fluorooxindole, organo-fluorine, selective fluorination

## Abstract

Diethyl 2-fluoromalonate ester is utilised as a building block for the synthesis of 2-fluoro-2-arylacetic acid and fluorooxindole derivatives by a strategy involving nucleophilic aromatic substitution reactions with *ortho*-fluoronitrobenzene substrates followed by decarboxylation, esterification and reductive cyclisation processes.

## Introduction

Since 1954, when Fried and Sabo observed that the incorporation of a fluorine atom into a corticosteroid derivative led to valuable enhanced biological activity [1], a growing number of commercially significant life science products, which owe their activity to the presence of fluorine atoms within their structures, have developed. Fluorine incorporation can lead, for example, to enhanced bioavailability, metabolic stability and lipophilicity of the organic system and these properties are exploited in a number of commercially valuable drugs including Ciprofloxacin, Lipitor and Voriconazole [2–6].

Given the very small number of fluorinated systems available from nature [7–9], in essence all organic molecules bearing carbon–fluorine bonds are ‘man-made’. Syntheses rely either on the construction of carbon–fluorine bonds using a fluorinating agent (‘late-stage’ fluorination) or the application of polyfunctional fluorine-containing small molecule building blocks (‘early stage’ fluorination) which may be employed in further transformations involving all the reactions and techniques available to synthetic organic chemists [10–13]. Of course, the success of an ‘early stage’ fluorination approach depends on the availability of a range of appropriately functionalised, fluorinated building blocks and the establishment of corresponding reactivity profiles [14]. However, it does not necessarily follow that reactions for which regio- and stereoselectivity profiles are well established for hydrocarbon systems will be similar to those for corresponding selectively fluorinated systems and, indeed, this is often not the case [15].

The use of 1,3-diketone, 1,3-ketoester and 1,3-diester derivatives in retrosynthetic planning is widespread in general organic chemistry and numerous terpenes, heterocycles and steroids originate from such simple yet synthetically versatile substrates [16–19]. In contrast, despite the availability of synthetic procedures for the preparation of various 2-fluoro-1,3-dicarbonyl systems [20–27], there is, surprisingly, only a relatively limited number of publications that report the use of such potentially useful fluorinated building blocks for the synthesis of more structurally sophisticated selectively fluorinated systems. For example, 2-fluoromalonate esters have been used for the preparation of various α-fluorocarboxylic acids [28–32], heterocycles, such as fluoropyrimidine [33] and quinolone [34] derivatives, alkylated [35] and Michael addition [36–40] products, providing an indication of the potential uses and opportunities available for the synthesis of fluoro-organic products from fluoromalonate precursors.

As part of a wider research programme aimed at developing routes for the synthesis of selectively fluorinated molecules using elemental fluorine for the key construction of the carbon–fluorine bond by complementary direct selective direct fluorination [41–44], continuous flow [45–49] and building block [50] strategies, in this paper, we describe nucleophilic aromatic substitution reactions of carbanions derived from diethyl 2-fluoromalonate ester as the first stage in the synthesis of fluoroacetic acid and fluoroxindole systems. While related palladium catalysed coupling processes between aryl bromides and diethyl 2-fluoromalonate have been described [51], reactions involving nucleophilic aromatic substitution between fluoromalonate systems [52] and appropriate aryl substrates have not been reported previously. Recently, various routes to fluorooxindoles have been discussed involving enantioselective fluorination of appropriate oxindole substrates by electrophilic fluorinating agents [53–62] or DAST [63] providing an indication of the importance of fluorooxindoles for medicinal chemistry applications.

## Results and Discussion

**Scheme 1:**
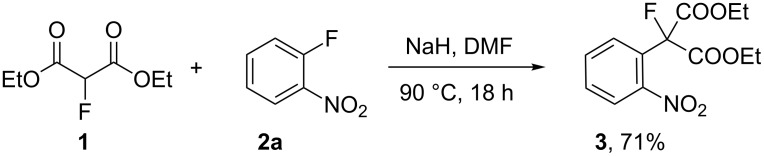
S_N_Ar reaction of 2-fluoronitrobenzene (**2a**) with diethyl 2-fluoromalonate (**1**).

**Figure 1:**
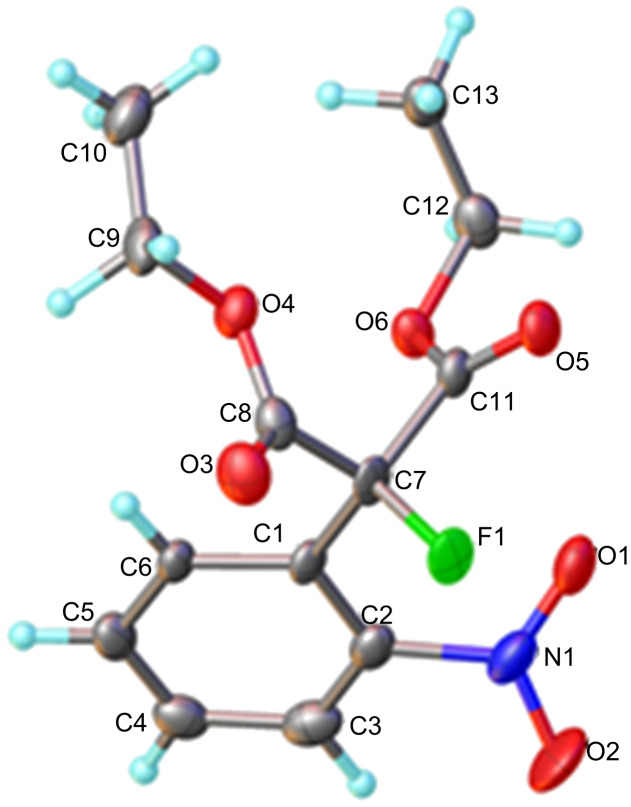
Molecular structure of **3**.

Reactions of carbanions generated by the addition of sodium hydride to a solution of diethyl 2-fluoromalonate (**1**) in DMF with *ortho*-fluoronitrobenzene (**2a**) led to the efficient displacement of fluorine by a nucleophilic aromatic substitution process to provide diester **3** in good yield (Scheme 1). Displacement of fluorine from *ortho*-fluoronitrobenzene was quantitative as measured by ^19^F NMR spectroscopy of the crude reaction mixture and the structure of isolated diester **3** was confirmed by X-ray crystallography (Figure 1).

In initial experiments, decarboxylation of **3** by reaction with potassium hydroxide gave good yields of the corresponding 2-fluoro-2-arylacetic acid **4a**. However, in subsequent experiments, we found that further purification of the diester **3** after the initial S_N_Ar step was not necessary and decarboxylation of crude diester **3** gave **4a** very efficiently. Consequently, in all analogous experiments (Table 1), crude product diesters of type **3** were isolated and used without further purification, allowing the ready synthesis of a range of arylfluoroacetic acid derivatives **4a–f** (Table 1). Structures **4a–f** were confirmed by NMR techniques and, in particular, a doublet located at −190 ppm (^2^*J*_HF_ = 50 Hz) in the ^19^F NMR spectra assigned to the CFH resonances and the corresponding doublets observed at ~6 ppm in the ^1^H NMR spectra, are diagnostic for the structures proposed.

**Table 1:** S_N_Ar reactions using fluoromalonate derivatives.

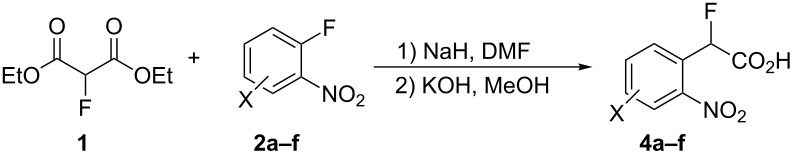		

Fluoronitroaryl 2	Fluoroarylacetic acid **4**	Yield

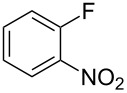 **2a**	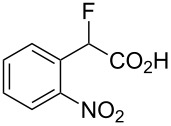 **4a**	62%
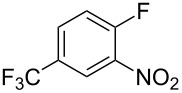 **2b**	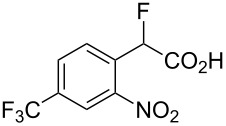 **4b**	77%
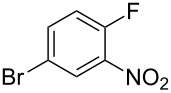 **2c**	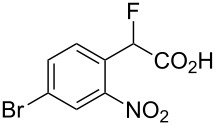 **4c**	83%
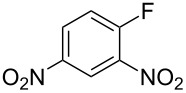 **2d**	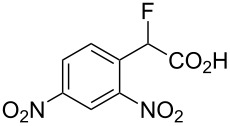 **4d**	56%
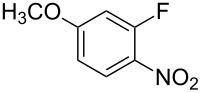 **2e**	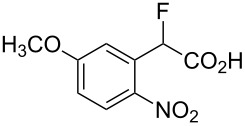 **4e**	60%
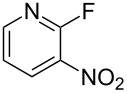 **2f**	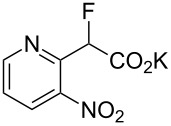 **4f**	86%

A nitro group *ortho* to a fluorine atom on the aryl ring is necessary under the present conditions to achieve full conversion of the starting fluoroarene. In related experiments, we found that a *para*-trifluoromethyl group is not sufficiently activating for reaction to occur whilst *para-*fluoronitrobenzene gave a complex mixture of unidentified products, most probably derived from competing benzyne formation.

This efficient methodology complements reported processes for the synthesis of various biologically active 2-fluoro-2-phenylacetic acids [64] which may be prepared using electrophilic fluorination of enolate esters [64–66], deoxofluorination [67–69] nucleophilic [70] and electrochemical fluorination [71–72] strategies.

**Scheme 2:**
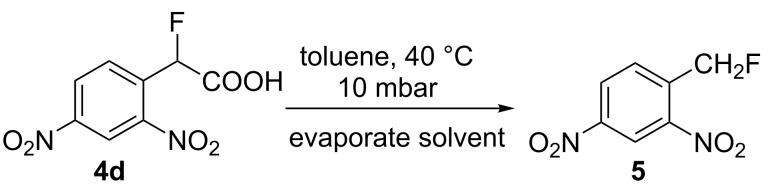
Synthesis of benzyl fluoride derivative **5**.

Attempts to prepare 2-fluoro-2-(2,4-dinitrophenyl)acetic acid by an analogous process led to the isolation of a benzyl fluoride derivative **5**, after evaporation of toluene and purification by column chromatography in 61% yield. The two consecutive decarboxylation reactions reflect the greater stability of the benzylic carbanion formed on loss of carbon dioxide from this system (Scheme 2).

**Figure 2:**
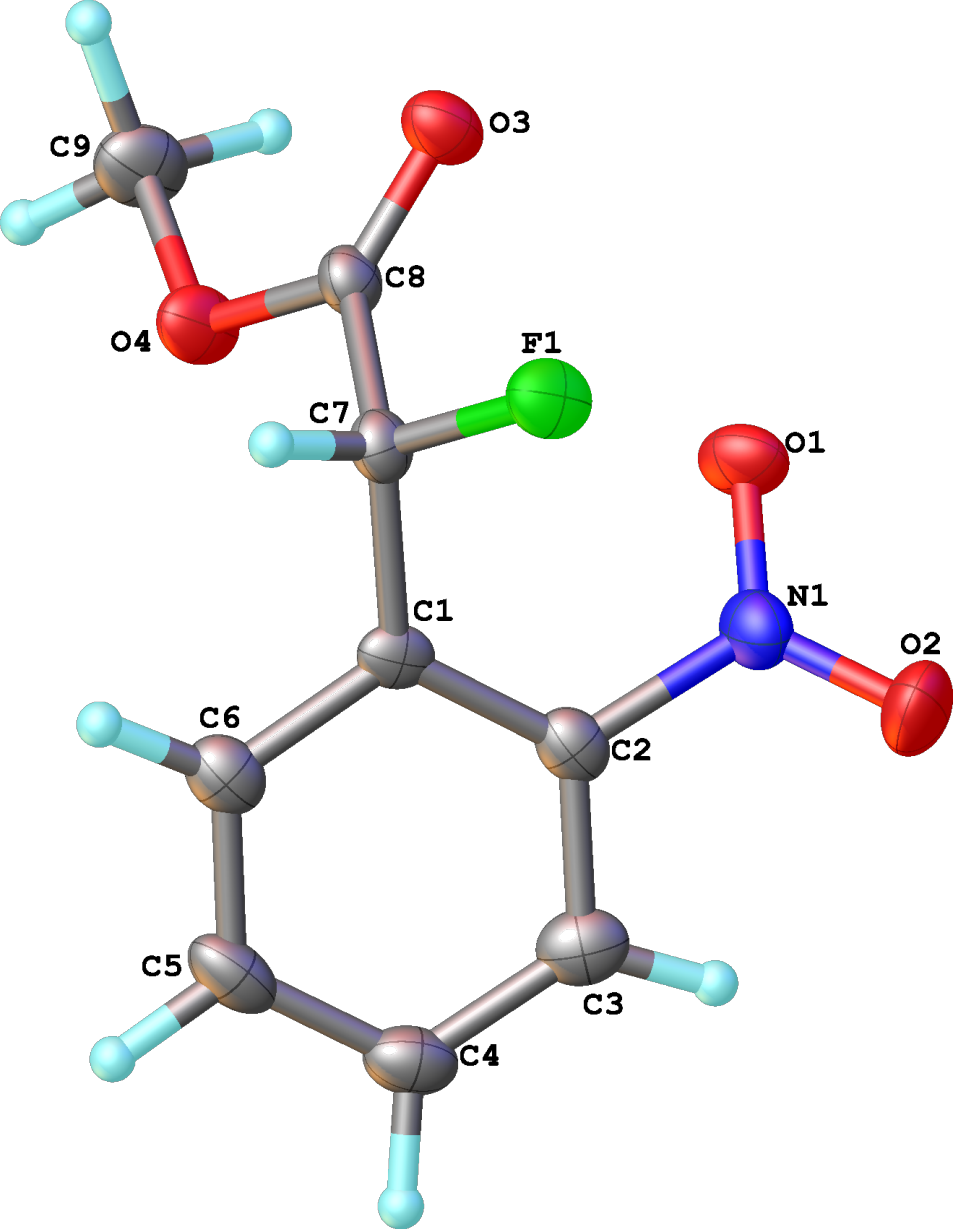
Molecular structure of methyl ester **6a**.

With the series of 2-fluorophenylacetic acids in hand, we attempted the reduction of the nitro group in **4a** using sodium dithionite, adapting reaction conditions similar to those described in the literature for the synthesis of biologically active system MaxiPost [63]. However, very low isolated yields of the cyclised product were obtained, presumably because of the high solubility of the amino acid intermediate in the aqueous reaction mixture and the well-established difficulty of direct amide bond formation processes. Consequently, before carrying out the nitro group reduction and amide forming cyclisation reactions, the acids **4a–e** were transformed to the corresponding methyl esters **6a–e** by stirring a mixture of the acid in hydrochloric acid and methanol (Table 2). The structure of **6a** was confirmed unambiguously by X-ray crystallography (Figure 2) and all other methyl esters **6b–e** were characterised by comparison with appropriate NMR data obtained for **6a**.

**Table 2:** Synthesis of methyl ester derivatives.

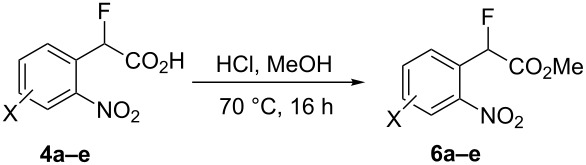		

Fluoroacetic acid 4	Methyl ester **6**	Yield

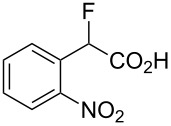 **4a**	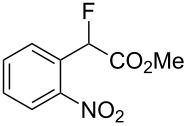 **6a**	88%
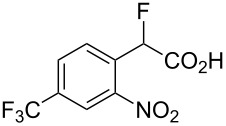 **4b**	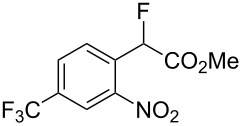 **6b**	98%
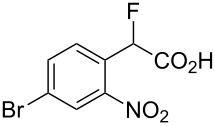 **4c**	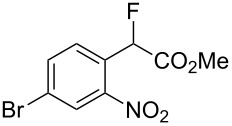 **6c**	97%
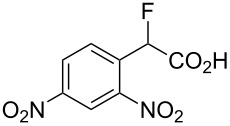 **4d**	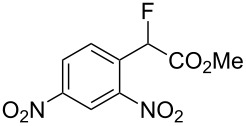 **6d**	65%
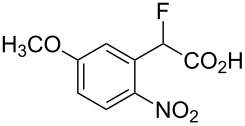 **4e**	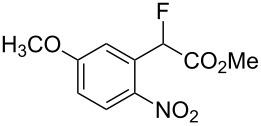 **6e**	98%

**Scheme 3:**
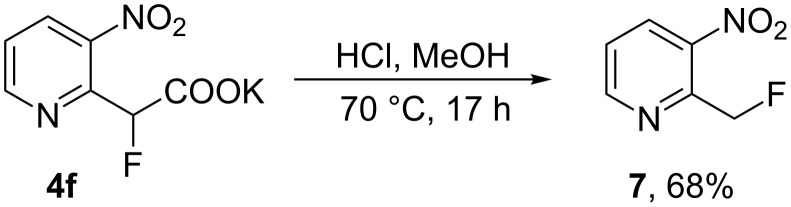
Synthesis of pyridyl fluoride **7**.

**Figure 3:**
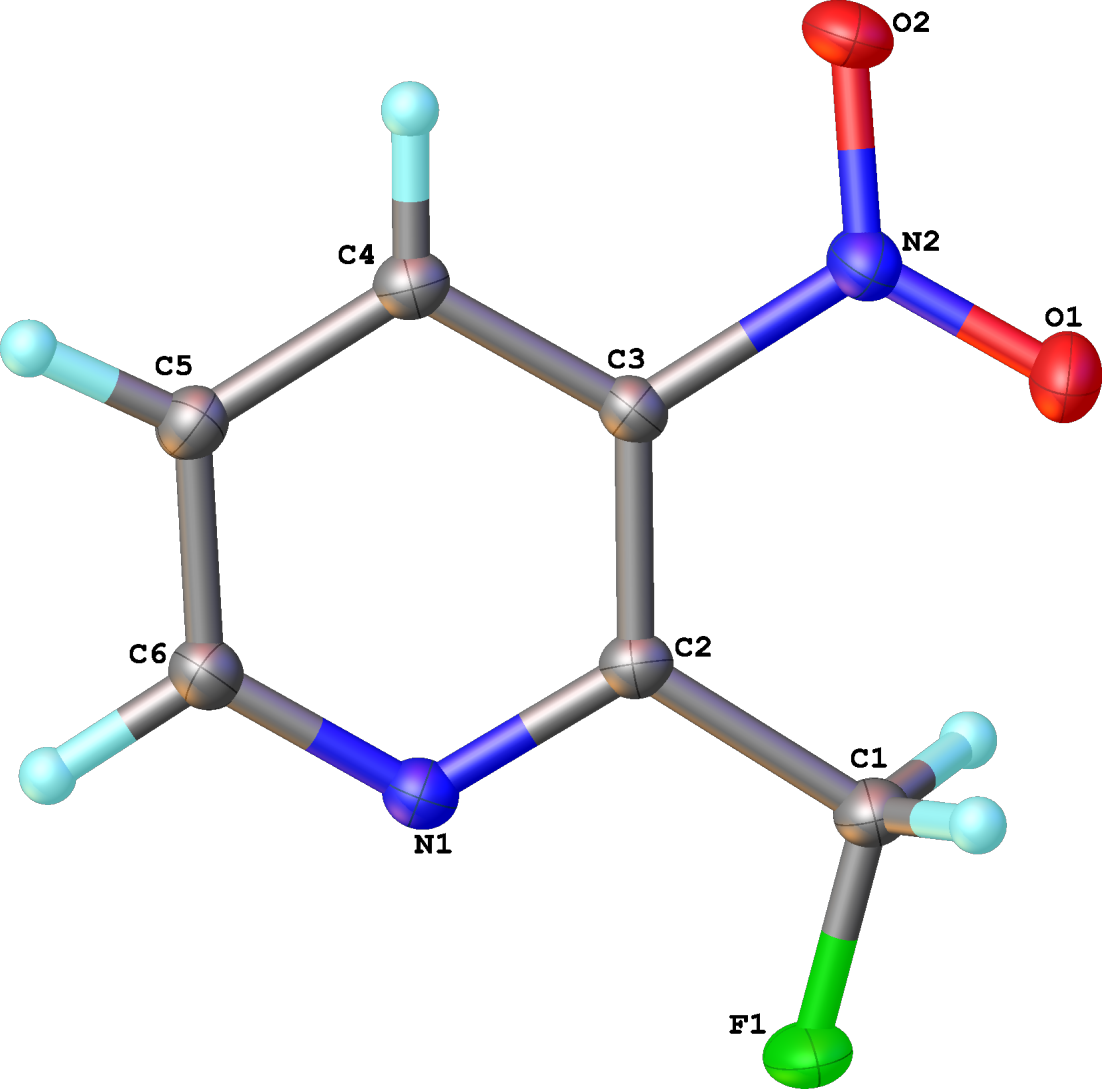
Molecular structure of **7**.

However, corresponding attempted esterification of the salt **4f** with HCl in methanol gave 2-fluoromethyl-3-nitropyridine (**7**) in 68% yield (Scheme 3) after purification of the crude material by column chromatography and the structure was confirmed by X-ray analysis (Figure 3). In this case competing decarboxylation, rather than esterification, reflects the greater stabilisation of the carbanion system formed upon decarboxylation for this system.

Reductive cyclization of methyl esters **6a–e** using sodium dithionite provided fluorooxindoles **8a–e** in acceptable yield after isolation by column chromatography (Table 3). In the ^1^H NMR spectrum, the characteristic CHF doublet located at 5.7 ppm (^2^*J*_HF_ = 51 Hz) for the fluorooxindole systems **8** are 0.9 ppm upfield from the corresponding CHF resonances of the arylfluoroacetic esters **6a–e** and, additionally, a broad NH singlet was detected at 9.0 ppm. The chemical shift of the doublet (−194.8 ppm) in the ^19^F NMR spectrum of fluorooxindoles **8a–e** is also observed 10 ppm upfield from the fluorine resonance of the starting esters **6a–e**.

**Table 3:** Synthesis of 3-fluorooxindoles.

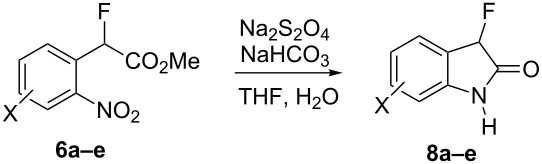		

Methyl ester **6**	Fluorooxindole **8**	Yield

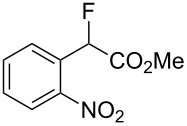 **6a**	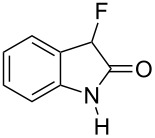 **8a**	32%
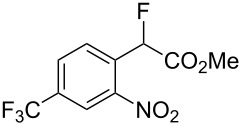 **6b**	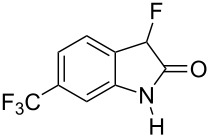 **8b**	82%
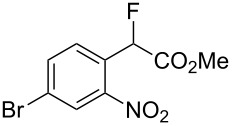 **6c**	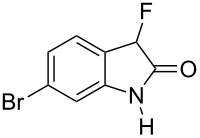 **8c**	57%
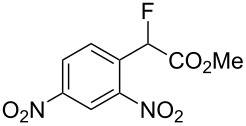 **6d**	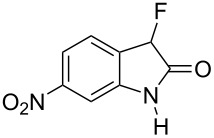 **8d**	0%
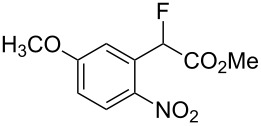 **6e**	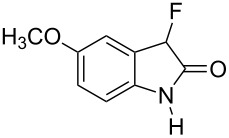 **8e**	30%

## Conclusion

Diethyl 2-fluoromalonate ester can be used as a highly effective fluorinated building block for the synthesis of various polyfunctional 2-fluoroacetic acid and 3-fluorooxindole systems. Fluorooxindoles are relatively rare fluorinated heterocyclic systems, even though several derivatives have useful biological activity, and current literature syntheses only involve fluorination of appropriate hydroxy and oxindole substrates. The strategy described here provides complementary building block syntheses from readily available fluorinated starting materials, further demonstrating the viability of using fluorinated dicarbonyl systems for the synthesis of more structurally sophisticated fluorinated derivatives.

## Supporting Information

File 1 Experimental procedures.

File 2 NMR spectra.

File 3 X-ray crystallographic data.
