# Supplementary material for: Syntheses of fluorooxindole and 2-fluoro-2-arylacetic acid derivatives from diethyl 2-fluoromalonate ester
Source: Beilstein J Org Chem. 2014 May 22;10:1213–9. doi: 10.3762/bjoc.10.119 (PMC4077372; doi:10.3762/bjoc.10.119)
Supplement: File 1 — Experimental procedures. [file Beilstein_J_Org_Chem-10-1213-s001.pdf]

Supporting Information File 1

for

Syntheses of fluorooxindole and 2-fluoro-2-arylacetic acid derivatives from diethyl 2-fluoromalonate ester

Antal Harsanyi[1], Graham Sandford[1*], Dmitri S. Yufit[2] and Judith A.K. Howard[2]

Address: [1]Department of Chemistry, Durham University, South Road, Durham, DH1 3LE, U.K, [2]Chemical Crystallography, Department of Chemistry, Durham University, South Road, Durham, DH1 3LE, U.K.

Email: Graham Sandford - graham.sandford@durham.ac.uk

*Corresponding author

Experimental procedures

General S3

Synthesis of diethyl 2-fluoro-2-(2-nitrophenyl)malonate '**3**' S3

Synthesis of 2-aryl-2-fluoroacetic acids **4a–f**: general procedure. S4

2-Fluoro-2-(2-nitrophenyl)acetic acid (**4a**) S5

2-Fluoro-2-(2-nitro-4-trifluoromethylphenyl)acetic acid (**4b**) S5

2-Fluoro-2-(2-nitro-4-bromophenyl)acetic acid (**4c**) S6

2-Fluoro-2-(2,4-dinitrophenyl)acetic acid (**4d**) S6

2-Fluoro-2-(2-nitro-5-methoxyphenyl)acetic acid (**4e**) S7

Potassium 2-fluoro-2-(3-nitro-2-pyridinyl)acetate (**4f**) S7

Synthesis of methyl esters **6a–e**: general procedure S8

Methyl 2-fluoro-2-(2-nitrophenyl)acetate (**6a**) S8

Methyl 2-fluoro-2-(2-nitro-4-trifluoromethylphenyl)acetate (**6b**) S9

Methyl 2-fluoro-2-(2-nitro-4-bromophenyl)acetate (**6c**) S9

Methyl 2-fluoro-2-(2,4-dinitrophenyl)acetate (**6d**) S10

Methyl 2-fluoro-2-(2-nitro-5-methoxyphenyl)acetate (**6e**) S10

Reductive cyclisation for the synthesis of fluorooxindole derivatives **8**:

general procedure S11

3-Fluorooxindole (**8a**) S11

3-Fluoro-6-trifluoromethyloxindole (**8b**) S11

3-Fluoro-6-bromooxindole (**8c**) S12

3-Fluoro-5-methoxyoxindole (**8e**) S12

Synthesis of fluoromethyl nitroarenes S13

2,4-Dinitrobenzyl fluoride (**5**) S13

2-Fluoromethyl-3-nitropyridine (**7**) S13

General

Analysis: Proton, carbon and fluorine nuclear magnetic resonance spectra (^{1}H NMR, ^{13}C NMR and ^{19}F NMR) were recorded (^{1}H NMR, 400 MHz; ^{13}C NMR, 100 MHz; ^{19}F NMR, 376 MHz) using solvent resonance as the internal standard (^{1}H NMR, $CHCl_3$ at 7.26 ppm; ^{13}C NMR, $CDCl_3$ at 77.36 ppm; ^{19}F NMR, $CFCl_3$ at 0.00 ppm). ^{1}H, ^{13}C and ^{19}F spectroscopic data are reported as follows: chemical shift, integration, multiplicity (s = singlet, d = doublet, t = triplet, m = multiplet), coupling constants (Hz), and assignment. Crystallographic data was recorded with a Rigaku R-Axis SPIDER IP diffractometer equipped with Cryostream (Oxford Cryosystems) low-temperature device at 120 K with graphite-monochromated Mo K_{α}-radiation (λ = 0.71073 Å). Melting points were measured at atmospheric pressure and are uncorrected.

Chemicals and solvents: Unless otherwise stated, commercially available reagents were used without purification. DMF was dried by colorimetric titration. Hexane, ethyl acetate, diethyl ether and DCM were purchased from Fischer and used without further purification. Flash column chromatography was carried out using Fluorochem Silicagel LC60A (40–63 micron).

Synthesis of diethyl 2-fluoro-2-(2-nitrophenyl)malonate (3)

NaH (0.88 g, 22 mmol 60% in mineral oil) was washed free from the oil with hexanes (3 × 10 mL) and was suspended in dry DMF (30 mL). Diethyl fluoromalonate (2.85 g, 16 mmol) dissolved in dry DMF (10 mL) was added dropwise while cooled in ice. After stirring for 10 minutes, 1-fluoro-2-nitrobenzene (2.10 g, 15 mmol) in dry DMF (10 mL) was added and the mixture was heated to 90 °C for 18 hours. After cooling to room temperature, the mixture

was poured into water (300 mL), acidified with concentrated HCl (2 mL) and extracted with ether (3 × 70 mL). The combined extracts were washed with saturated $NaHCO_3$ solution (2 × 50 mL) and brine (1 × 50 mL) and dried over Na_2SO_4. After evaporation of the solvent under reduced pressure an orange oil was obtained that was purified by Kugelrohr distillation (8 mbar, 200 °C) to afford diethyl 2-fluoro-2-(2-nitrophenyl)malonate (**3,** 3.21 g, 71%) as an orange solid. M.p. 48-49 °C; IR (neat, cm^{-1}) 2996, 1755, 1530, 1276, 1103; δ_H ($CDCl_3$, 400 MHz) 1.33 (6H, t, *J* 7.1, CH_3), 4.31 – 4.43 (4H, m, CH_2), 7.57 – 7.63 (2H, m, Ar-H), 7.66 – 7.71 (1H, m, Ar-H), 8.04 (1H, d, J 8.0, Ar-H); δ_F ($CDCl_3$, 376 MHz) - 152.98 (s, C-F); δ_C ($CDCl_3$, 100 MHz) 14.01 (s, CH_3), 63.55 (s, CH_2), 94.05 (d, $^1J_{CF}$ 198.7, C-F), 125.87 (s, Ar), 128.54 (s, Ar), 128.66 (s, Ar), 128.89 (s, Ar), 130.97 (s, Ar), 133.30 (s, Ar), 164.68 (d, $^2J_{CF}$ 25.1, COO); *m/z* (ASAP): 226.0 (85%, $[M-CO_2Et]^+$), 134.0 (100%).

Crystals suitable for X-ray crystallography were obtained by crystallisation from EtOH/H_2O (1:1 mixture).

Synthesis of 2-aryl-2-fluoroacetic acids* 4a–f*: general procedure.

Diethyl 2-fluoromalonate (**1**, 1.07 g, 6.0 mmol) in DMF (5 mL) was added dropwise to a suspension of sodium hydride (0.32 g, 8.0 mmol, 60% in mineral oil) in DMF (10 mL) and the mixture was stirred at room temperature for 20 min. Fluoroarene **2** (5.0 mmol) in DMF (10 mL) was added and the mixture was heated to 80 °C. The mixture was poured into crushed ice (150 mL), acidified with conc. HCl (5 mL) and extracted with diethyl ether (3 × 30 mL). The organic phase was washed with saturated aq. $NaHCO_3$ solution (25 mL) and saturated brine (2 × 25 mL), dried (Na_2SO_4) and concentrated to give the crude diester. ^1H and ^{19}F NMR analysis confirmed the formation of the intermediate aryl-fluoromalonate **3** (δ_F ~ –150 ppm) which was used in the next step without any further purification.

The aryl-fluoromalonate **3** was dissolved in anhydrous ethanol (40 mL) and KOH (0.68 g, 12.0 mmol) in ethanol (10 mL) was added dropwise with cooling (0 °C). The mixture was stirred for 1 h, hexane (50 mL) was added, and stirring continued for a further 30 min. The solid formed was filtered and washed with hexane:ethanol (20 mL, 1:1 mixture). The resulting solid was dissolved in water (40 mL), the solution acidified with conc. HCl (5 mL) and extracted with ethyl acetate (3 × 30 mL). The organic phase was washed with saturated brine (2 × 20 mL) and dried (Na_2SO_4). After filtration, the solvent was evaporated under reduced pressure to give pure 2-aryl-2-fluoroacetic acid derivative **4** which was purified by recrystallisation if required.

2-Fluoro-2-(2-nitrophenyl)acetic acid (4a)

2-Fluoronitrobenzene (**2a**, 0.71 g, 5.0 mmol) gave 2-fluoro-2-(2-nitrophenyl)acetic acid (**4a**, 0.62 g, 62%) as a yellow powder; mp 119-121 °C (Found: C, 48.2, H, 3.0, N, 6.9. $C_8H_6FNO_4$ requires: C, 48.25, H, 3.0, N, 7.0%); IR (neat, cm^{-1}) 3014 (br), 1732, 1530, 1340, 1204, 1063; δ_H (d_6-DMSO , 400 MHz) 6.52 (1H, d, $^2J_{HF}$ 45.5, CFH), 7.70 - 7.75 (2H, m, ArH), 7.86 (1H, t, $^3J_{HH}$ 7.6, ArH), 8.14 (1H, d, $^3J_{HH}$ 7.6, ArH), 13.40 (1H, bs, COOH); δ_F (d_6-DMSO, 376 MHz) - 185.10 (d, $^2J_{HF}$ 45.5); δ_C (d_6-DMSO, 100 MHz) 86.62 (d, $^1J_{CF}$ 181.7, CHF), 124.96 (s, C-3), 128.85 (d, $^3J_{FC}$ 11.0, C6), 129.79 (d, $^2J_{CF}$ 20.3, C-1), 132.52 (d, $^4J_{CF}$ 1.6, C-5), 134.20 (d, $^5J_{CF}$ 1.1, C-4), 146.91 (s, C-2), 169.94 (d, $^2J_{CF}$ 24.2, COOH); *m/z* (ESI) 154 (100%, $[M-COOH]^+$), 104 (23). [1]

2-Fluoro-2-(2-nitro-4-trifluoromethylphenyl)acetic acid (4b)

3-Nitro-4-fluorobenzotrifluoride (**2b**, 1.05 g, 5.0 mmol) gave 2-fluoro-2-(2-nitro-4-trifluoromethylphenyl)acetic acid (**4b**, 1.03 g, 77%) as a white powder; mp 110-112 °C (Found: C, 40.2; H, 1.9; N, 5.1. $C_9H_5F_4NO_4$ requires: C, 40.5; H, 1.9; N, 5.2%); IR (neat, cm^{-1}) 3018 (br), 1736, 1549, 1319, 1196, 1131, 1096, 1059; δ_H (CD_3OD, 400 MHz) 6.63 (1H, d,

$^2J_{HF}$ 46.3, CFH), 7.96 (1H, d, $^3J_{HH}$ 8.4, ArH), 8.09 (1H, d, $^3J_{HH}$ 8.4, ArH), 8.41 (1H, s, ArH); δ_F (CD_3OD, 376 MHz) - 67.11 (3F, s, CF_3), - 192.48 (1F, d, $^2J_{HF}$ 46.3, CHF); δ_C (CD_3OD, 100 MHz) 87.88 (d, $^1J_{CF}$ 186.3, CHF), 123.32 (q, $^3J_{CF}$ 4.0, C-3), 124.27 (q, $^1J_{CF}$ 271.5, CF_3), 130.24 (d, $^3J_{CF}$ 14.4, C-6), 131.52 – 131.54 (m, C-5), 133.19 (q, $^2J_{CF}$ 33.6, C-4), 136.00 (d, $^3J_{CF}$ 21.3, C-1), 148.77 (s, C-2), 169.06 (d, $^2J_{CF}$ 24.3, COOH); *m/z* (ASAP) 222 (100%).

2-Fluoro-2-(2-nitro-4-bromophenyl)acetic acid (4c)

2-Fluoro-5-bromonitrobenzene (**2c**, 1.10 g, 5.0 mmol) gave 2-fluoro-2-(2-nitro-4-bromophenyl)acetic acid (**4c**, 1.15 g, 83%) as a tan powder; mp 155-158 °C (Found: C, 34.5, H, 1.8, N, 4.9. $C_8H_5BrFNO_4$ requires: C, 34.6, H, 1.8, N, 5.0%); IR (neat, cm^{-1}) 3084 (br), 2360, 1693, 1537, 1339, 1205, 1060; δ_H (CD_3OD, 400 MHz) 6.51 (1H, d, $^2J_{HF}$ 46.4, CFH), 7.65 (1H, d, $^3J_{HH}$ 8.4, ArH), 7.94 (1H, dd, $^3J_{HH}$ 8.4, $^4J_{HF}$ 2.0, ArH), 8.27 - 8.29 (1H, m, ArH); δ_F (CD_3OD, 376 MHz) - 189.70 (d, $^2J_{HF}$ 46.4); δ_C (CD_3OD, 100 MHz) 87.66 (d, $^1J_{CF}$ 182.9, CHF), 124.19 (d, $^4J_{CF}$ 1.9, C-5), 129.02 (s, C-3), 130.80 (d, $^3J_{CF}$ 13.3, C-6), 130.98 (d, $^2J_{CF}$ 21.4, C-1), 137.99 (d, $^5J_{CF}$ 1.5, C-4), 149.10 (s, C-2), 169.47 (d, $^2J_{CF}$ 24.3, COOH); *m/z* (ASAP) 234 (96%), 232 (100 %).

2-Fluoro-2-(2,4-dinitrophenyl)acetic acid (4d)

1-Fluoro-2,4-dinitrobenzene (**2d**, 1.50 g, 8 mmol) was used to synthesise the crude fluoromalonate as above which was dissolved in glacial acetic acid (25 mL) and water (15 mL). Conc. sulfuric acid (4 mL) was added and the mixture was heated at 100 °C for 25 h. The mixture was poured into crushed ice (350 mL), extracted with DCM (3 × 100 mL) and the organic phase was evaporated. The residue was dissolved in saturated $NaHCO_3$ solution (60 mL) and extracted with DCM (2 × 30 mL). The aqueous solution was acidified to pH 1 with concentrated aq. HCl and extracted with DCM (3 × 50 ml). The organic phase was dried (Na_2SO_4), filtered and evaporated to give 2-fluoro-2-(2,4-dinitrophenyl)acetic acid (**4d**, 1.10

g, 56%) as an orange solid; mp 74-77 °C; IR (neat, cm^{-1}) 3585, 3109, 2481, 1715, 1521, 1354, 1093; δ_H ($CDCl_3$, 400 MHz) 6.80 (1H, d, $^2J_{HF}$ 46.5, CFH), 8.05 (1H, d, $^3J_{HH}$ 8.5, ArH), 8.59 (1H, dd, $^3J_{HH}$ 8.5, $^4J_{HF}$ 2.4, ArH), 8.72 (1H, bs, COOH), 9.00 – 9.01 (1H, m, ArH); δ_F ($CDCl_3$, 376 MHz) - 190.37 (d, $^2J_{HF}$ 46.5); δ_C ($CDCl_3$, 100 MHz) 86.16 (d, $^1J_{CF}$ 189.4, CHF), 120.88 (s, C-3), 128.38 (d, $^4J_{CF}$ 2.3, C-5), 129.29 (d, $^3J_{CF}$ 15.9, C-6), 136.06 (d, $^2J_{CF}$ 21.5, C-1), 147.14 (s, C-4), 148.51 (s, C-2), 169.21 (d, $^2J_{CF}$ 23.6, COOH); *m/z* (ASAP) 199 (55 %), 179 (44).

2-Fluoro-2-(2-nitro-5-methoxyphenyl)acetic acid (4e)

3-Fluoro-4-nitroanisole (**2e**, 1.0 g, 5.8 mmol) gave 2-fluoro-2-(2-nitro-5-methoxyphenyl)-acetic acid (**4e**, 0.80 g, 60%) as a tan powder; mp 129–131 °C (Found: C, 46.9; H, 3.5; N, 6.1. $C_9H_8FNO_5$ requires: C, 47.2; H, 3.5; N, 6.1%); IR (neat, cm^{-1}) 2848 (br), 1724, 1582, 1324, 1283, 1235, 1087; δ_H (d_6-DMSO, 400 MHz) 3.92 (3H, s, CH_3), 6.52 (1H, d, $^2J_{HF}$ 46.0, CFH), 7.18 - 7.24 (2H, m, ArH), 8.22 (1H, d, $^3J_{HH}$ 8.9, ArH), 13.73 (1H, s, COOH); δ_F (d_6-DMSO, 376 MHz) - 185.36 (d, $^2J_{HF}$ 46.0); δ_C (d_6-DMSO, 100 MHz) 56.37 (s, CH_3), 87.39 (d, $^1J_{CF}$ 183.2, CHF), 114.18 (d, $^3J_{CF}$ 13.5, C-6), 114.45 (s, C-4), 128.10 (s, C-3), 133.21 (d, $^2J_{CF}$ 19.8, C-1), 139.45 (d, $^3J_{CF}$ 1.9, C-2), 163.58 (d, $^4J_{CF}$ 1.4, C-5), 167.73 (d, $^2J_{CF}$ 23.5, COOH); *m/z* (ASAP) 184 (100%,), 164 (24).

Potassium 2-fluoro-2-(3-nitro-2-pyridinyl)acetate (4f)

2-Fluoro-3-nitropyridine (**2f**, 0.72 g, 5.0 mmol) gave potassium 2-fluoro-2-(3-nitro-2-pyridinyl)acetate (**4f**, 1.03 g, 86%) as a deep red powder; mp >140 °C (decomposes); IR (neat, cm^{-1}) 1654, 1523, 1359; δ_H (D_2O, 400 MHz) 6.32 (1H, d, $^2J_{HF}$ 47.7, CFH), 7.69 – 7.73 (1H, m, ArH), 8.52 (1H, d, $^3J_{HH}$ 8.4, ArH), 8.80 (1H, dd, $^3J_{HH}$ 4.9, $^4J_{HF}$ 1.4, ArH); δ_F (D_2O, 376 MHz) - 180.27 (d, $^2J_{HF}$ 47.7); δ_C (D_2O, 100 MHz) 87.91 (d, $^1J_{CF}$ 185.2, CHF), 124.29 (d,

$^{5}J_{CF}$ 1.6, C-5), 133.25 (s, C-4), 143.85 (s, C-6), 148.27 (d, $^{2}J_{CF}$ 20.0, C-2), 151.82 (s, C-3), 171.86 (d, $^{2}J_{CF}$ 21.6, COOH); *m/z* (ASAP) 155 (8%), 137 (100).

Synthesis of methyl esters 6a–e: general procedure

2-Fluoro-2-arylacetic acid ‘**4**’ (20–30 mmol) was dissolved in methanol (50 mL) and HCl in methanol (1.4 M, 10 mL, 14 mmol) was added. The mixture heated to reflux for 16 h then the solvent was evaporated. The resulting oil was partitioned between DCM (50 mL) and saturated aqueous $NaHCO_3$ solution (20 mL). The aqueous phase was extracted with DCM (2 × 20 mL), the combined organic phase was washed with saturated brine (20 mL) and dried (Na_2SO_4). Filtration and evaporation gave the methyl 2-fluoro-2-arylacetate derivative **6** which was purified by distillation or column chromatography if required.

Methyl 2-fluoro-2-(2-nitrophenyl)acetate (6a)

2-Fluoro-2-(2-nitrophenyl)acetic acid (**4a**, 5.98 g, 30 mmol) after vacuum distillation gave methyl 2-fluoro-2-(2-nitrophenyl)acetate (**6a**, 5.67 g, 88%) as a yellow crystalline solid; mp 41-43 °C; bp 110-112 °C (5 mbar) ($[MH]^+$: 214.0500. $C_9H_8FNO_4$ requires: $[MH]^+$, 214.0516); IR (neat, cm^{-1}) 1749, 1526, 1348, 1216, 1022; δ_H ($CDCl_3$, 400 MHz) 3.76 (3H, s, CH_3), 6.57 (1H, d, $^{2}J_{HF}$ 46.7, CFH), 7.55 - 7.59 (1H, m, ArH), 7.70 - 7.75 (2H, m, ArH), 8.12 (1H, d, $^{3}J_{HH}$ 8.4, ArH); δ_F ($CDCl_3$, 376 MHz) - 188.04 (d, $^{2}J_{HF}$ 46.7); δ_C ($CDCl_3$, 100 MHz) 53.07 (s, CH_3), 86.60 (d, $^{1}J_{CF}$ 185.4, CHF), 125.07 (s, C-4), 127.80 (d, $^{3}J_{CF}$ 15.1, C-6), 130.12 (d, $^{4}J_{CF}$ 1.3, C-5), 130.17 (d, $^{2}J_{CF}$ 21.1, C-1), 134.21 (d, $^{4}J_{CF}$ 1.9, C-3), 146.82 (d, $^{3}J_{CF}$ 3.2, C-2), 167.13 (d, $^{2}J_{CF}$ 25.0, COO); *m/z* (ASAP) 214 (4 %, $[MH]^+$), 194 (8), 154 (100).

Methyl 2-fluoro-2-(2-nitro-4-trifluoromethylphenyl)acetate (6b)

2-Fluoro-2-(2-nitro-4-trifluoromethylphenyl)acetic acid (**4b**, 5.91 g, 22 mmol) gave methyl 2-fluoro-2-(2-nitro-4-trifluoromethylphenyl)acetate (**6b**, 6.10 g, 98%) as a brown oil; ($[MH]^+$, 282.0394. $C_{10}H_7F_4NO_4$ requires: $[MH]^+$, 282.0389); IR (neat, cm^{-1}) 1752, 1543, 1324, 1179, 1133, 1095; δ_H ($CDCl_3$, 400 MHz) 3.81 (3H, s, CH_3), 6.70 (1H, d, $^2J_{HF}$ 46.4, CFH), 7.96 - 8.05 (2H, m, ArH), 8.44 (1H, s, ArH); δ_F ($CDCl_3$, 376 MHz) - 64.09 (3F, s, CF_3), - 189.74 (1F, d, $^2J_{HF}$ 46.4, CHF); δ_C ($CDCl_3$, 100 MHz) 53.55 (s, CH_3), 86.54 (d, $^1J_{CF}$ 186.2, CHF), 122.65 (q, $^1J_{CF}$ 272.6, CF_3), 122.66 (q, $^3J_{CF}$ 3.7, C-3), 128.70 (d, $^3J_{CF}$ 16.0, C-6), 130.77 - 130.85 (m, C-5), 132.80 (q, $^2J_{CF}$ 34.8, C-4), 134.25 (d, $^2J_{CF}$ 21.8, C-1), 146.97 (s, C-2), 166.41 (d, $^2J_{CF}$ 24.0, COO); *m/z* (ASAP) 282 (13%, $[MH]^+$), 222 (100).

Methyl 2-fluoro-2-(2-nitro-4-bromophenyl)acetate (6c)

2-Fluoro-2-(2-nitro-4-bromophenyl)acetic acid (**4c**, 5.83 g, 21 mmol) gave methyl 2-fluoro-2-(2-nitro-4-bromophenyl)acetate (**6c**, 5.93 g, 97%) as a tan powder; mp 78-79 °C; ($[MH]^+$, 291.9641. $C_9H_7[^{79}Br]FNO_4$ requires: $[MH]^+$, 291.9621); IR (neat, cm^{-1}) 1744, 1525, 1345, 1214, 1021, 986; δ_H ($CDCl_3$, 400 MHz) 3.79 (3H, s, CH_3), 6.56 (1H, d, $^2J_{HF}$ 46.5, CFH), 7.65 (1H, d, $^3J_{HH}$ 8.6, ArH), 7.86 (1H, dd, $^3J_{HH}$ 8.5, $^4J_{HH}$ 2.0, ArH), 8.29 (1H, s, ArH). δ_F ($CDCl_3$, 376 MHz) -188.89 (d, $^2J_{HF}$ 46.5); δ_C ($CDCl_3$, 100 MHz) 53.38 (s, CH_3), 86.39 (d, $^1J_{CF}$ 185.4, CHF), 123.70 (d, $^5J_{CF}$ 2.0, C-4), 128.31 (s, C-3), 129.23 (d, $^3J_{CF}$ 14.8, C-6), 129.39 (d, $^2J_{CF}$ 21.7, C-1), 137.27 (d, $^4J_{CF}$ 1.7, C-5), 147.28 (s, C-2), 166.73 (d, $^2J_{CF}$ 24.7, COO); *m/z* (ASAP) 292 (6%, $[M(^{79}Br)H]^+$), 294 (6%, $[M(^{81}Br)H]^+$), 272 (15), 274 (15), 232 (100), 234 (96).

Methyl 2-fluoro-2-(2,4-dinitrophenyl)acetate (6d)

2-Fluoro-2-(2,4-dinitrophenyl)acetic acid (**4d**, 4.69 g, 19 mmol) after purification by column chromatography on silica gel using hexane:ethyl acetate (3:1) as elutant, R_f: 0.36) gave methyl 2-fluoro-2-(2,4-dinitrophenyl)acetate (**6d**, 3.21 g, 65%) as a yellow oil; ($[MH]^+$, 259.0372. $C_9H_7FN_2O_6$ requires: $[MH]^+$, 259.0366); IR (neat, cm^{-1}) 3101, 1760, 1530, 1349, 1228, 1087; δ_H ($CDCl_3$, 400 MHz) 3.82 (3H, s, CH_3), 6.74 (1H, d, $^2J_{HF}$ 46.8, CFH), 8.06 (1H, d, $^3J_{HH}$ 8.6, ArH), 8.59 (1H, dd, $^3J_{HH}$ 8.6, $^4J_{HH}$ 2.3, ArH), 9.00 - 9.02 (1H, m, ArH); δ_F ($CDCl_3$, 376 MHz) - 189.83 (d, $^2J_{HF}$ 46.8); δ_C ($CDCl_3$, 100 MHz) 53.73 (s, CH_3), 86.56 (d, $^1J_{CF}$ 187.8, CHF), 120.77 (s, C-3), 128.31 (d, $^4J_{CF}$ 2.3, C-5), 129.17 (d, $^3J_{CF}$ 16.7, C-6), 136.66 (d, $^2J_{CF}$ 21.4, C-1), 147.17 (s, C-4), 148.39 (s, C-2), 165.95 (d, $^2J_{CF}$ 23.7, COO); *m/z* (ASAP) 259 (10 %, $[MH]^+$), 212 (54), 199 (100).

Methyl 2-fluoro-2-(2-nitro-5-methoxyphenyl)acetate (6e)

2-Fluoro-2-(2-nitro-5-methoxyphenyl)acetic acid (**4e**) gave methyl 2-fluoro-2-(2-nitro-5-methoxyphenyl)acetate (**6e**, 4.35 g, 98%) as a brown oil; ($[MH]^+$, 244.0598. $C_{10}H_{10}FNO_5$ requires: $[MH]^+$, 244.0621); IR (neat, cm^{-1}) 2956, 1751, 1582, 1514, 1340, 1288, 1238; δ_H ($CDCl_3$, 400 MHz) 3.76 (3H, s, CH_3), 3.90 (3H, s, CH_3), 6.57 (1H, d, $^2J_{HF}$ 46.9, CFH), 6.97 (1H, dd, $^3J_{HH}$ 9.3, $^4J_{HH}$ 2.8, ArH), 7.18 (1H, d, $^4J_{HH}$ 3.0, ArH), 8.17 (1H, dd, $^3J_{HH}$ 9.3, $^5J_{HH}$ 1.0, ArH); δ_F ($CDCl_3$, 376 MHz) - 187.68 (d, $^2J_{HF}$ 46.9); δ_C ($CDCl_3$, 100 MHz) 53.06 (s, CH_3), 56.16 (s, CH_3), 87.18 (d, $^1J_{CF}$ 183.5, CHF), 112.66 (d, $^3J_{CF}$ 16.8, C-6), 114.41 (s, C-4), 128.03 (s, C-3), 133.42 (d, $^2J_{CF}$ 20.6, C-1), 139.53 (d, $^3J_{CF}$ 3.0, C-2), 164.06 (d, $^4J_{CF}$ 2.4, C-5) 166.92 (d, $^2J_{CF}$ 24.5, COO); *m/z* (ASAP) 244 (7 %, $[MH]^+$), 212 (24), 198 (17), 184 (100%).

Reductive cyclisation for the synthesis of fluorooxindole derivatives 8: general procedure

The methyl 2-fluoro-2-(2-nitroaryl)acetate derivative **6** (5 mmol) was dissolved in THF (20 mL) and water (20 mL). $NaHCO_3$ (1.68 g, 20 mmol) was added and the mixture was stirred vigorously. $Na_2S_2O_4$ (2.61 g, 15 mmol) was added over 40 min and the mixture was stirred for 20 min. Ethyl acetate (40 mL) was added, the aqueous layer was removed, HCl in methanol (3 mL, 1.4 M, 4.2 mmol) was added and the mixture was heated to reflux for 3 h. The solution was cooled, dried (Na_2SO_4), filtered and evaporated. The residue was purified by column chromatography on silica gel using hexane:ethyl acetate (5:1) as eluent to give fluorooxindole derivative **8**.

3-Fluorooxindole (8a)

Methyl 2-fluoro-2-(2-nitrophenyl)acetate (**6a**, 1.06 g, 5 mmol) gave 3-fluorooxindole (**8a**, 0.24 g, 32%) as a yellow powder; R_f: 0.21; mp 92-94 °C; ($[MH]^+$, 152.0496. C_8H_6FNO requires: $[MH]^+$, 152.0512); IR (neat, cm^{-1}) 3188, 1772, 1642, 1050; δ_H ($CDCl_3$, 400 MHz) 5.70 (1H, d, $^2J_{HF}$ 51.0, CFH), 6.91 (1H, dm, $^3J_{HH}$ 7.8, ArH), 7.11 (1H, tt, $^3J_{HH}$ 7.6, $^5J_{HH}$ 0.8, ArH), 7.35 (1H, tm, $^3J_{HH}$ 7.8, ArH), 7.46 (1H, dm, $^3J_{HH}$ 7.4, ArH), 8.42 (1H, bs, N-H); δ_F ($CDCl_3$, 376 MHz) - 194.56 (d, $^2J_{HF}$ 51.0); δ_C ($CDCl_3$, 100 MHz) 85.87 (d, $^1J_{CF}$ 189.4, CHF), 110.84 (d, $^5J_{CF}$ 1.5, C-7), 123.36 (d, $^2J_{CF}$ 16.4, C-4), 123.52 (d, $^4J_{CF}$ 3.0, C-8), 126.57 (d, $^4J_{CF}$ 1.7, C-6), 131.68 (d, $^3J_{CF}$ 3.3, C-5), 141.92 (d, $^3J_{CF}$ 5.5, C-9), 173.39 (d, $^2J_{CF}$ 17.9, C=O); *m/z* (ASAP) 152 (100 %, $[MH]^+$), 132 (53).

3-Fluoro-6-trifluoromethyloxindole (8b)

Methyl 2-fluoro-2-(2-nitro-4-trifluoromethylphenyl)acetate (**6b**, 1.41 g, 5 mmol) gave 3-fluoro-6-trifluoromethyloxindole (**8b**, 0.90 g, 82%) as a pale yellow powder; R_f: 0.17; mp

175-177 °C; ([M]$^+$, 219.0305. $C_9H_5F_4NO$ requires: [M]$^+$, 219.0307); IR (neat, cm^{-1}) 3143, 1753,1698, 1317, 1292, 1257, 1114, 1054; δ_H (d_6-DMSO, 400 MHz) 5.99 (1H, d, $^2J_{HF}$ 49.5, CFH), 7.08 (1H, s, ArH), 7.41 (1H, d, $^3J_{HH}$ 7.6, ArH), 7.68 (1H, d, $^3J_{HH}$ 7.6, ArH), 10.91 (1H, bs, N-H); δ_F (d_6-DMSO, 376 MHz) - 62.55 (3F, s, CF_3), - 195.89 (1F, d, $^2J_{HF}$ 49.5, CHF); δ_C (d_6-DMSO, 100 MHz) 85.44 (d, $^1J_{CF}$ 184.9, CHF), 106.50 (m, C-8), 119.11 (m, C-6), 123.70 (q, $^1J_{CF}$ 271.5, CF_3), 126.99 (s, C-5), 127.67 (d, $^2J_{CF}$ 15.6, C-4), 131.48 (qd, $^2J_{CF}$ 32.1, $^4J_{CF}$ 3.1, C-7), 144.24 (d, $^3J_{CF}$, C-9), 172.06 (d, $^2J_{CF}$ 17.4, COO); *m/z* (ASAP) 220 (100 %, [MH]$^+$), 219 (21), 200 (95), 191 (24).

3-Fluoro-6-bromooxindole (8c)

Methyl 2-fluoro-2-(2-nitro-4-bromophenyl)acetate (**6c**, 1.45 g, 5 mmol) gave 3-fluoro-6-bromooxindole (**8c**, 0.65 g, 57%) as a white solid; R_f: 0.18; mp 207-208 °C (decomposes) ([M]$^+$, 228.9534. $C_8H_5{}^{79}BrFNO$ requires: [M]$^+$, 229.9539); IR (neat, cm^{-1}) 3136, 1728, 1617, 1451, 1048; δ_H (d_6-DMSO, 400 MHz) 5.85 (1H, d, $^2J_{HF}$ 50.3, CHF), 7.01 – 7.02 (1H, m, ArH), 7.23 (1H, dm, $^3J_{HH}$ 8.0, ArH), 7.40 (1H, dd, $^3J_{HH}$ 8.0, $^4J_{HH}$ 2.0, ArH), 10.78 (1H, bs, N-H); δ_F (d_6-DMSO, 376 MHz) - 193.44 (d, $^2J_{HF}$ 50.2); δ_C (d_6-DMSO, 100 MHz) 85.49 (d, $^1J_{CF}$ 183.9, CHF), 113.23 (s, C-8), 122.63 (d, $^2J_{CF}$ 16.1, C-4), 124.02 (d, $^5J_{CF}$ 4.0, C-7), 124.85 (d, $^3J_{CF}$ 2.9, C-5), 127.99 (s, C-6), 144.99 (d, $^3J_{CF}$ 5.8, C-9), 172.16 (d, $^2J_{CF}$ 17.4, C=O); *m/z* (ASAP) 232 (92%, [M(^{81}Br)H]$^+$), 230 (100 %, [M(^{79}Br)H]$^+$), 229 (26),212 (86), 210 (91).

3-Fluoro-5-methoxyoxindole (8e)

Methyl 2-fluoro-2-(2-nitro-5-methoxyphenyl)acetate (**6e**, 1.15 g, 5 mmol) gave 3-fluoro-5-methoxyoxindole (**8e**, 0.25 g, 30%) as a tan powder; R_f: 0.20; mp 130-132 °C; ([MH]$^+$, 182.0612. $C_9H_8FNO_2$ requires: [MH]$^+$, 182.0617); IR (neat, cm^{-1}) 3192, 1716, 1486, 1309, 1206, 1050; δ_H (d_6-DMSO, 400 MHz) 3.72 (3H, s, CH_3), 5.84 (1H, d, $^2J_{HF}$ 50.5, CFH), 6.78 (1H, dd, $^3J_{HH}$ 8.5, $^4J_{HH}$ 1,4, ArH), 6.91 (1H, dt, $^3J_{HH}$ 8.5, $^4J_{HH}$ 2.3, ArH), 7.10 (1H, t, $^4J_{HH}$ 2.3,

ArH), 10.45 (1H, bs, N-H); δ_F (d_6-DMSO, 376 MHz) - 193.13 (d, $^2J_{HF}$ 50.5); δ_C (d_6-DMSO, 100 MHz) 55.60 (s, CH_3), 86.40 (d, $^1J_{CF}$ 183.6 Hz, CHF), 110.92 (s, C-7), 112.69 (s, C-5), 116.25 (d, $^4J_{CF}$ 3.8, C-8), 124.37 (d, $^2J_{CF}$ 15.9, C-4), 136.36 (d, $^3J_{CF}$ 5.9, C-9), 155.11 (d, $^4J_{CF}$ 3.3, C-6), 172.21 (d, $^2J_{CF}$ 17.5, C=O); *m/z* (ASAP) 182 (37 %, $[MH]^+$), 162 (100).

Synthesis of fluoromethyl nitroarenes

2,4-Dinitrobenzyl fluoride 5

2-Fluoro-2-(2,4-dinitrophenyl)acetic acid was synthesised according to the general procedure from 2,4-dinitrofluorobenzene (0.93 g, 5 mmol). The crude acid (dark oil) was dissolved in toluene (40 mL) that evaporated (10 mbar) in a 40 °C water bath to leave a dark solid that was purified by column chromatography on silica gel using hexane:ethyl acetate (4:1), R_f: 0.19 as elutant to give 2,4-dinitrobenzyl fluoride **5** (0.61 g, 61%) as an off white solid; mp 68-70 °C (Found: C, 42.1; H, 2.5; N, 13.8. $C_7H_5FN_2O_4$ requires: C, 42.0; H, 2.5; N, 14.0%); ($[MH]^+$, 201.0298. $C_7H_5FN_2O_4$ requires: $[MH]^+$, 201.0312); IR (neat, cm^{-1}) 2360, 1522, 1341, 1027; δ_H ($CDCl_3$, 400 MHz) 5.97 (2H, d, $^2J_{HF}$ 47.8, CFH), 8.08 (1H, d, $^3J_{HH}$ 8.7, ArH), 8.60 (1H, dd, $^3J_{HH}$ 8.7, $^4J_{HH}$ 2.4, ArH), 9.08 (1H, s, ArH); δ_F ($CDCl_3$, 376 MHz) - 219.93 (t, $^2J_{HF}$ 47.8); δ_C ($CDCl_3$, 100 MHz) 81.93 (d, $^1J_{CF}$ 176.4, CH_2F), 120.58 (s, C-3), 128.51 (d, $^3J_{CF}$ 12.7, C-6), 128.56 (d, $^4J_{CF}$ 3.5, C-5), 140.97 (d, $^2J_{CF}$ 19.2, C-1), 145.66 (s, C-4), 147.64 (s, C-2); *m/z* (ASAP) 201 (100 %, $[MH]^+$), 181 (43), 123 (55).

2-Fluoromethyl-3-nitropyridine (7)

Potassium 2-fluoro-2-(3-nitro-2-pyridinyl)acetate (**4f,** 6.00 g, 25 mmol) was suspended in methanol (100 mL), HCl in methanol (1.4 M, 50 mL, 70 mmol) was added and the mixture

was heated at reflux for 17 hours. After cooling, the solvent was evaporated and the solid residue was partitioned between DCM (100 mL) and saturated aq. $NaHCO_3$ (25 mL). The organic layer was washed with saturated brine (25 mL) and dried (Na_2SO_4). After filtration, the solvent was evaporated to leave a brown solid (3.50 g) that was purified by column chromatography on silica gel using hexane:ethyl acetate (2:1, R_f 0.27) as elutant to give 2-fluoromethyl-3-nitropyridine (**7**, 2.66 g, 68%) as an orange crystalline solid; mp 74-76 °C; ($[MH]^+$, 157.0406. $C_6H_5FN_2O_2$ requires: $[MH]^+$, 157.0413); IR (neat, cm^{-1}) 2358, 1598, 1522, 1349, 1022; δ_H ($CDCl_3$, 400 MHz) 5.83 (2H, d, $^2J_{HF}$ 47.3, CFH), 7.53 (1H, dd, $^3J_{HH}$ 8.3, $^3J_{HH}$ 4.8, ArH), 8.41 (1H, dt, $^3J_{HH}$ 8.3, $^4J_{HH}$ 1.2, ArH), 8.88 (1H, dd, $^3J_{HH}$ 4.8, $^4J_{HH}$ 1.5, ArH); δ_F ($CDCl_3$, 376 MHz) - 221.85 (t, $^2J_{HF}$ 47.0); δ_C ($CDCl_3$, 100 MHz) 82.16 (d, $^1J_{CF}$ 175.7, CH_2F), 124.06 (d, $^4J_{CF}$ 1.8, C-6), 132.97 (s, C-5), 144.04 (s, C-4), 151.02 (d, $^2J_{CF}$ 17.1, C-2), 153.47 (s, C-3); *m/z* (ASAP) 157 (100%, $[MH]^+$), 137 (46). Crystals suitable for X-ray crystallography were grown by slow evaporation of chloroform.

References

1. Zhang, F.; Song, J. Z., *Tetrahedron Lett.* **2006**, *47*, 7641–7644.
