# Supplementary material for: Syntheses of fluorooxindole and 2-fluoro-2-arylacetic acid derivatives from diethyl 2-fluoromalonate ester
Source: Beilstein J Org Chem. 2014 May 22;10:1213–9. doi: 10.3762/bjoc.10.119 (PMC4077372; doi:10.3762/bjoc.10.119)
Supplement: File 2 — NMR spectra. [file Beilstein_J_Org_Chem-10-1213-s002.pdf]

Supporting Information File 2

for

Syntheses of fluorooxindole and 2-fluoro-2-arylacetic acid derivatives from diethyl 2-fluoromalonate ester

Antal Harsanyi[1], Graham Sandford[1*], Dmitri S. Yufit[2] and Judith A.K. Howard[2]

Address: [1]Department of Chemistry, Durham University, South Road, Durham, DH1 3LE, U.K, [2]Chemical Crystallography, Department of Chemistry, Durham University, South Road, Durham, DH1 3LE, U.K.

Email: Graham Sandford - graham.sandford@durham.ac.uk

*Corresponding author

NMR spectra

Diethyl 2-fluoro-2-(2-nitrophenyl)malonate (**3**) S3

2-Fluoro-2-(2-nitrophenyl)acetic acid (**4a**) S5

2-Fluoro-2-(2-nitro-4-trifluoromethylphenyl)acetic acid (**4b**) S7

2-Fluoro-2-(2-nitro-4-bromophenyl)acetic acid (**4c**) S9

2-Fluoro-2-(2,4-dinitrophenyl)acetic acid **(4d)** S11

2-Fluoro-2-(2-nitro-5-methoxyphenyl)acetic acid (**4e**) S13

Potassium 2-fluoro-2-(3-nitro-2-pyridinyl)acetate (**4f**) S15

Methyl 2-fluoro-2-(2-nitrophenyl)acetate (**6a**) S17

Methyl 2-fluoro-2-(2-nitro-4-trifluoromethylphenyl)acetate (**6b**) S19

Methyl 2-fluoro-2-(2-nitro-4-bromophenyl)acetate (**6c**) S21

Methyl 2-fluoro-2-(2,4-dinitrophenyl)acetate (**6d**) S23

Methyl 2-fluoro-2-(2-nitro-5-methoxyphenyl)acetate (**6e**) S25

3-Fluorooxindole (**8a**) S27

3-Fluoro-6-trifluoromethyloxindole (**8b**) S29

3-Fluoro-6-bromooxindole (**8c**) S31

3-Fluoro-5-methoxyoxindole (**8e**) S33

2,4-Dinitrobenzyl fluoride **5** **S**35

2-Fluoromethyl-3-nitropyridine (**7**) S37

Diethyl 2-fluoro-2-(2-nitrophenyl)malonate (**3**)

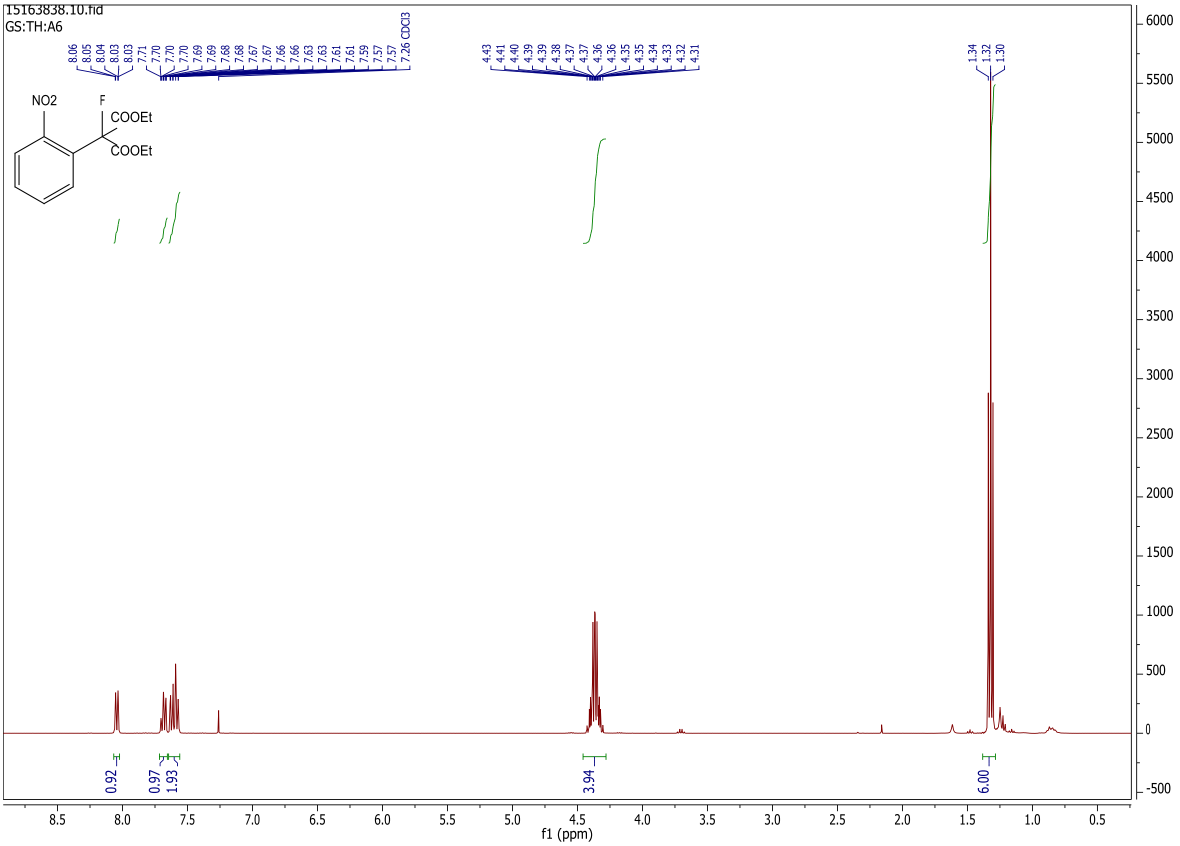

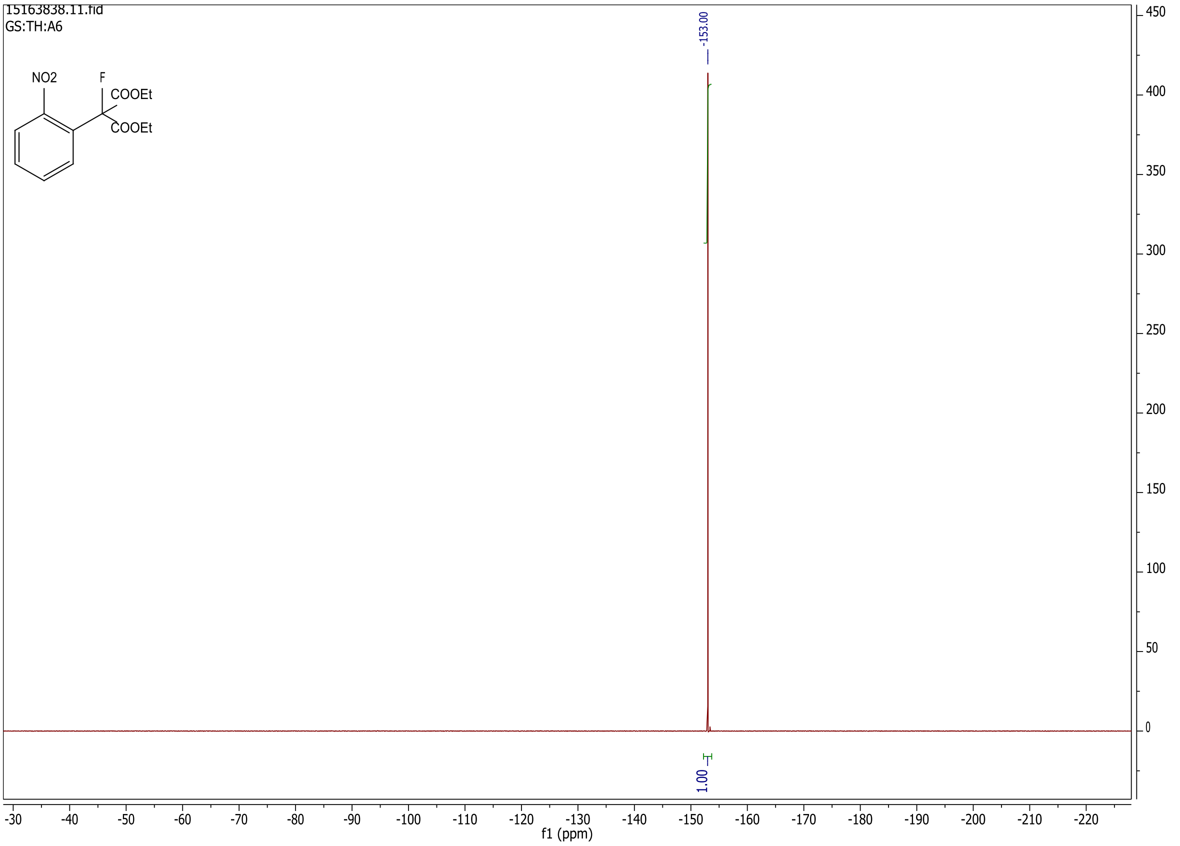

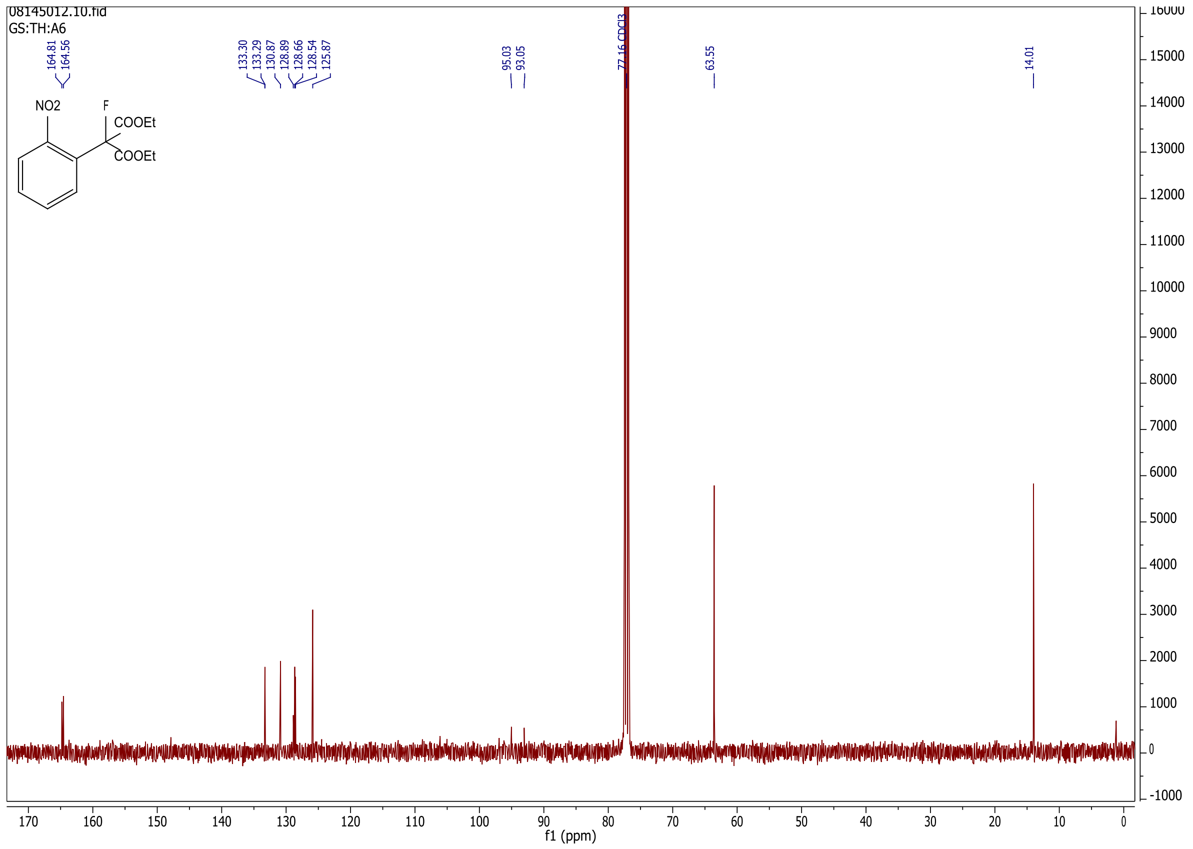

08145012.10.fid
GS:TH:A6
164.81
164.56
133.30
133.29
130.87
128.89
128.66
128.54
125.87
95.03
93.05
77.16 CDCl3
63.55
14.01
NO2
F
COOEt
COOEt
f1 (ppm)

2-Fluoro-2-(2-nitrophenyl)acetic acid (**4a**)

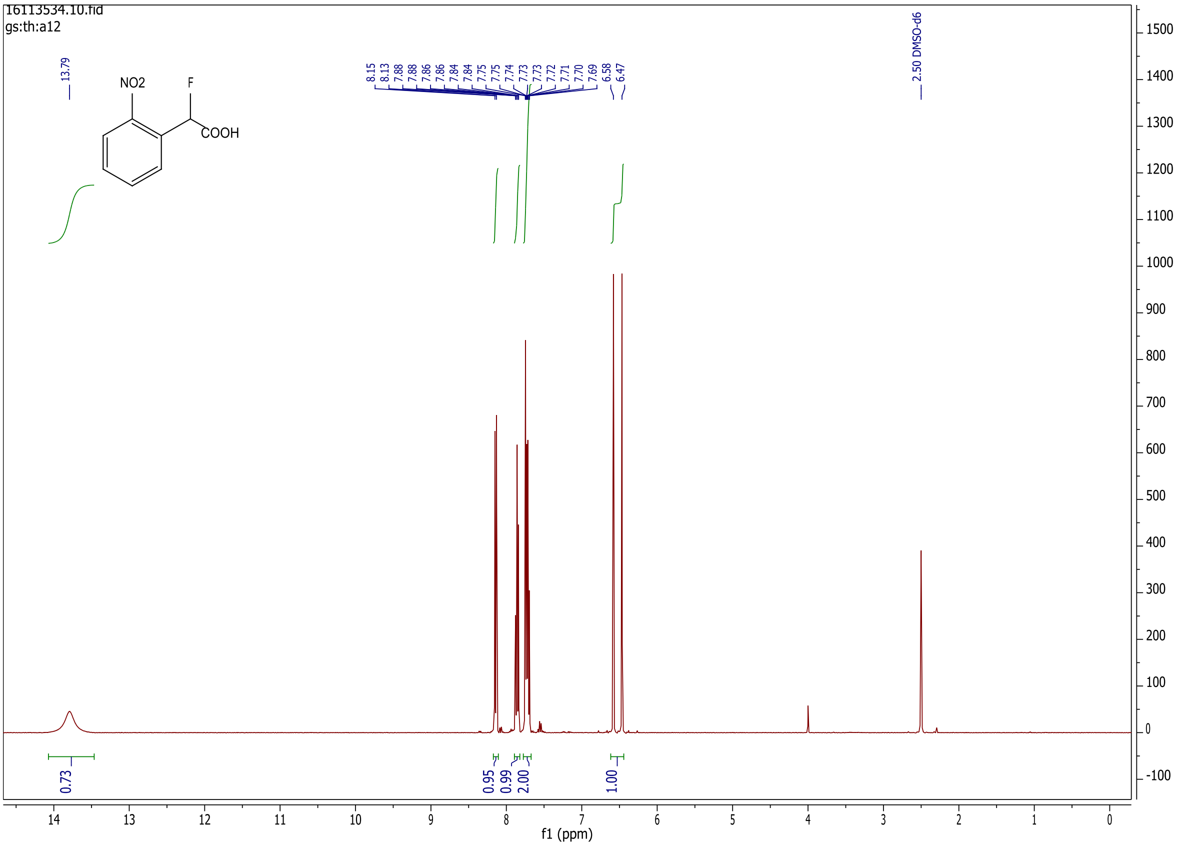

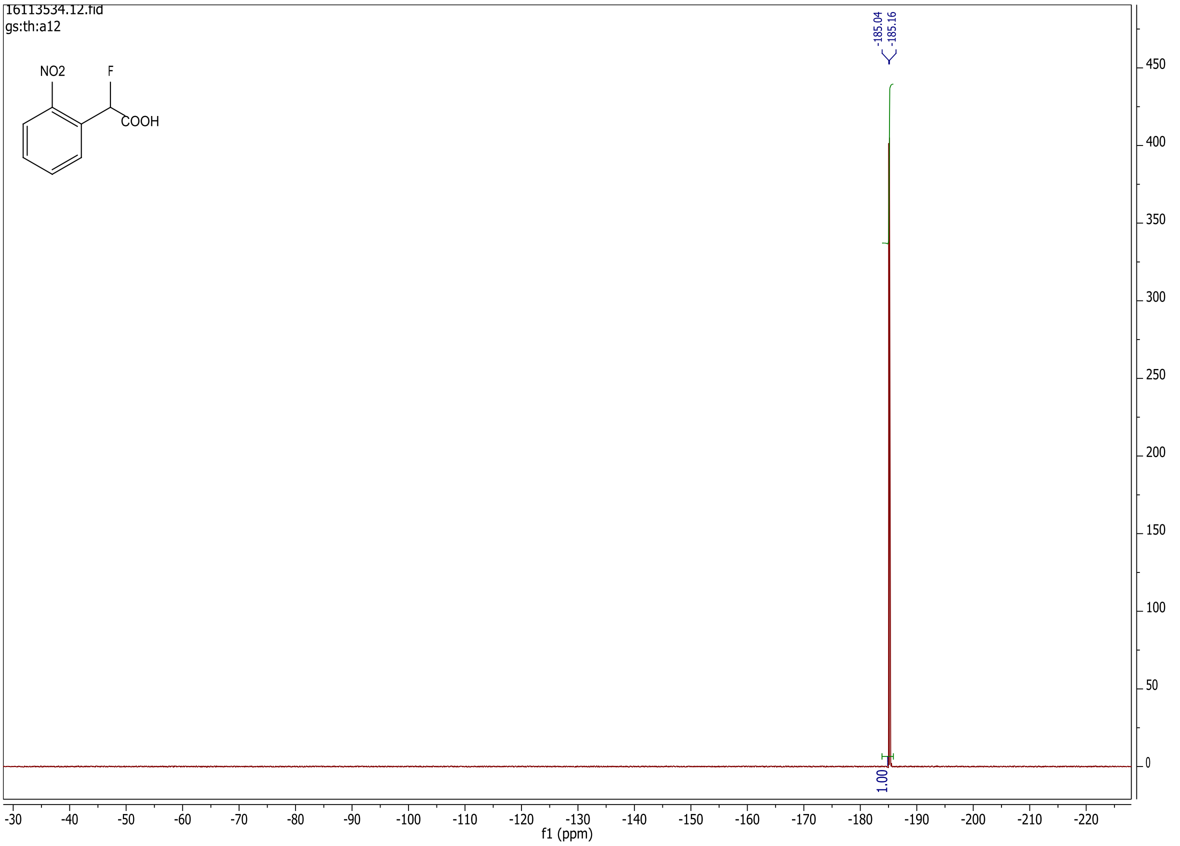

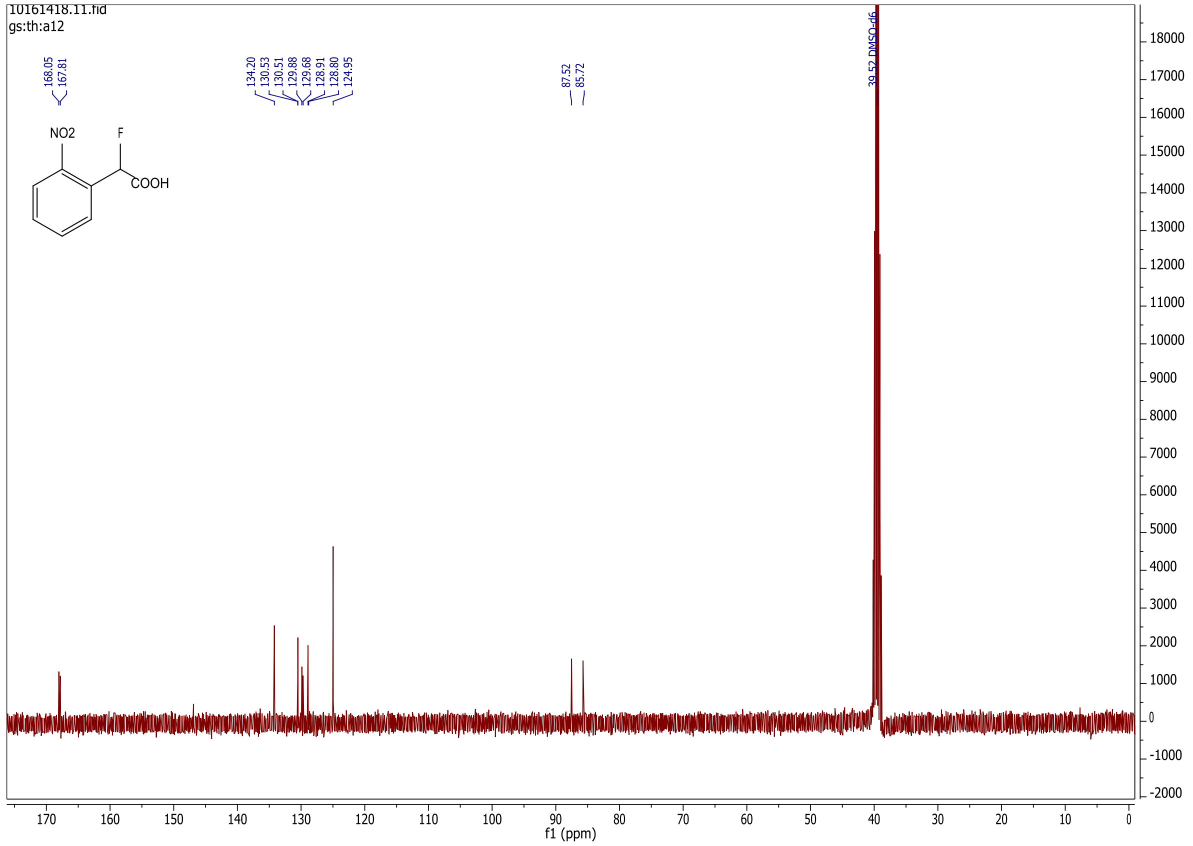

10161418.11.fid
gs:th:a12
168.05
167.81
134.20
130.53
130.51
129.88
129.68
128.91
128.80
124.95
87.52
85.72
39.52 DMSO-d6
NO2
F
COOH
f1 (ppm)

2-Fluoro-2-(2-nitro-4-trifluoromethylphenyl)acetic acid (**4b**)

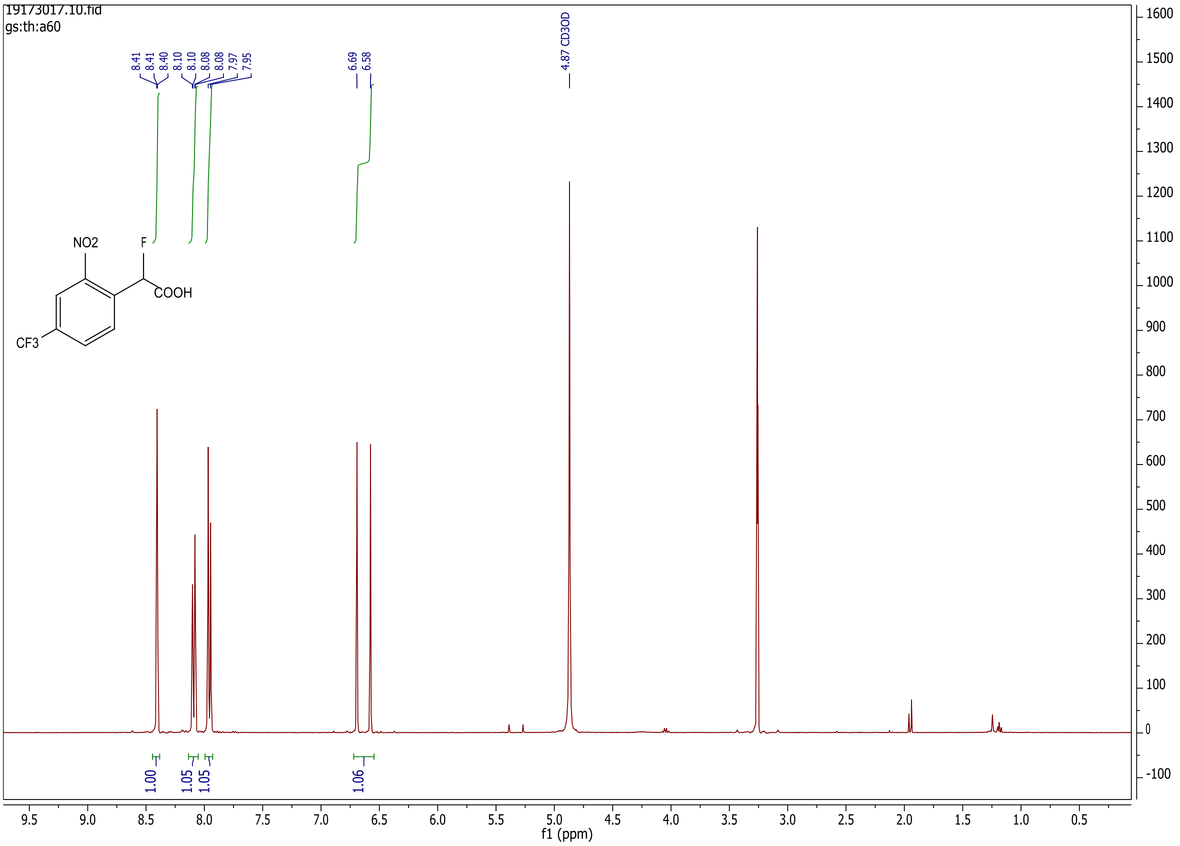

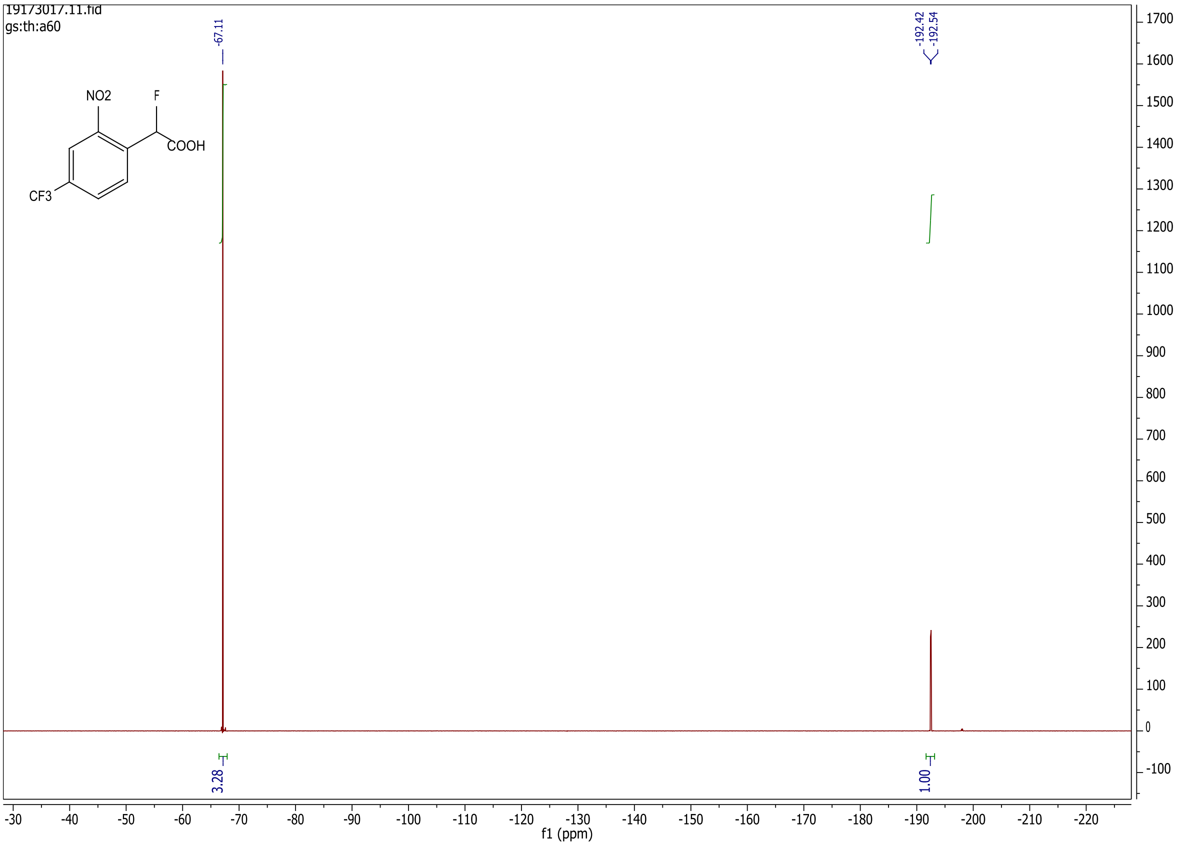

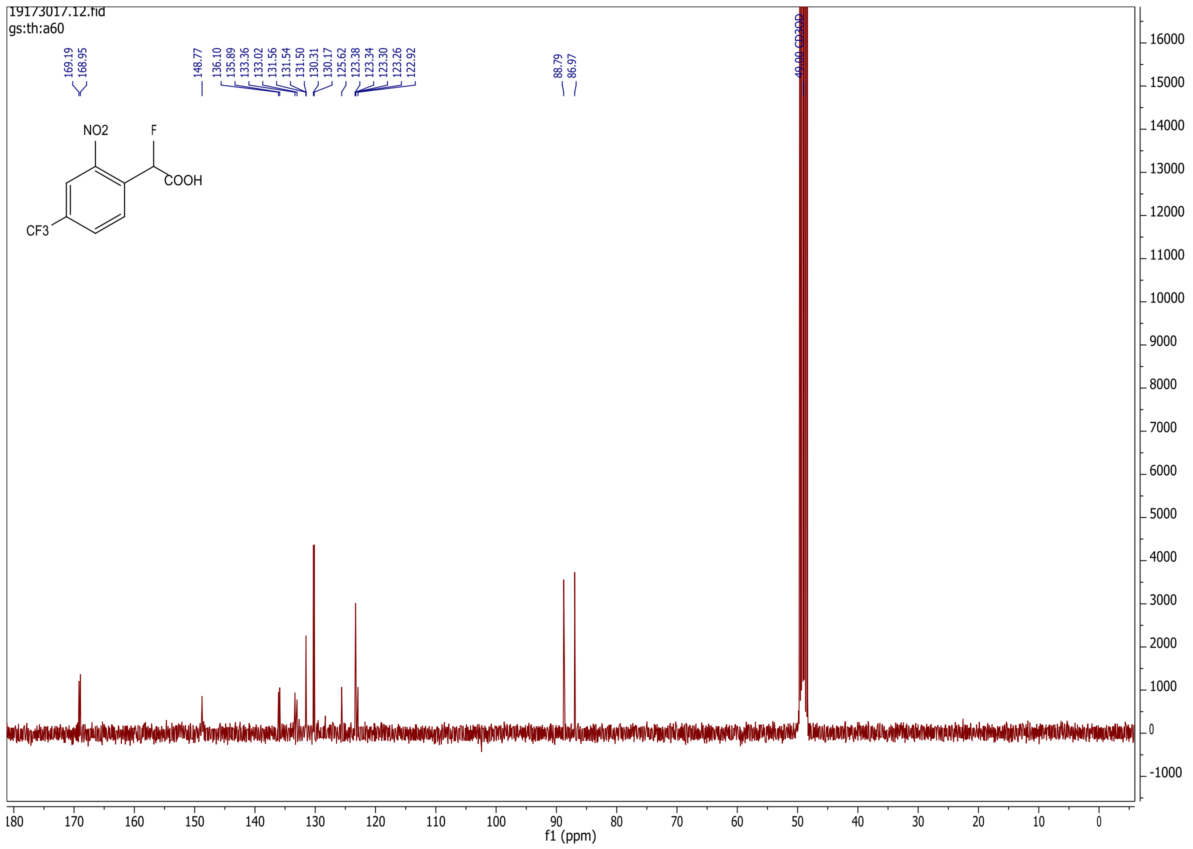

19173017.12.fid
gs:th:a60
169.19
168.95
148.77
136.10
135.89
133.36
133.02
131.56
131.54
131.50
130.31
130.17
125.62
123.38
123.34
123.30
123.26
122.92
88.79
86.97
49.00 CD3OD
NO2
F
COOH
CF3
f1 (ppm)

2-Fluoro-2-(2-nitro-4-bromophenyl)acetic acid (**4c**)

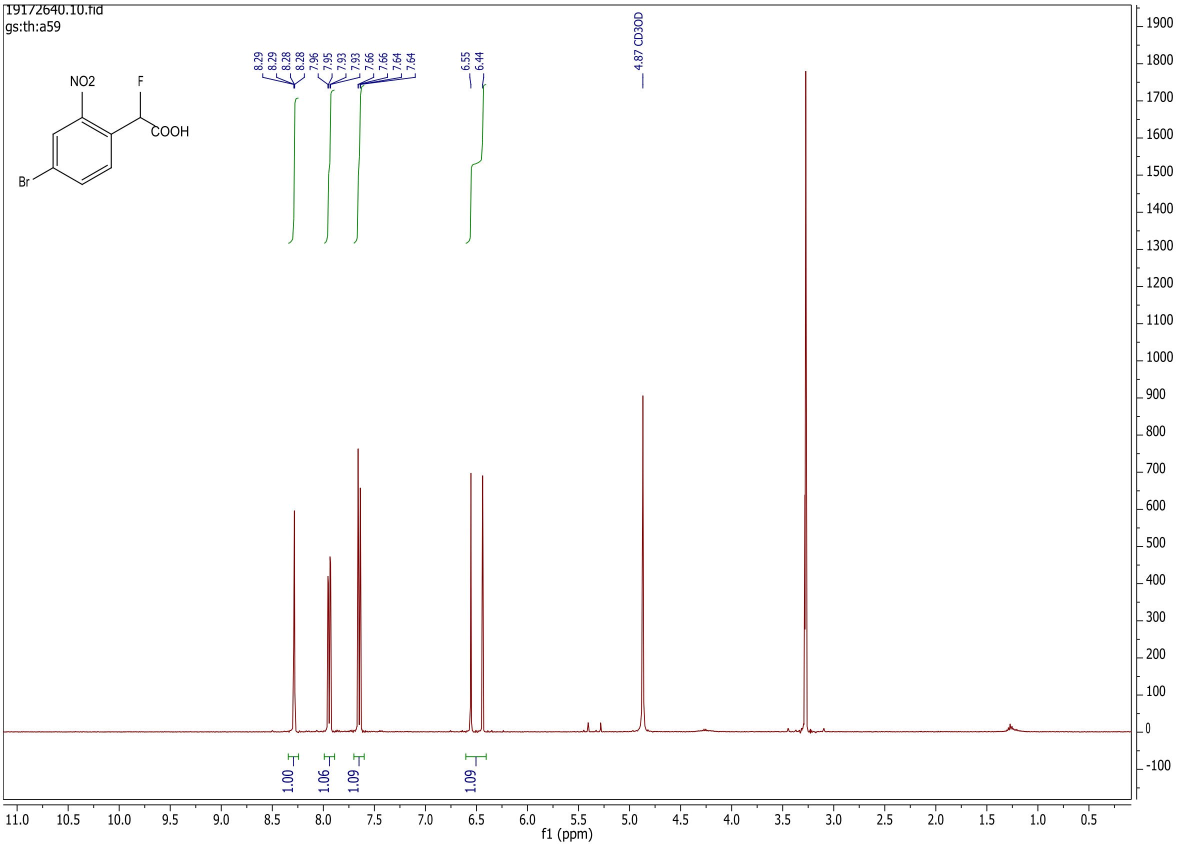

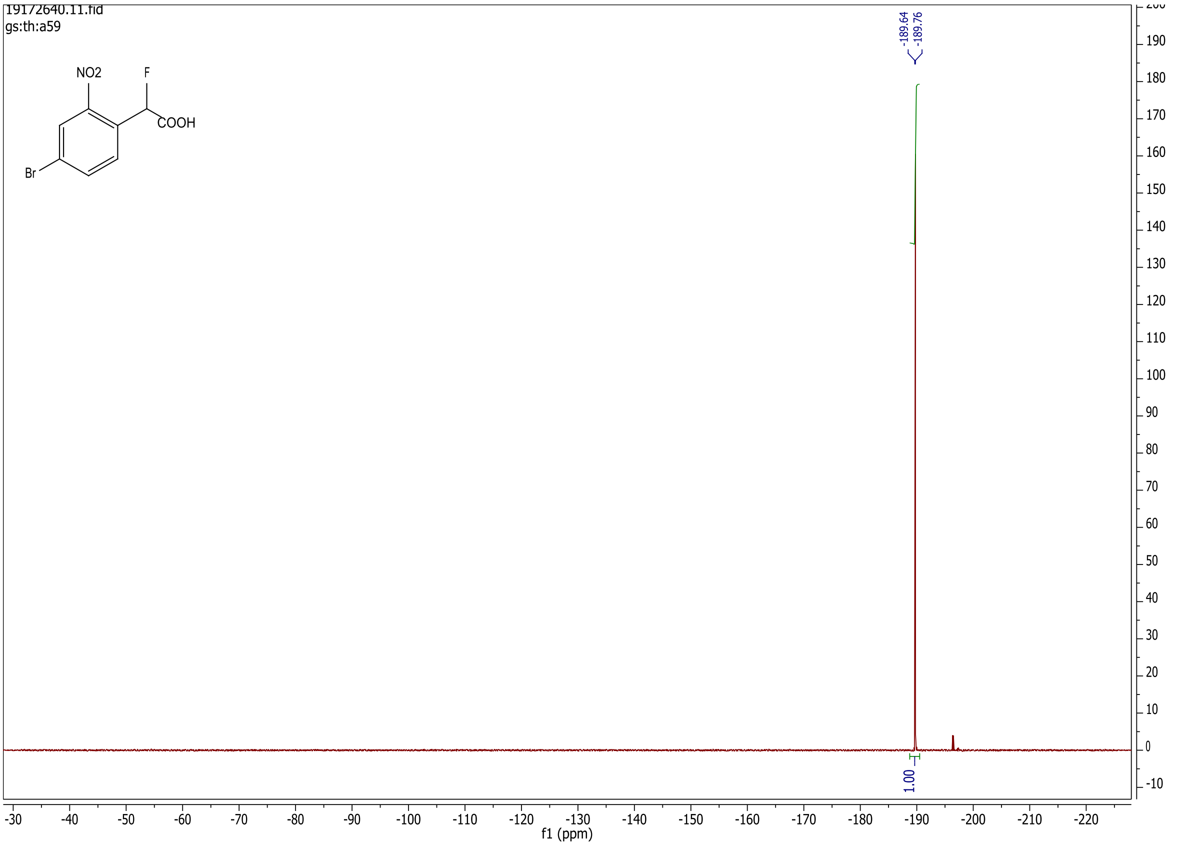

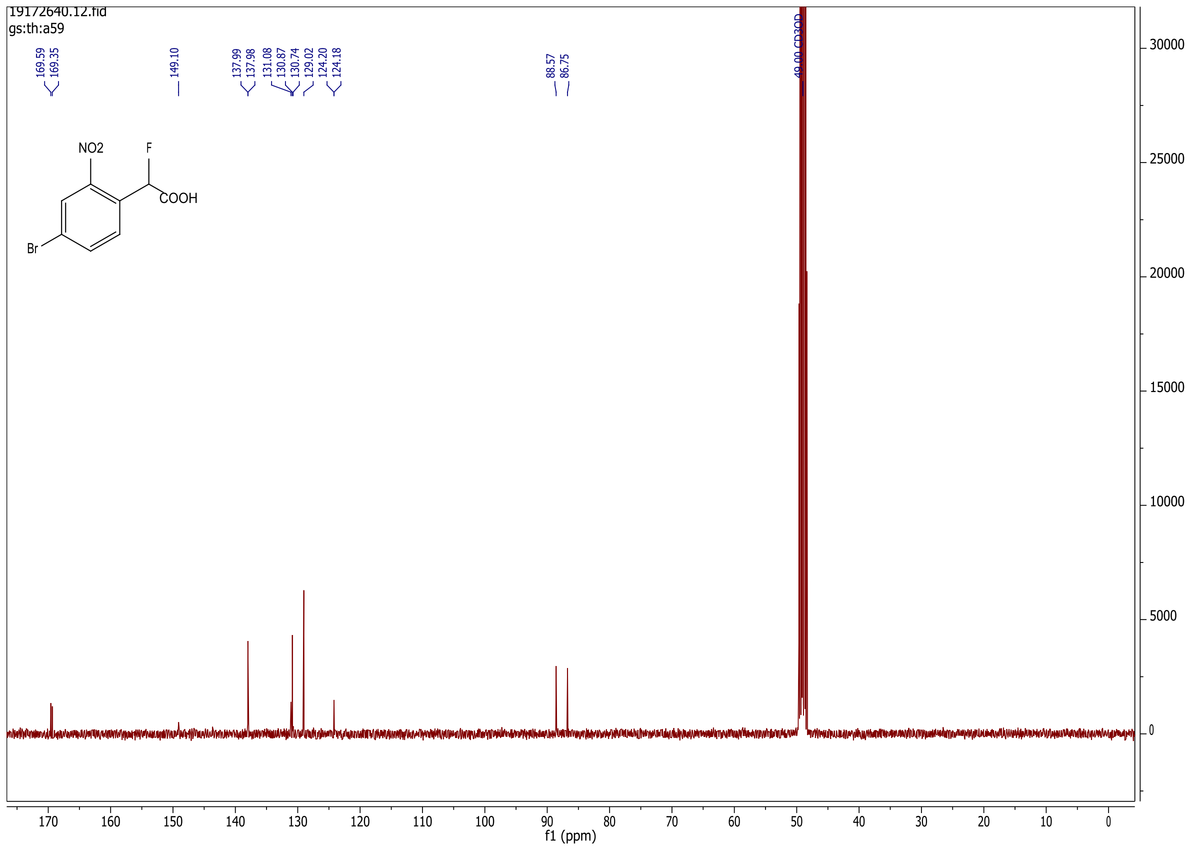

19172640.12.fid
gs:th:a59
169.59
169.35
149.10
137.99
137.98
131.08
130.87
130.74
129.02
124.20
124.18
88.57
86.75
49.00 CD3OD
NO2
F
COOH
Br
30000
25000
20000
15000
10000
5000
0
170
160
150
140
130
120
110
100
90
80
70
60
50
40
30
20
10
0
f1 (ppm)

2-Fluoro-2-(2,4-dinitrophenyl)acetic acid (**4d**)

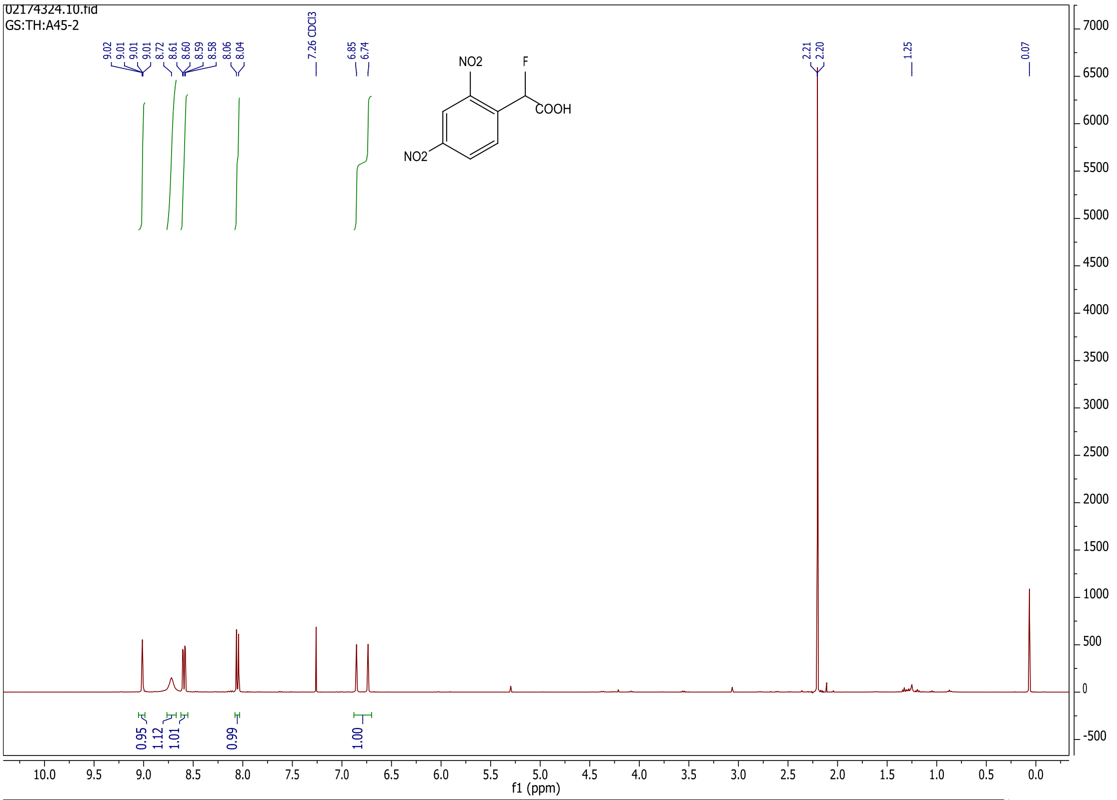

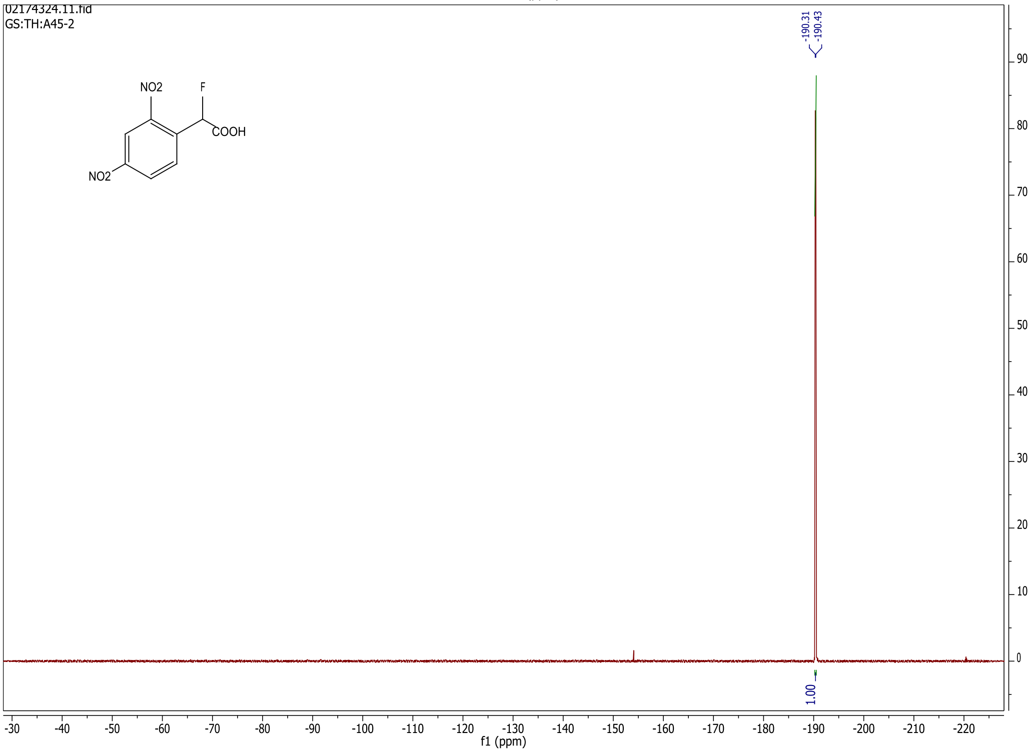

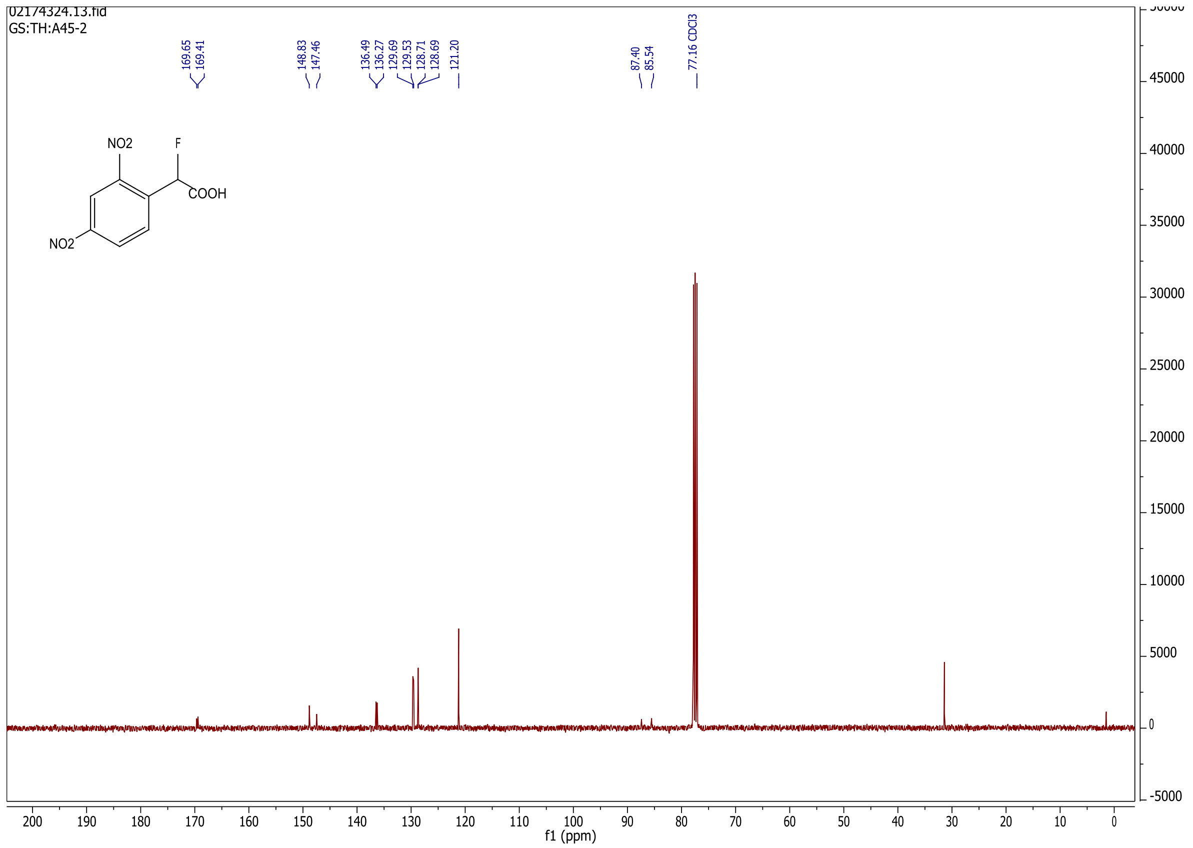
02174324.13.fid
GS:TH:A45-2
169.65
169.41
148.83
147.46
136.49
136.27
129.69
129.53
128.71
128.69
121.20
87.40
85.54
77.16 CDCl3
NO2
F
COOH
NO2
f1 (ppm)

2-Fluoro-2-(2-nitro-5-methoxyphenyl)acetic acid (**4e**)

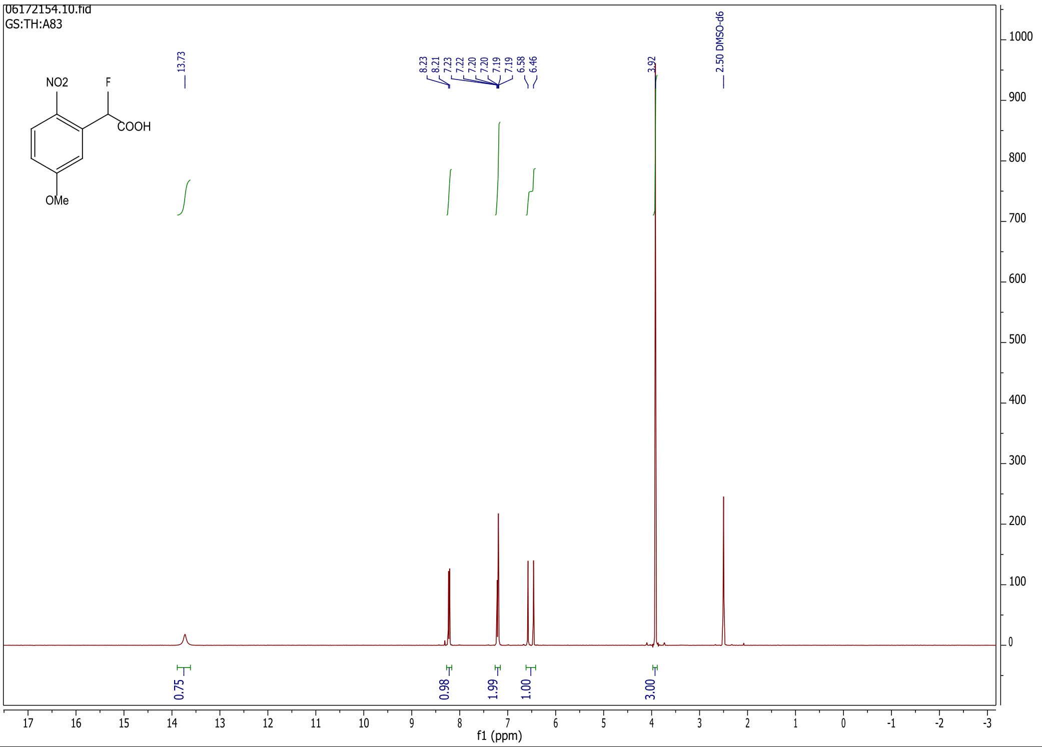

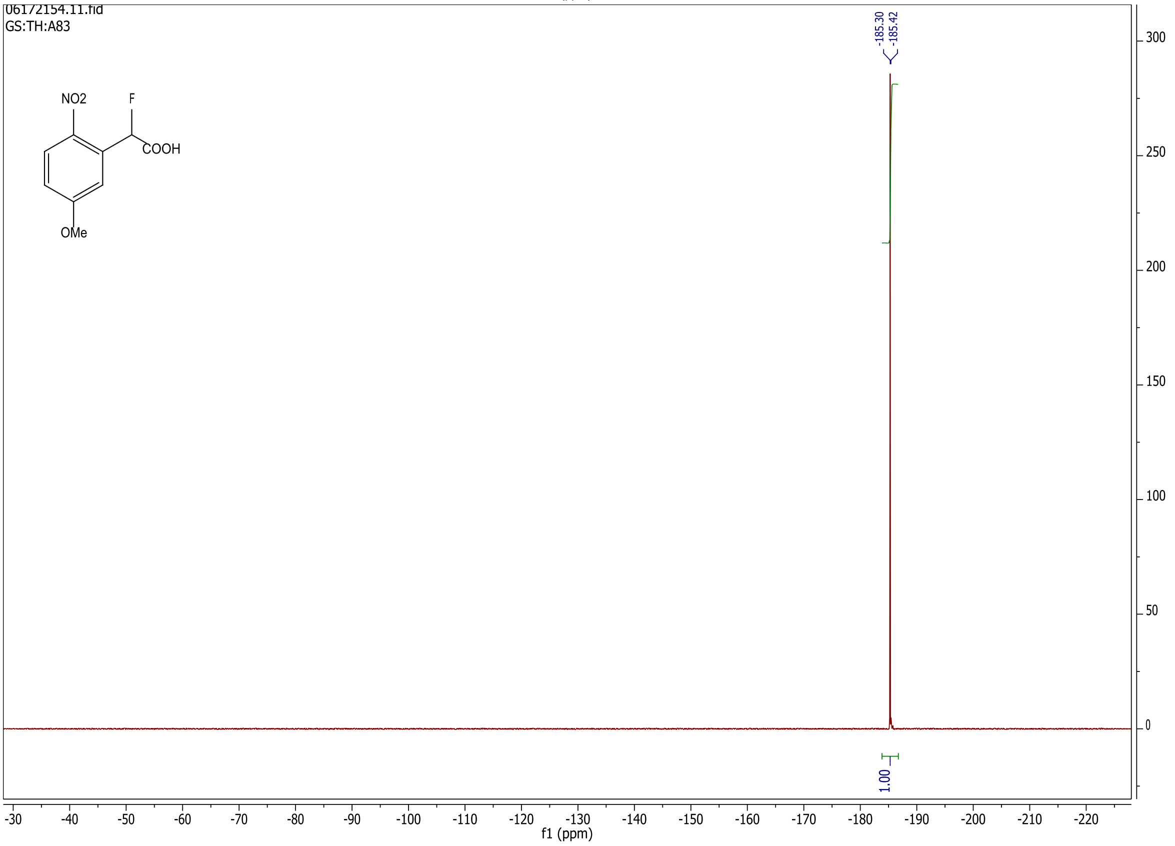

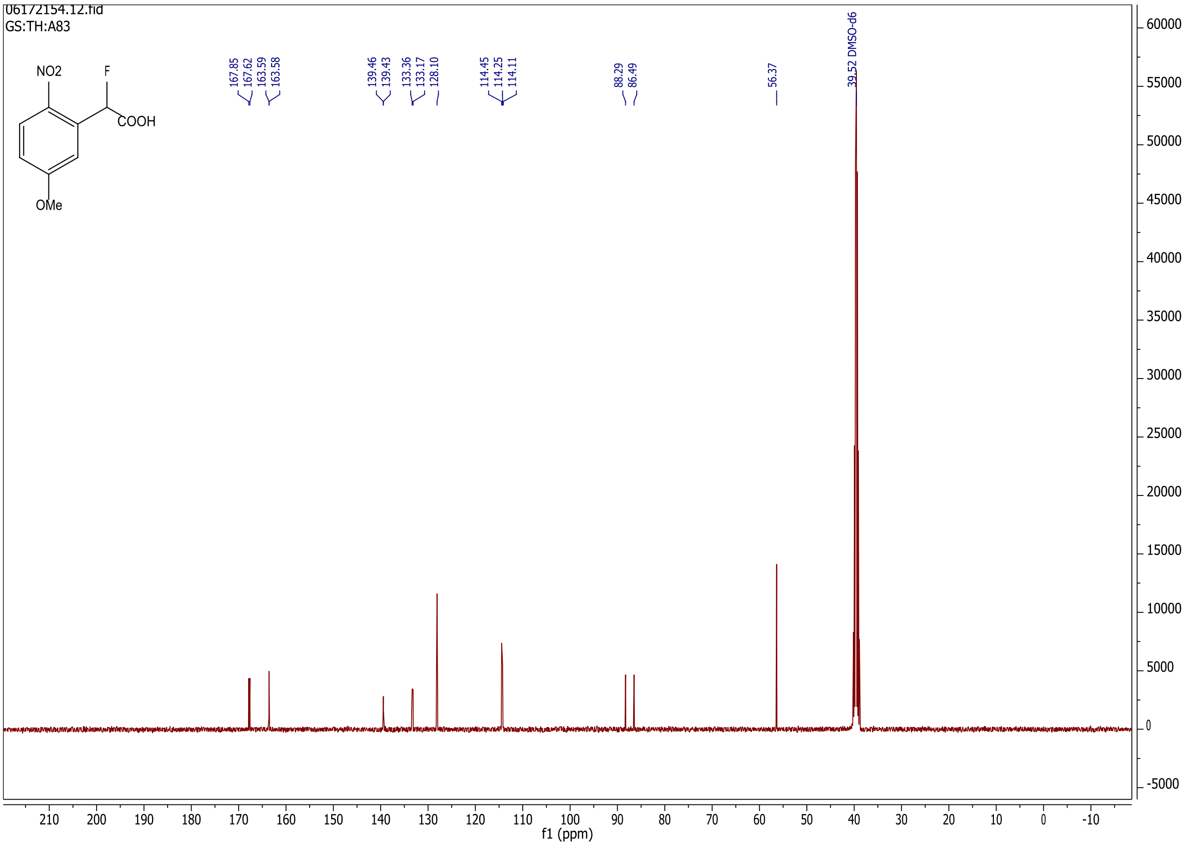
06172154.12.fid
GS:TH:A83
NO2
F
COOH
OMe
167.85
167.62
163.59
163.58
139.46
139.43
133.36
133.17
128.10
114.45
114.25
114.11
88.29
86.49
56.37
39.52 DMSO-d6
f1 (ppm)

Potassium 2-fluoro-2-(3-nitro-2-pyridinyl)acetate (**4f**)

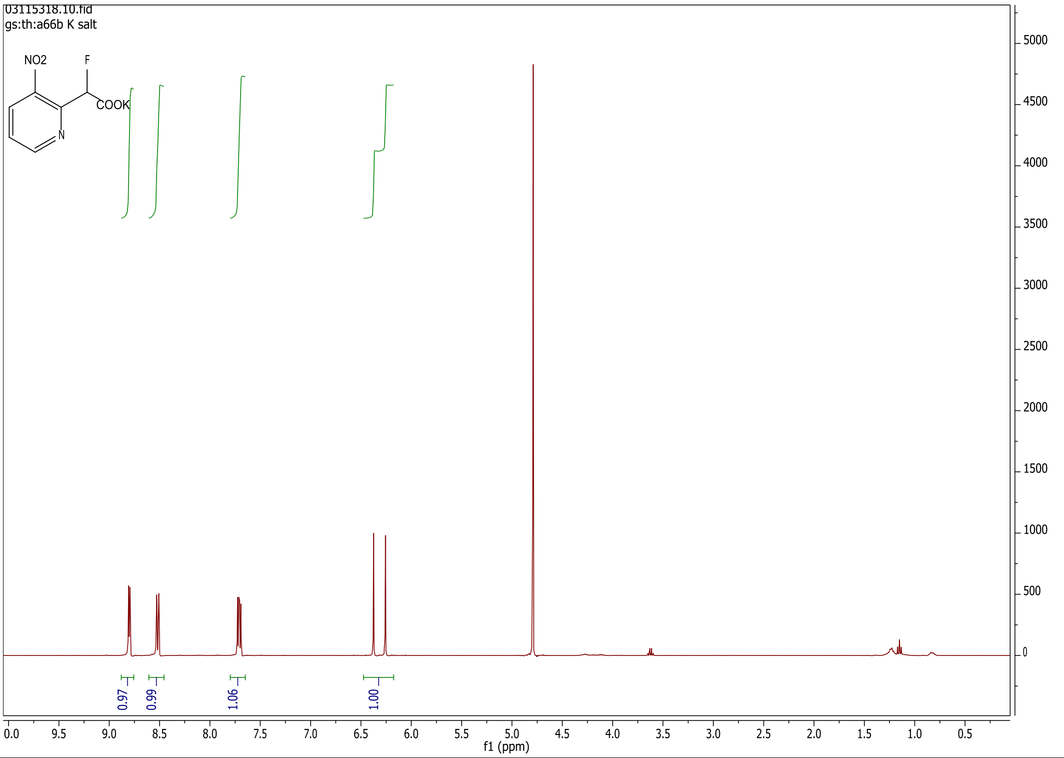

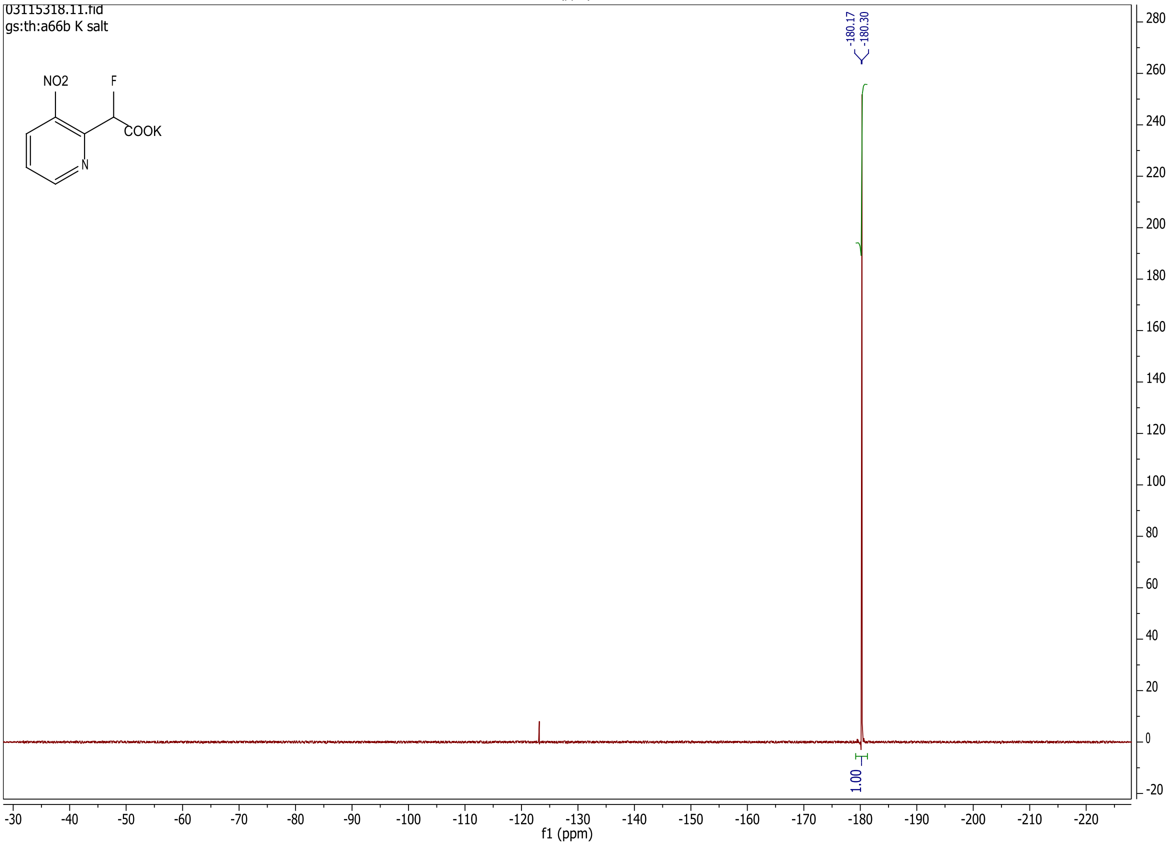

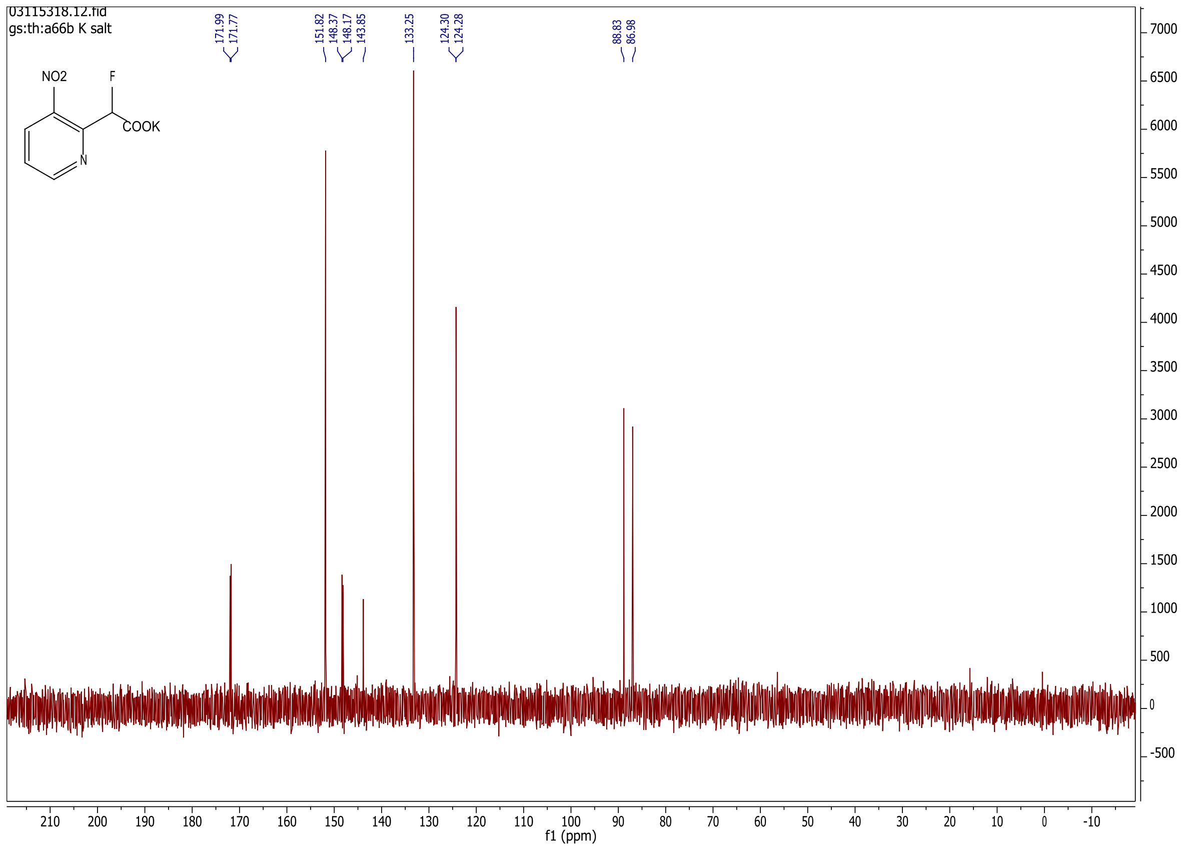
03115318.12.fid
gs:th:a66b K salt
NO2
F
COOK
N
171.99
171.77
151.82
148.37
148.17
143.85
133.25
124.30
124.28
88.83
86.98
f1 (ppm)

Methyl 2-fluoro-2-(2-nitrophenyl)acetate (**6a**)

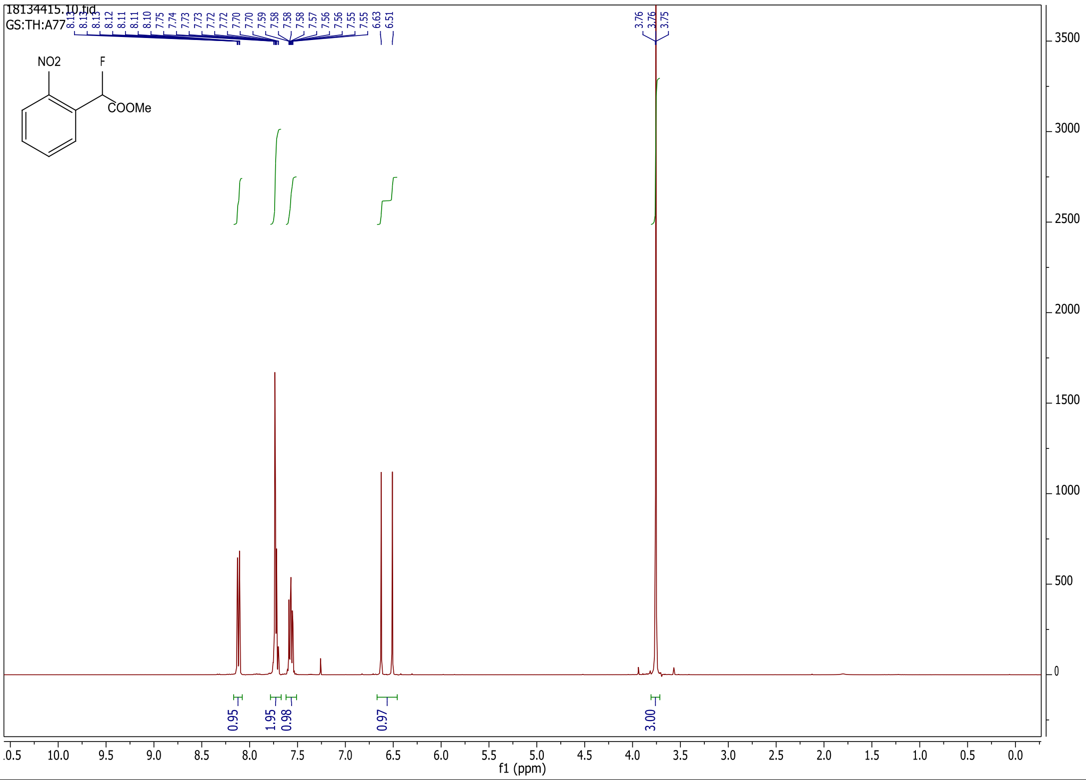

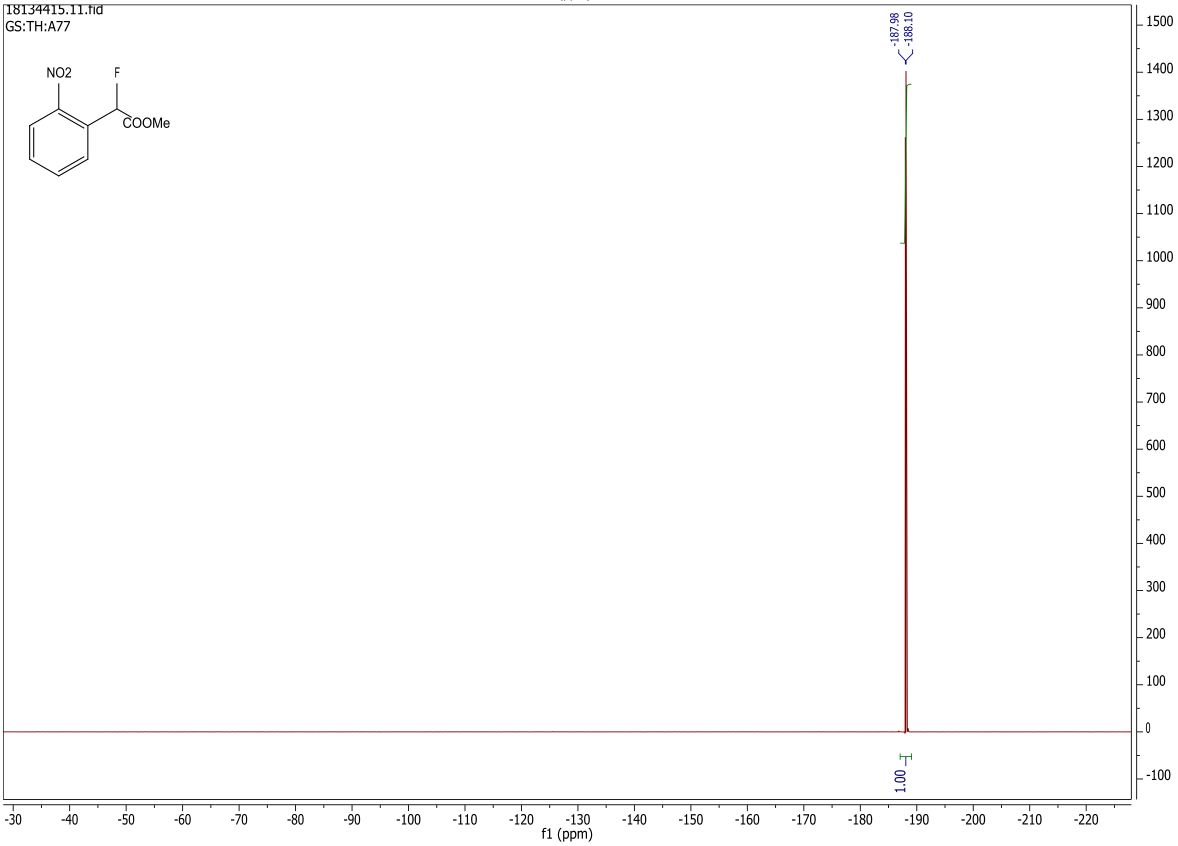

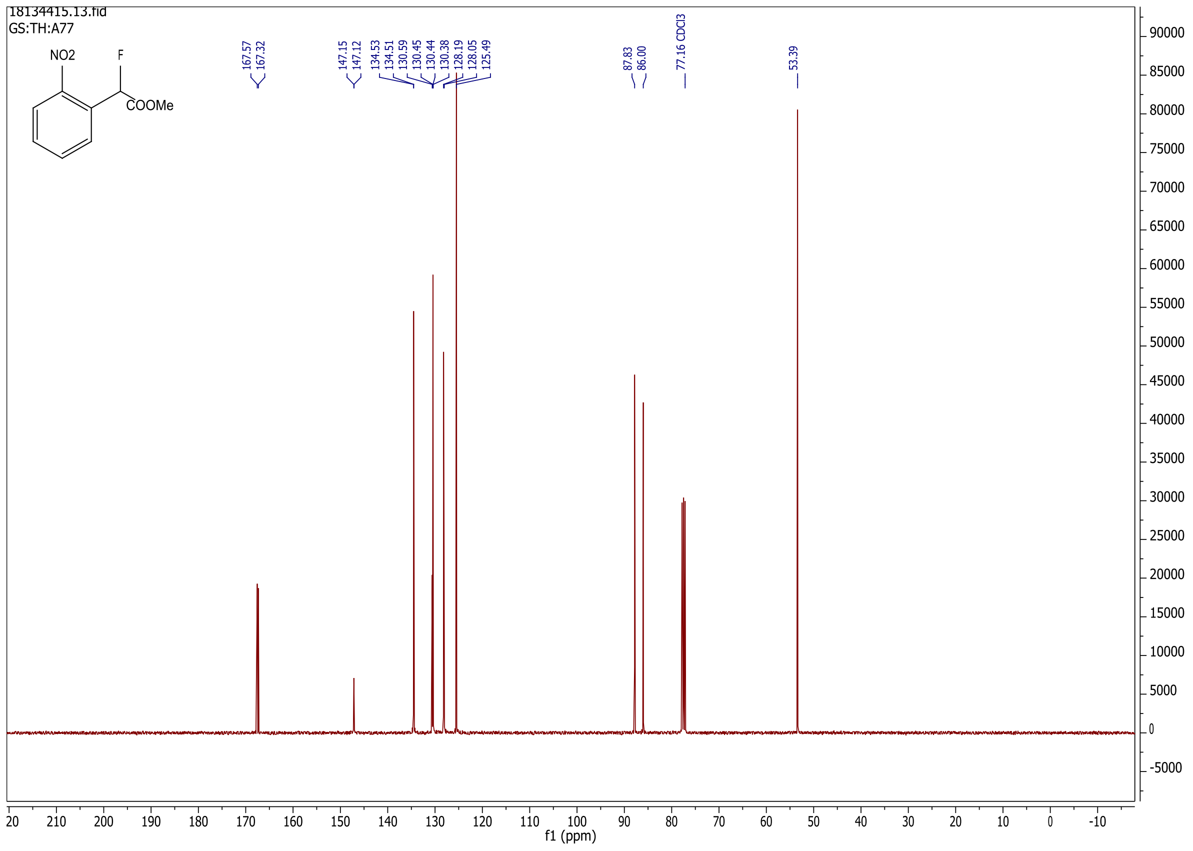
18134415.13.fid
GS:TH:A77
NO2
F
COOMe
167.57
167.32
147.15
147.12
134.53
134.51
130.59
130.45
130.44
130.38
128.19
128.05
125.49
87.83
86.00
77.16 CDCl3
53.39
f1 (ppm)

Methyl 2-fluoro-2-(2-nitro-4-trifluoromethylphenyl)acetate (**6b**)

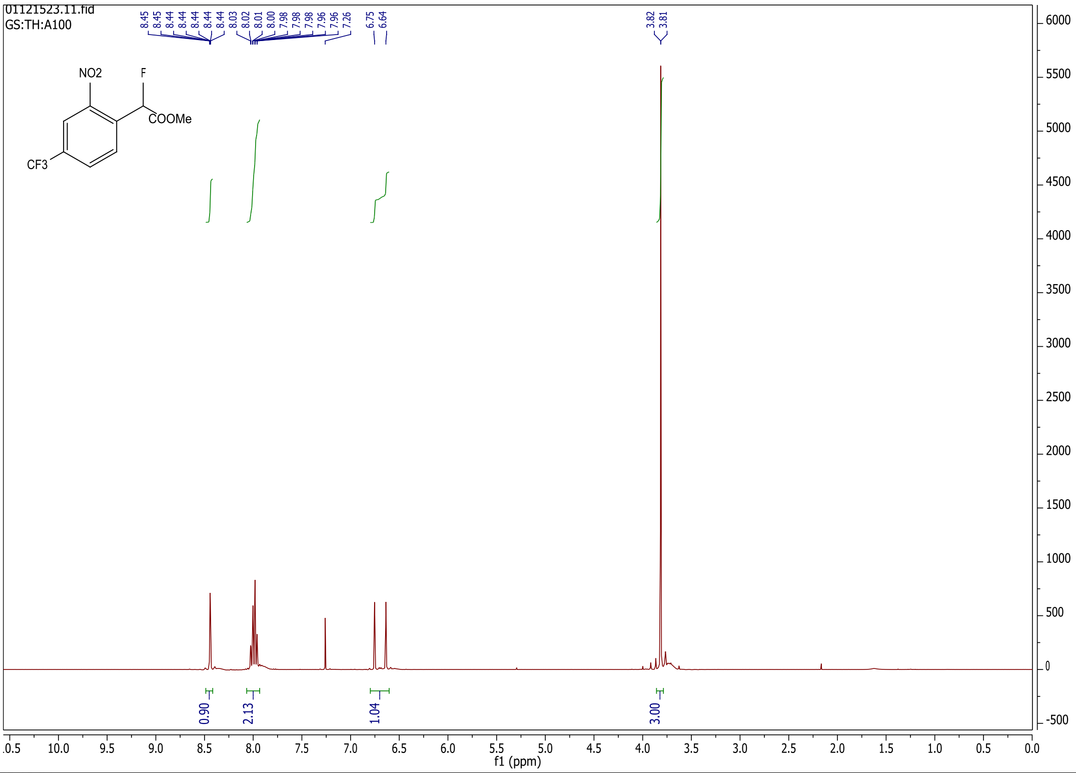

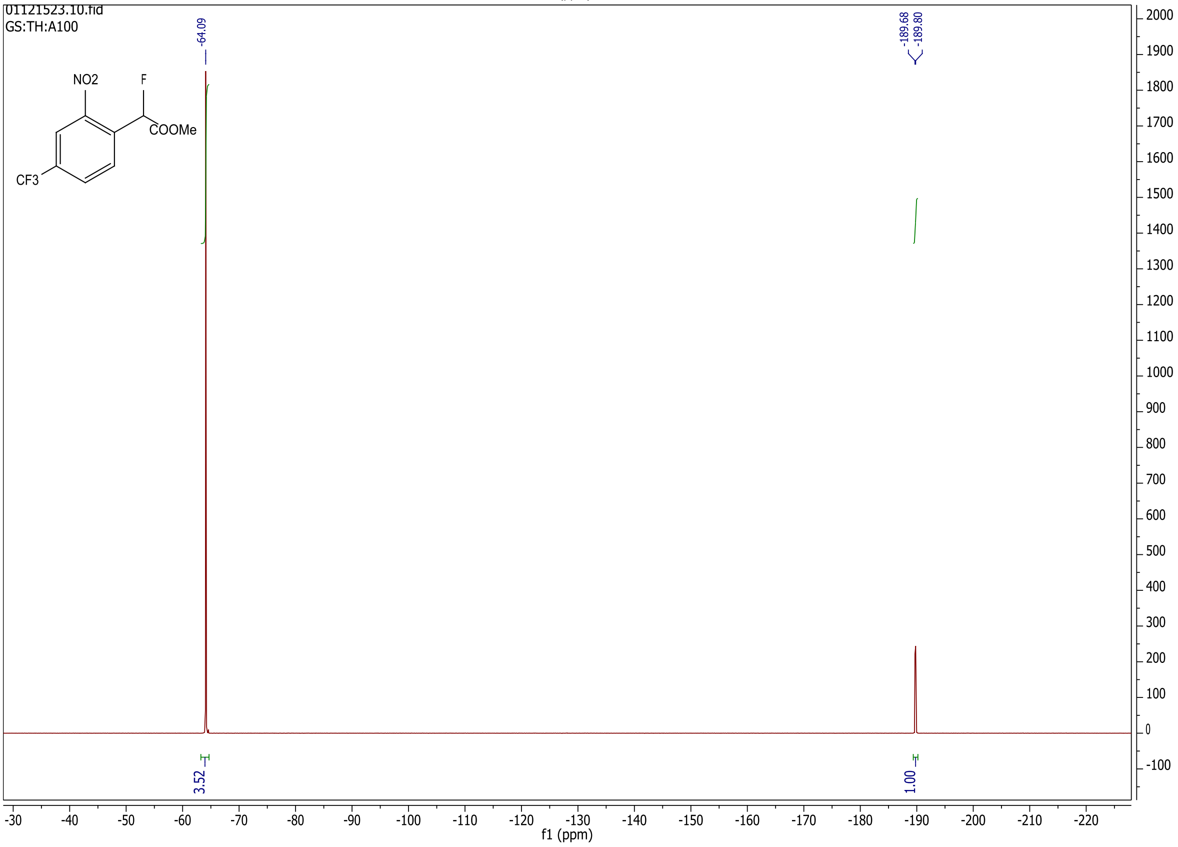

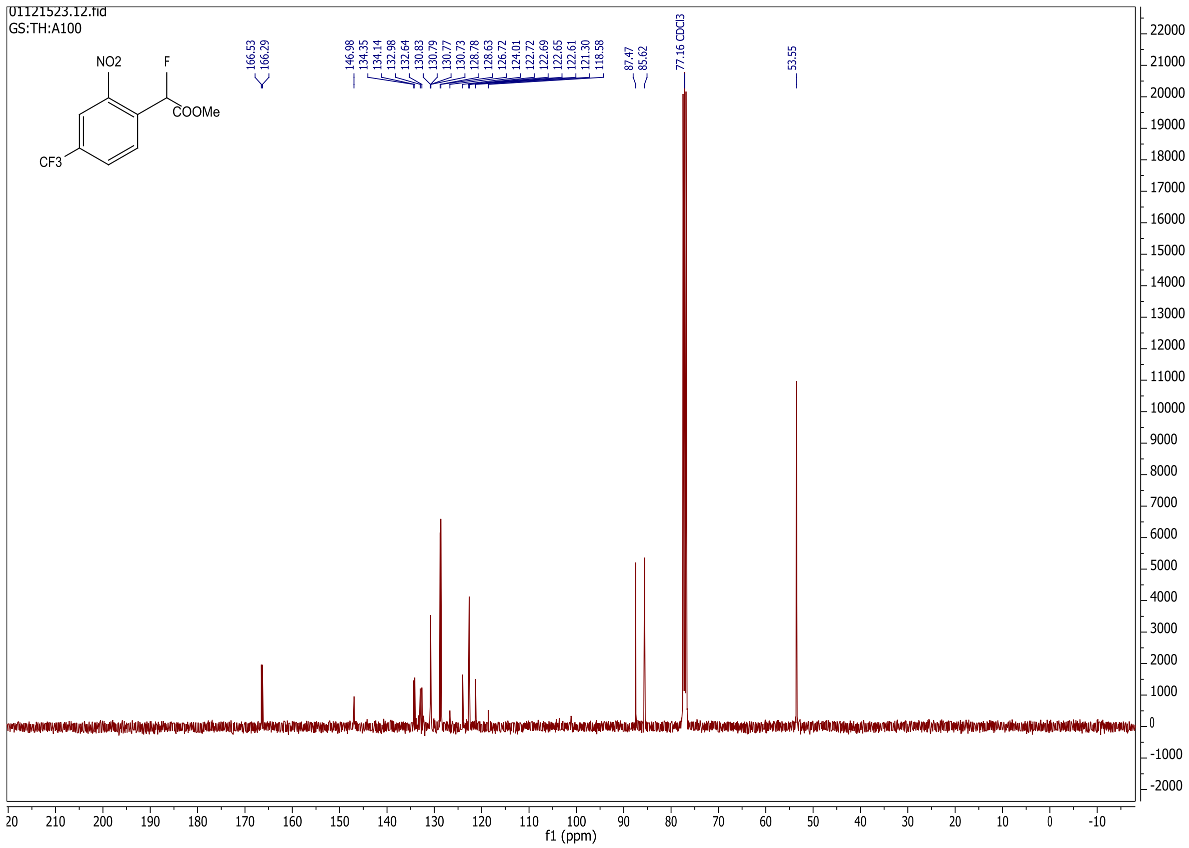
01121523.12.fid
GS:TH:A100
NO2
F
COOMe
CF3
166.53
166.29
146.98
134.35
134.14
132.98
132.64
130.83
130.79
130.77
130.73
128.78
128.63
126.72
124.01
122.72
122.69
122.65
122.61
121.30
118.58
87.47
85.62
77.16 CDCl3
53.55
f1 (ppm)

Methyl 2-fluoro-2-(2-nitro-4-bromophenyl)acetate (**6c**)

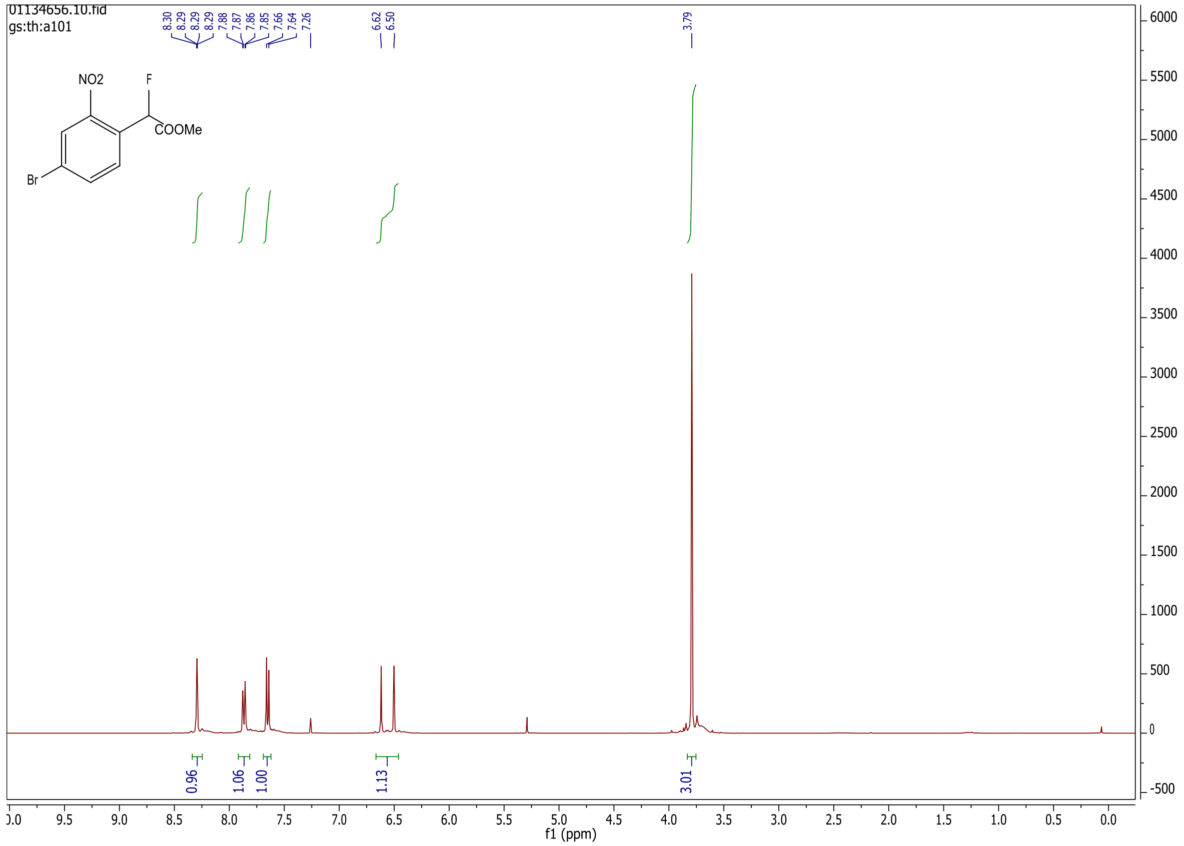

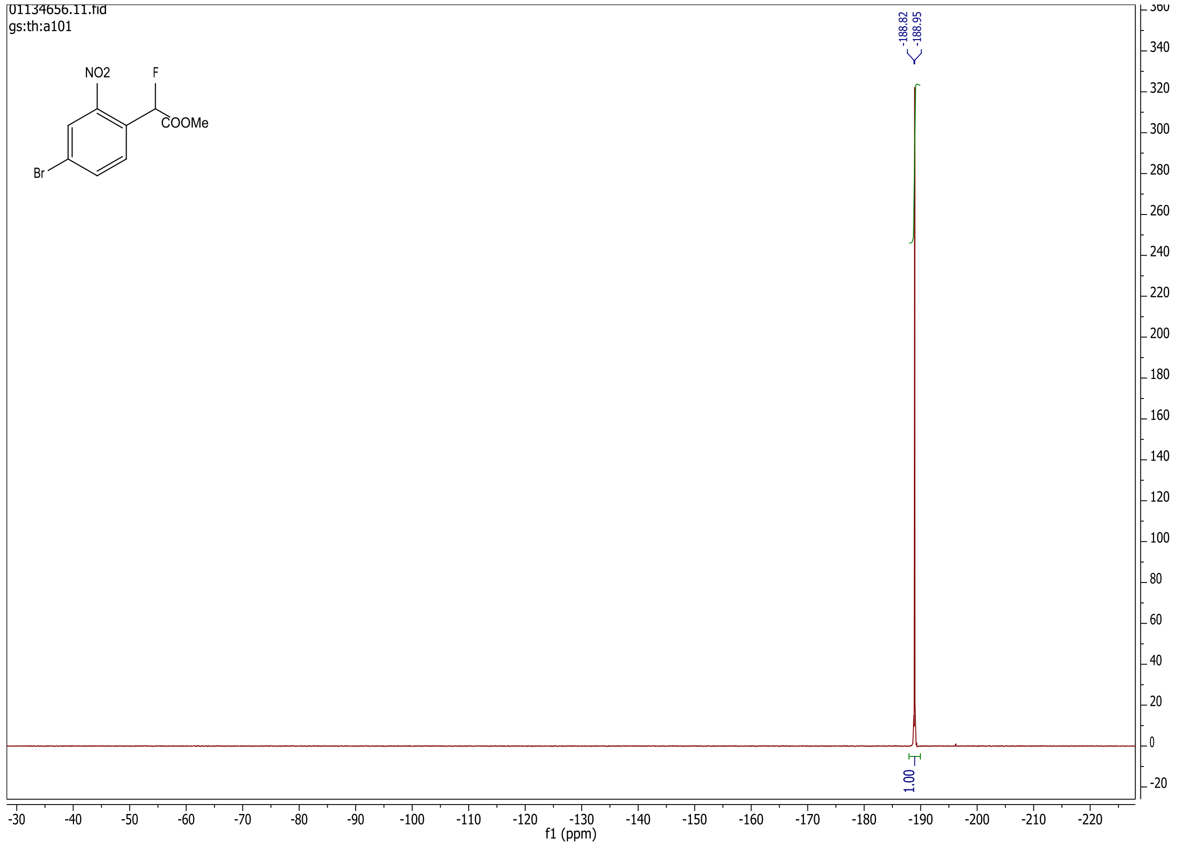

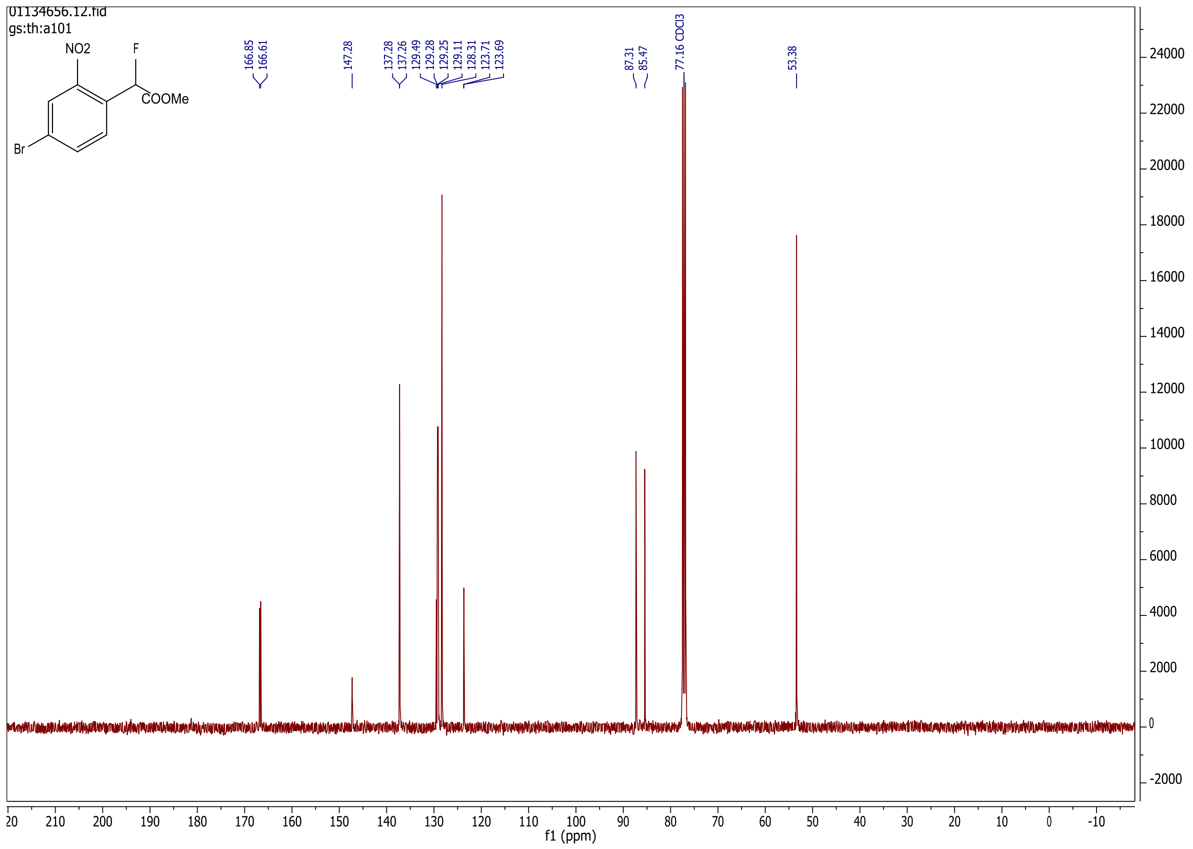
01134656.12.fid
gs:th:a101
NO2
F
COOMe
Br
166.85
166.61
147.28
137.28
137.26
129.49
129.28
129.25
129.11
128.31
123.71
123.69
87.31
85.47
77.16 CDCl3
53.38
f1 (ppm)

Methyl 2-fluoro-2-(2,4-dinitrophenyl)acetate (**6d**)

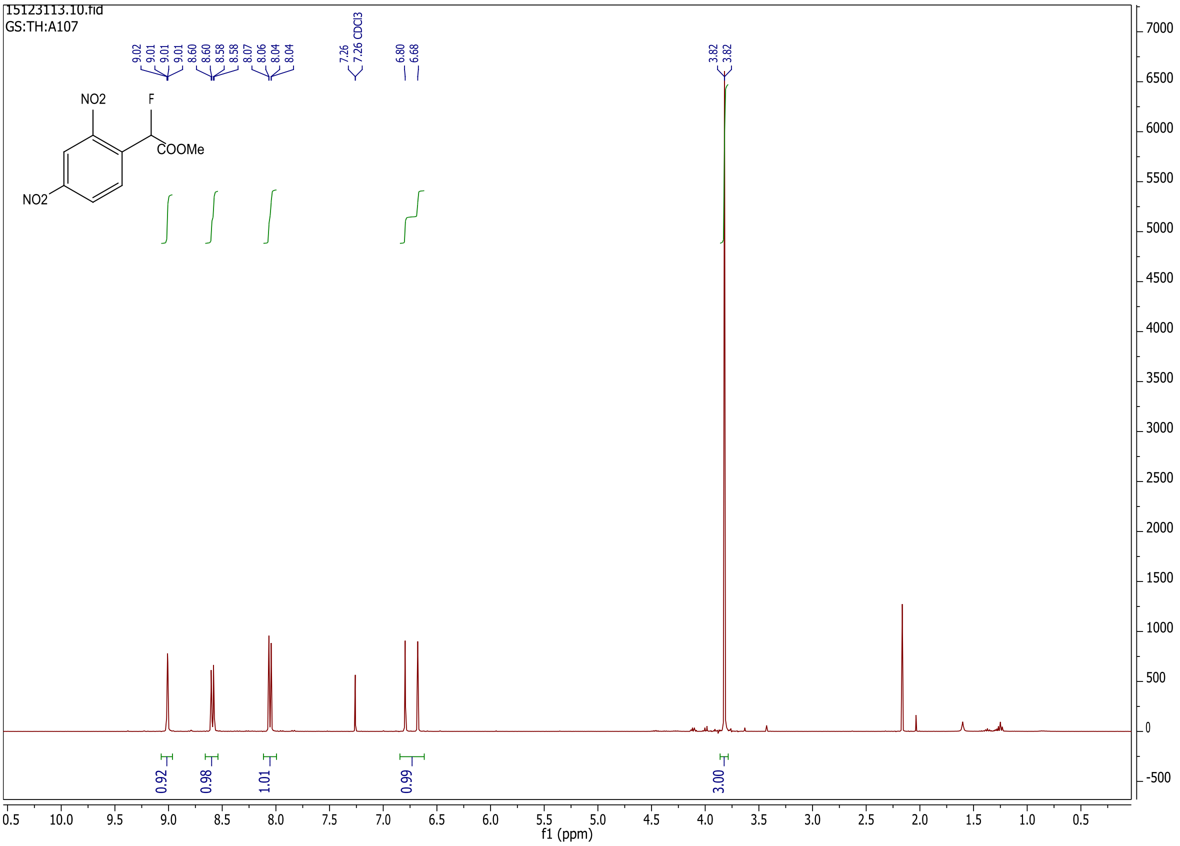

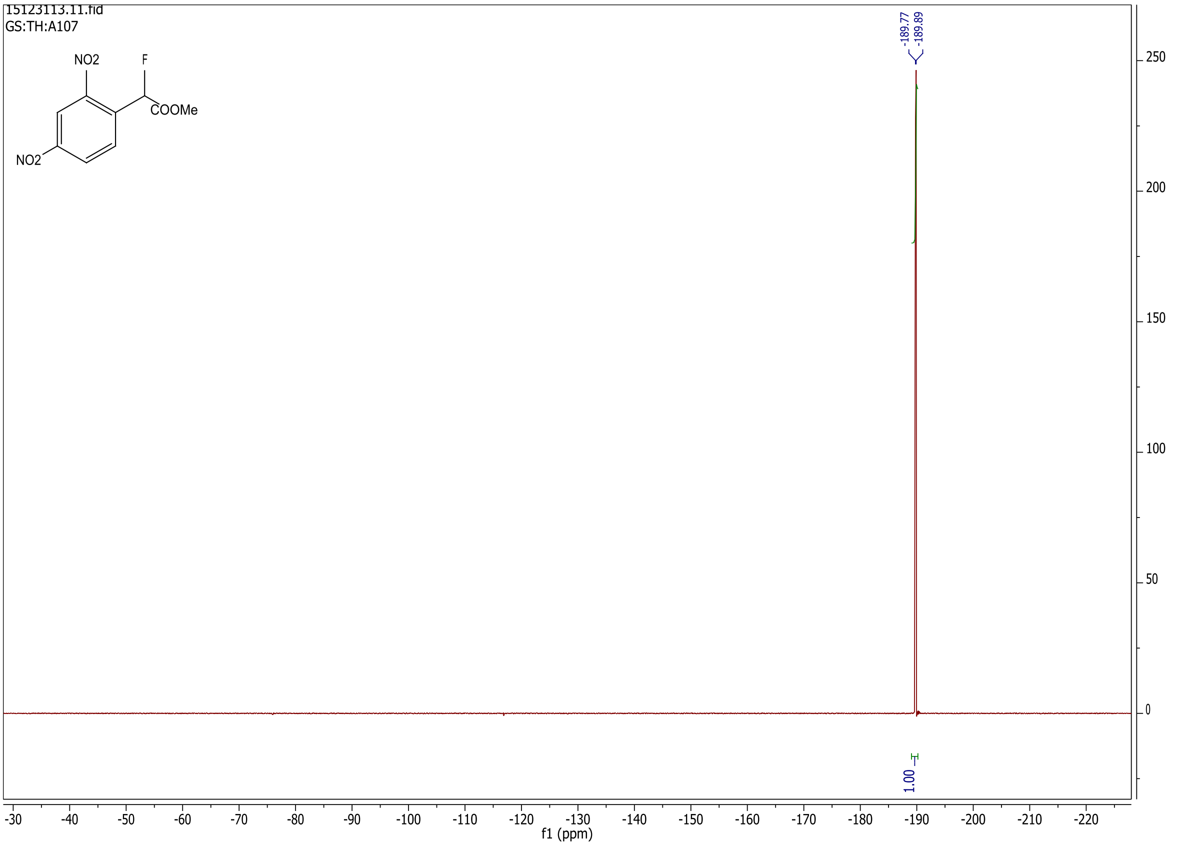

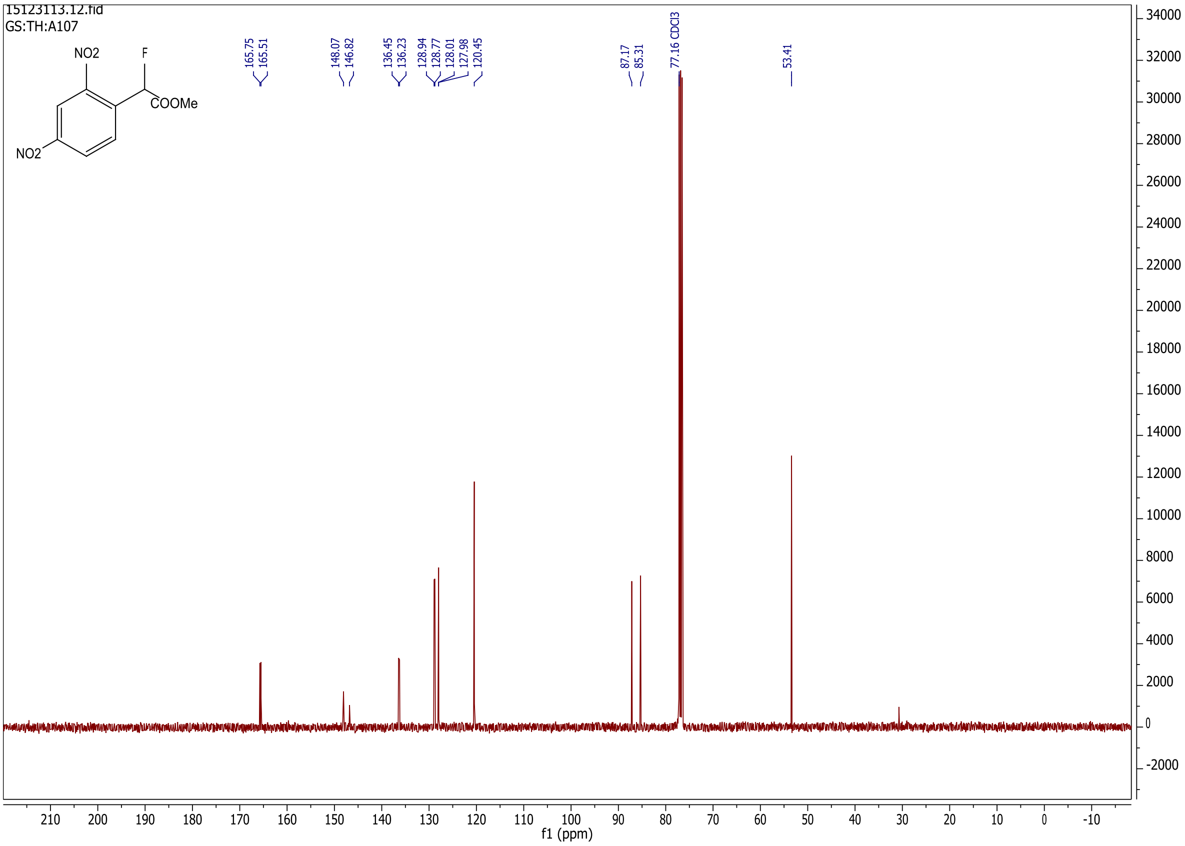
15123113.12.fid
GS:TH:A107
NO2
F
COOMe
NO2
165.75
165.51
148.07
146.82
136.45
136.23
128.94
128.77
128.01
127.98
120.45
87.17
85.31
77.16 CDCl3
53.41
f1 (ppm)

Methyl 2-fluoro-2-(2-nitro-5-methoxyphenyl)acetate (**6e**)

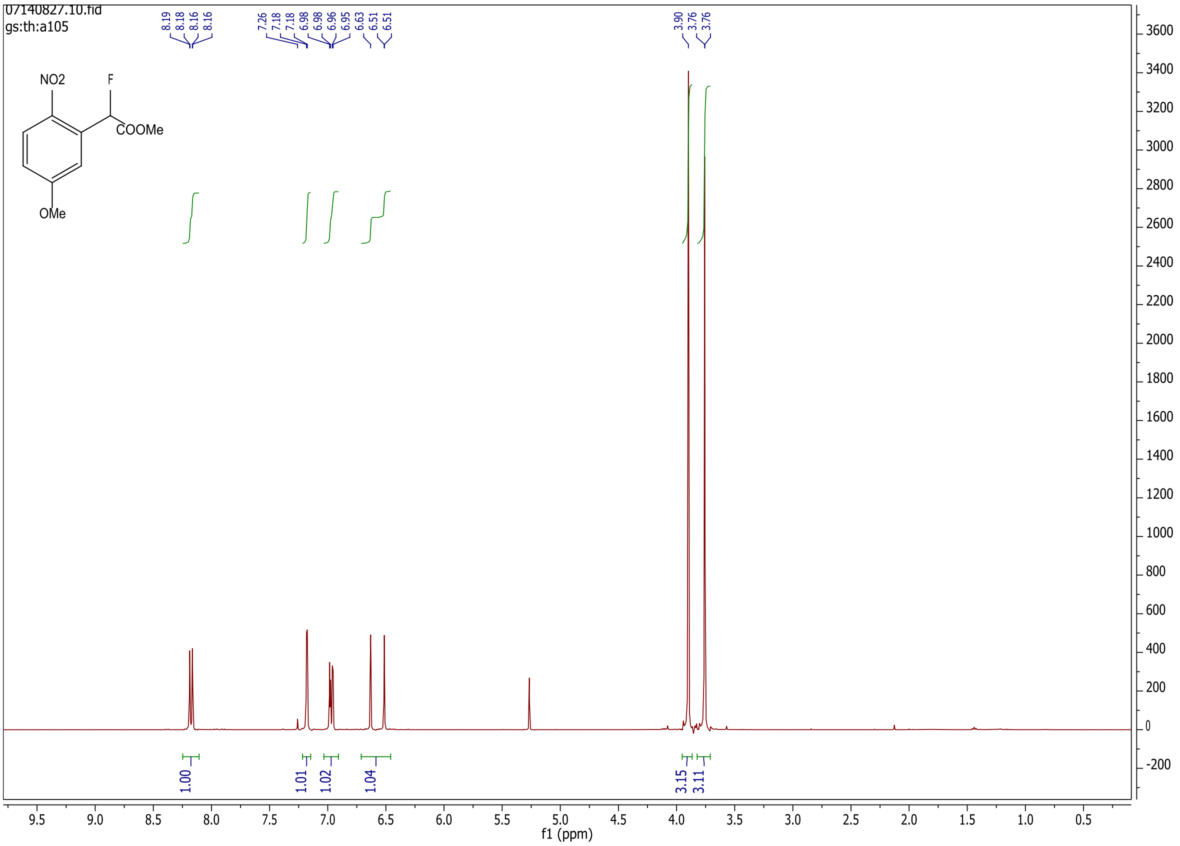

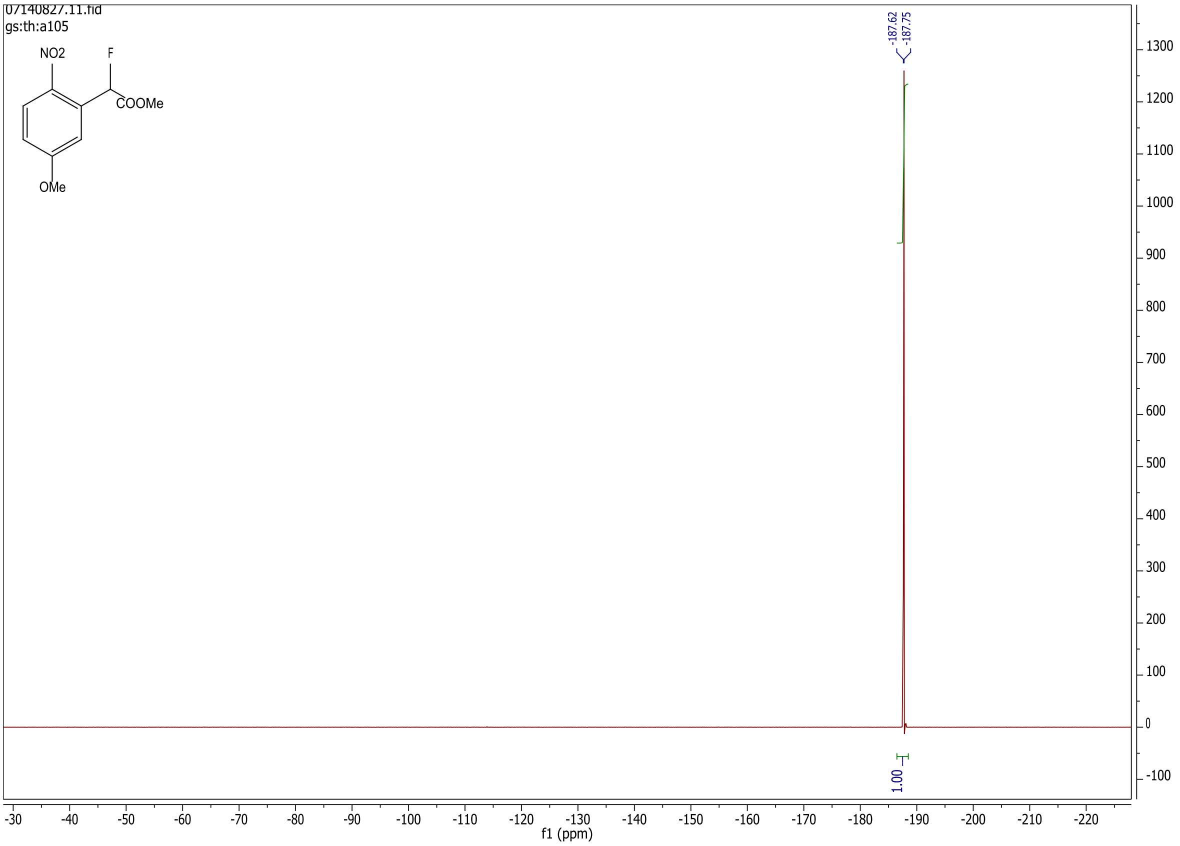

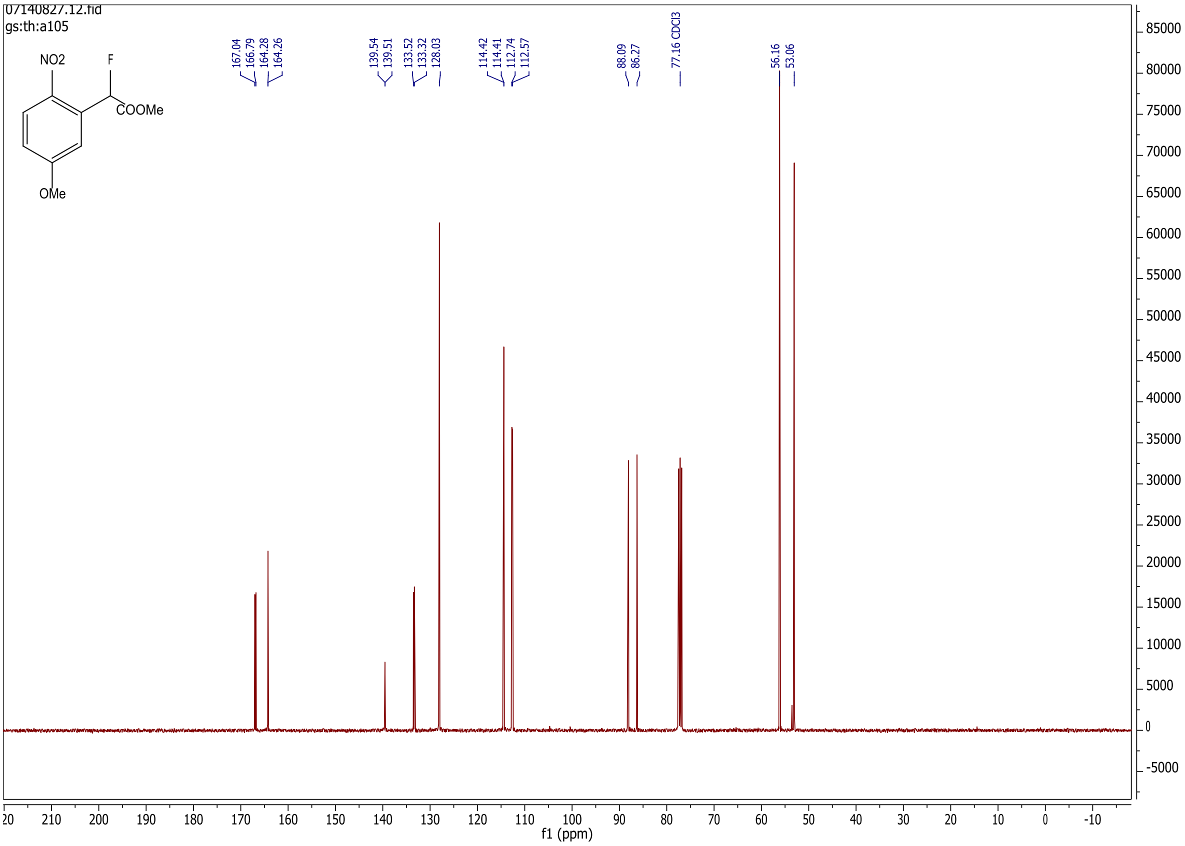
07140827.12.fid
gs:th:a105
NO2
F
COOMe
OMe
167.04
166.79
164.28
164.26
139.54
139.51
133.52
133.32
128.03
114.42
114.41
112.74
112.57
88.09
86.27
77.16 CDCl3
56.16
53.06
f1 (ppm)

3-Fluorooxindole (**8a**)

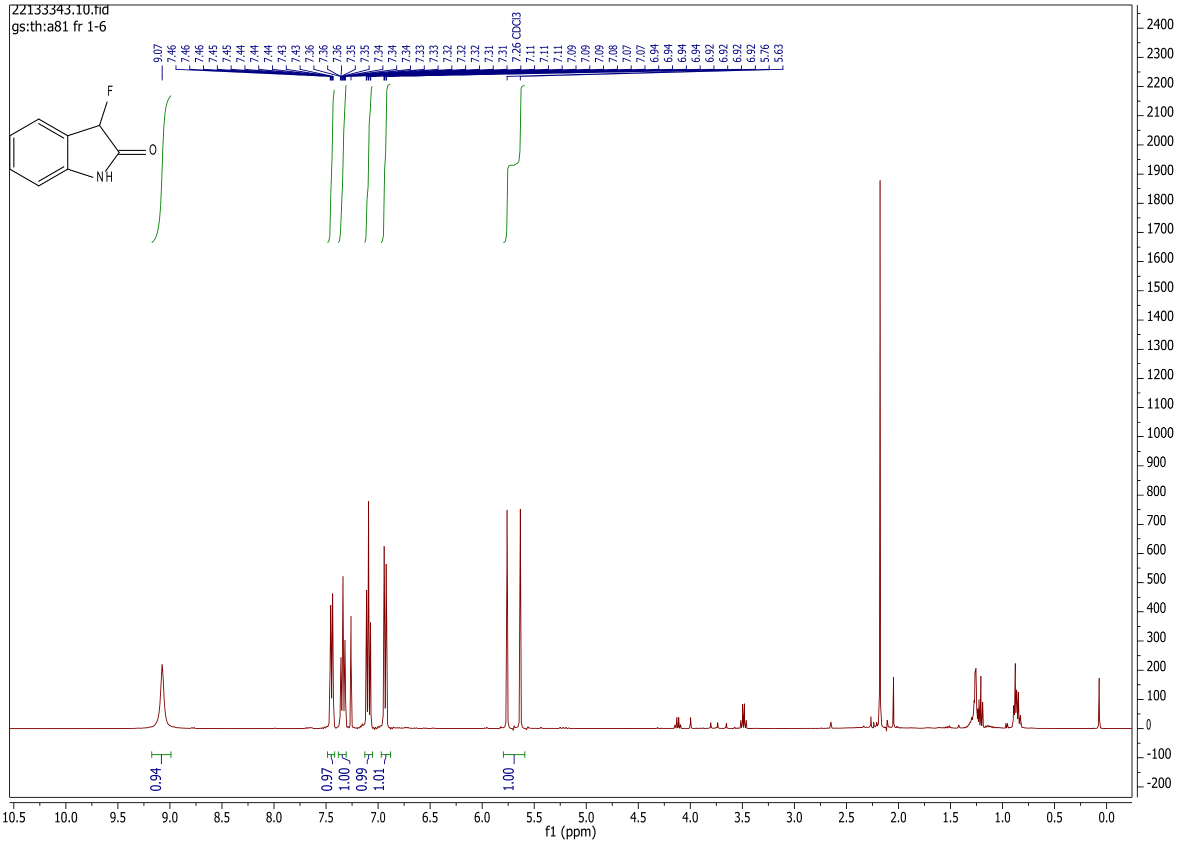

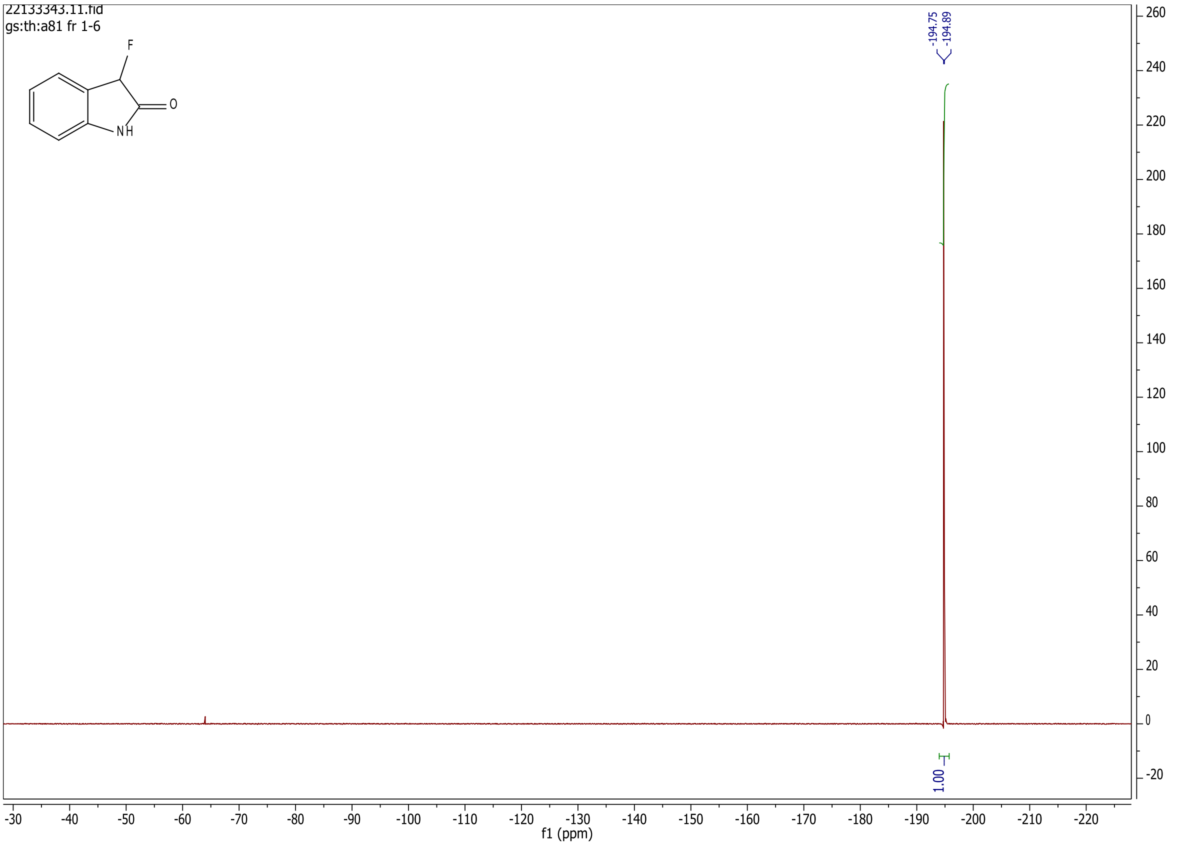

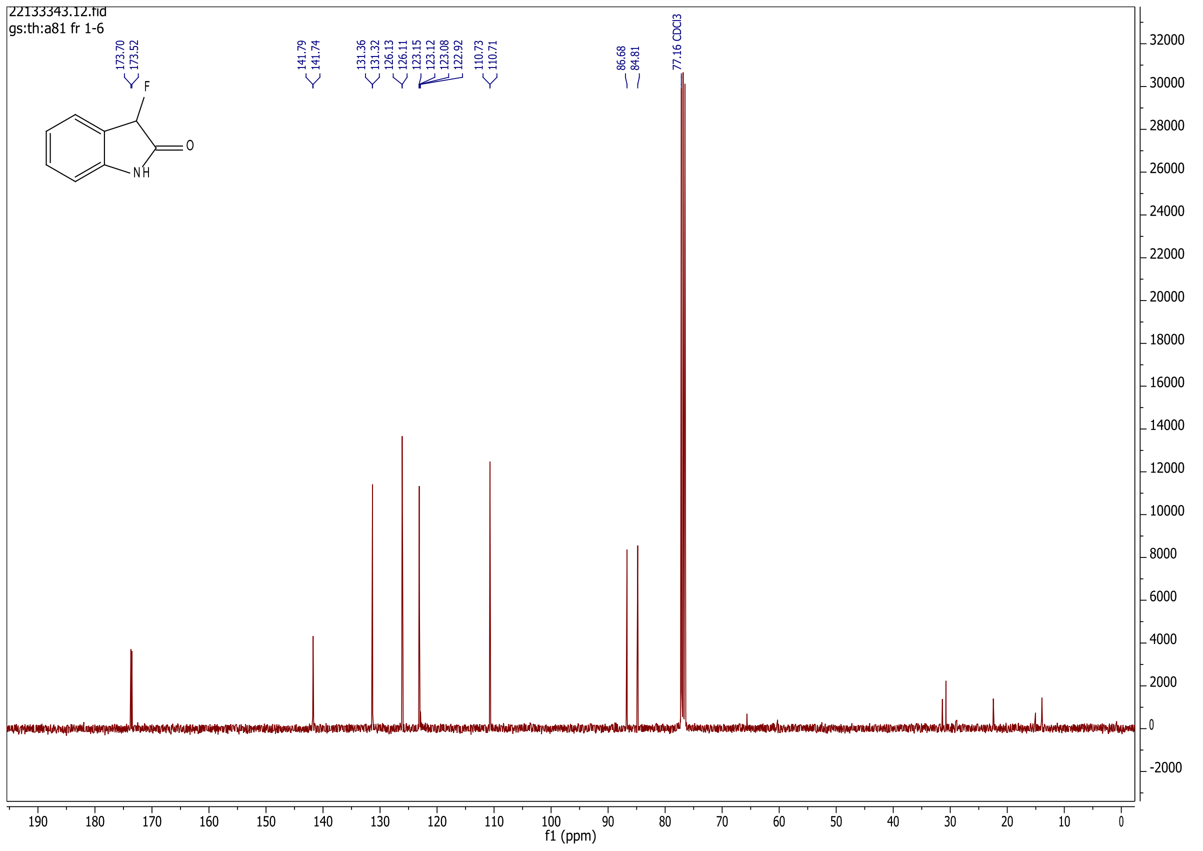

22133343.12.fid
gs:th:a81 fr 1-6
173.70
173.52
141.79
141.74
131.36
131.32
126.13
126.11
123.15
123.12
123.08
122.92
110.73
110.71
86.68
84.81
77.16 CDCl3
F
O
NH
f1 (ppm)

3-Fluoro-6-trifluoromethyloxindole (**8b**)

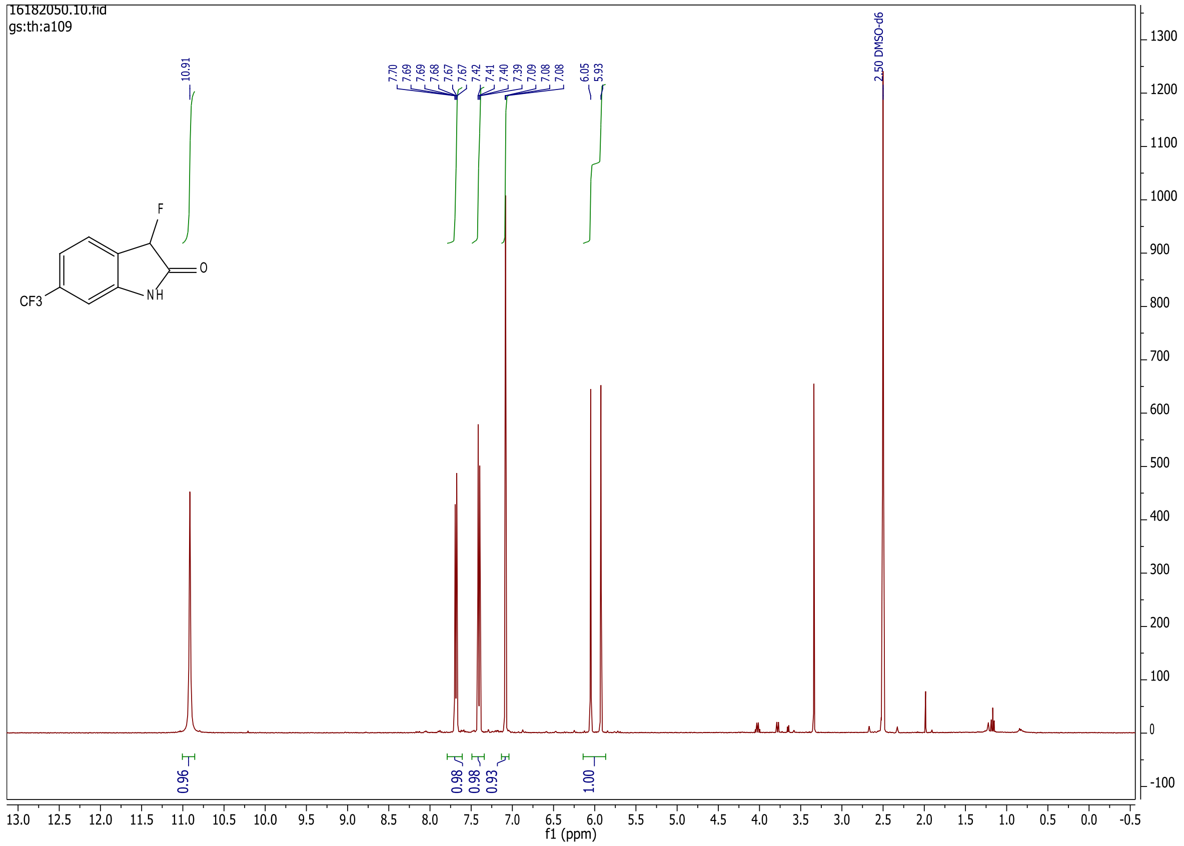

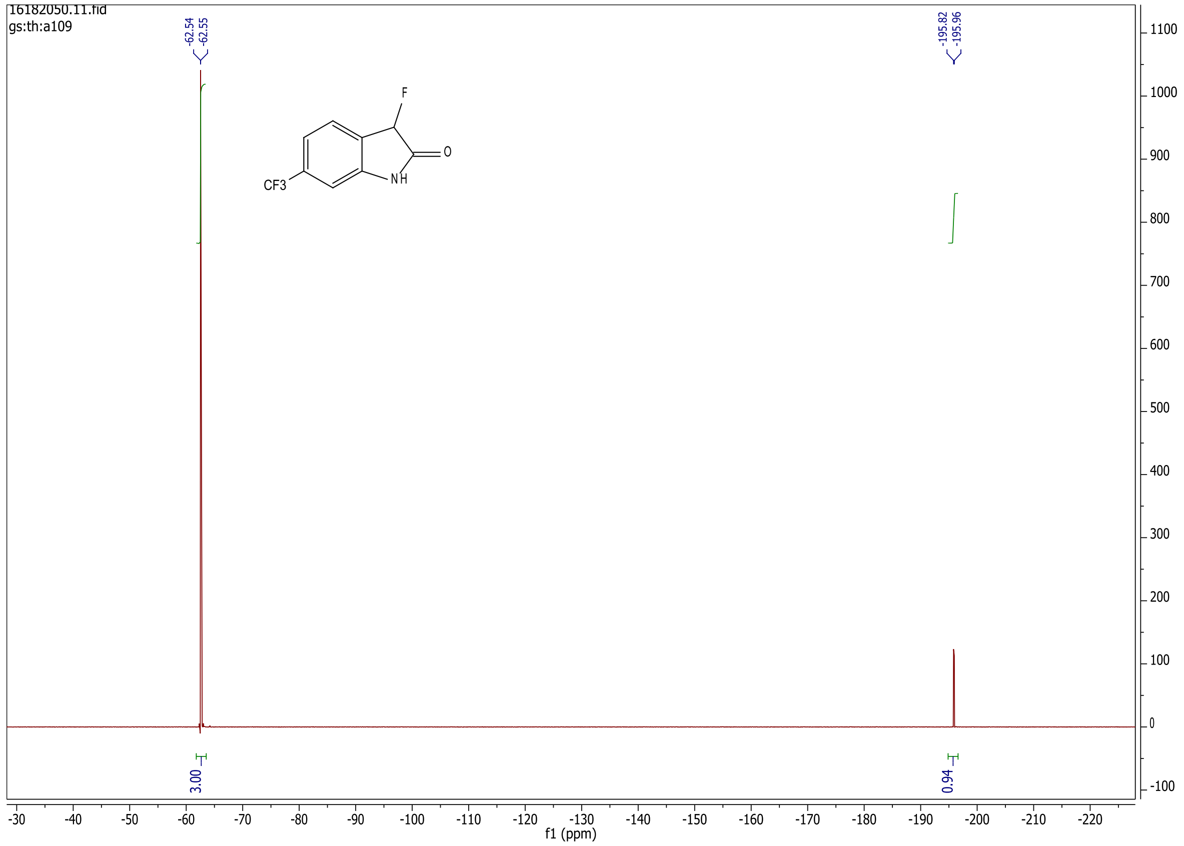

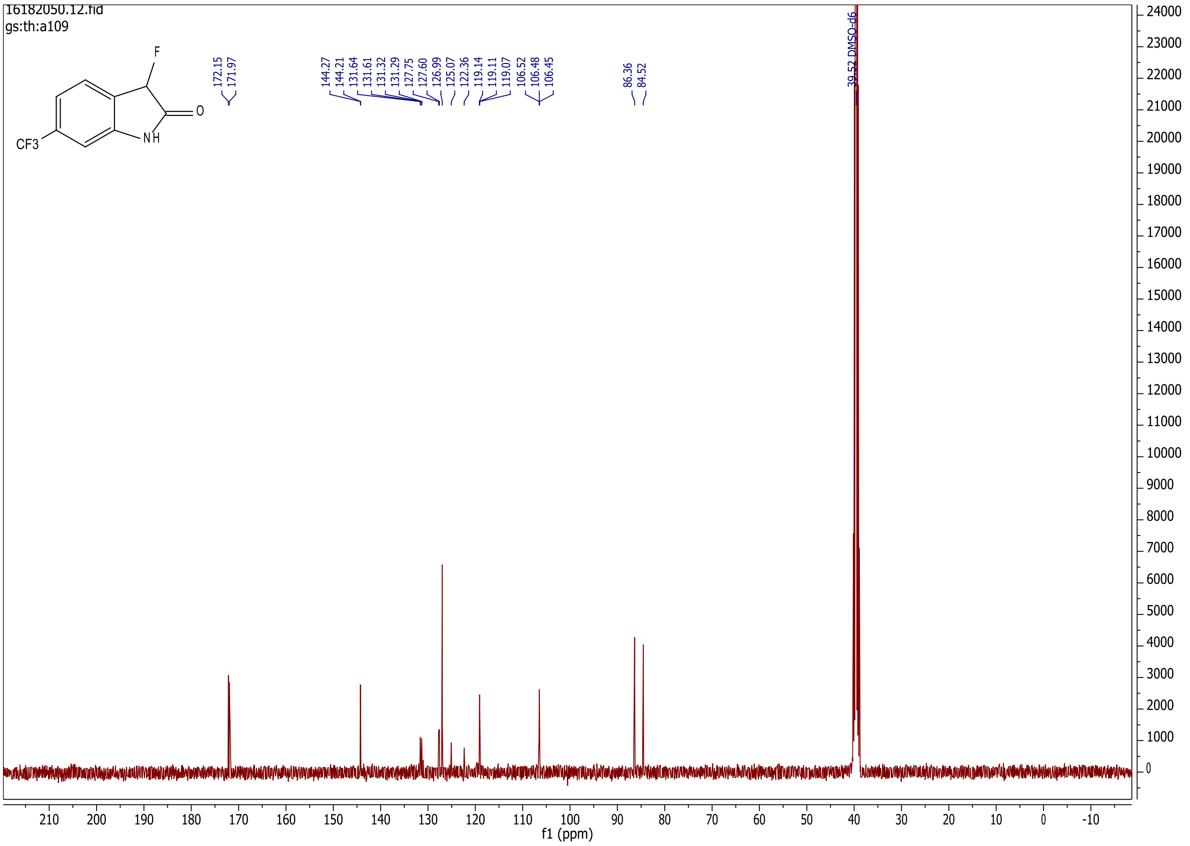
16182050.12.fid
gs:th:a109
F
O
NH
CF3
172.15
171.97
144.27
144.21
131.64
131.61
131.32
131.29
127.75
127.60
126.99
125.07
122.36
119.14
119.11
119.07
106.52
106.48
106.45
86.36
84.52
39.52 DMSO-d6
f1 (ppm)

3-Fluoro-6-bromooxindole (**8c**)

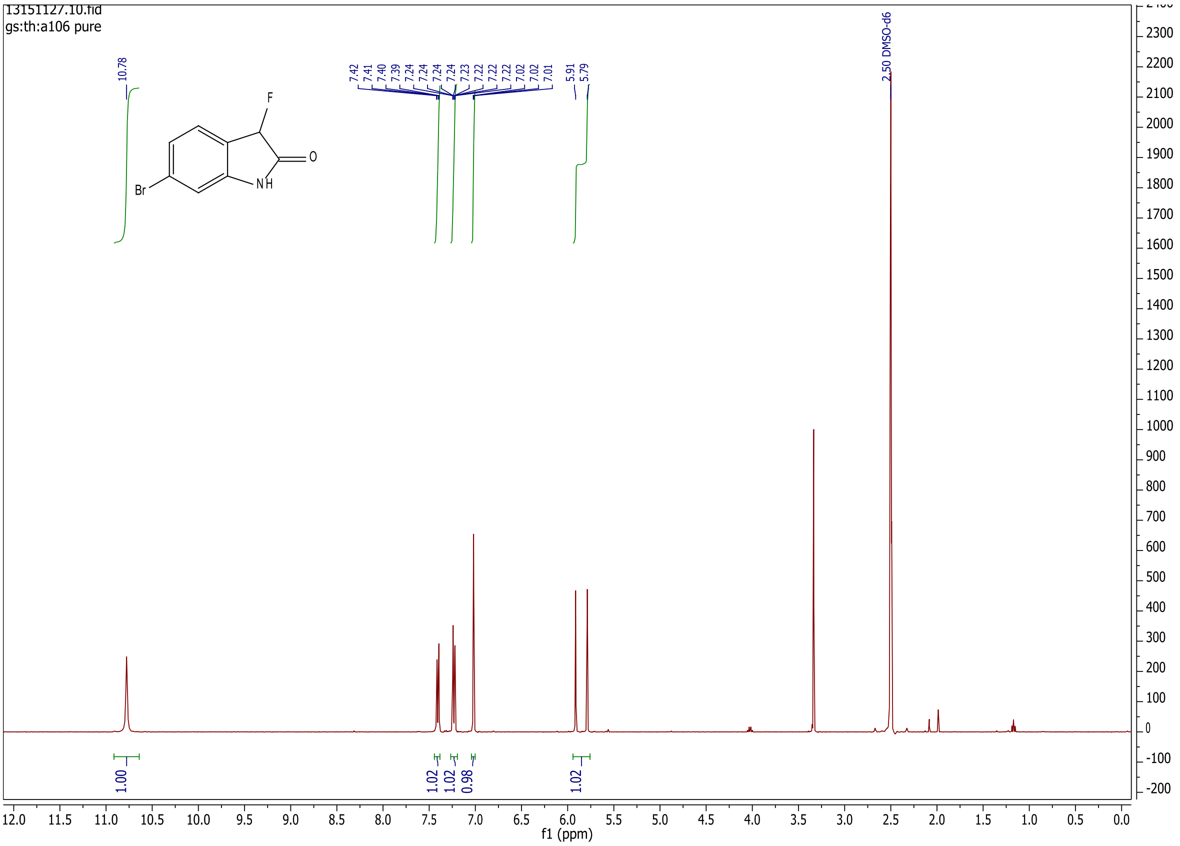

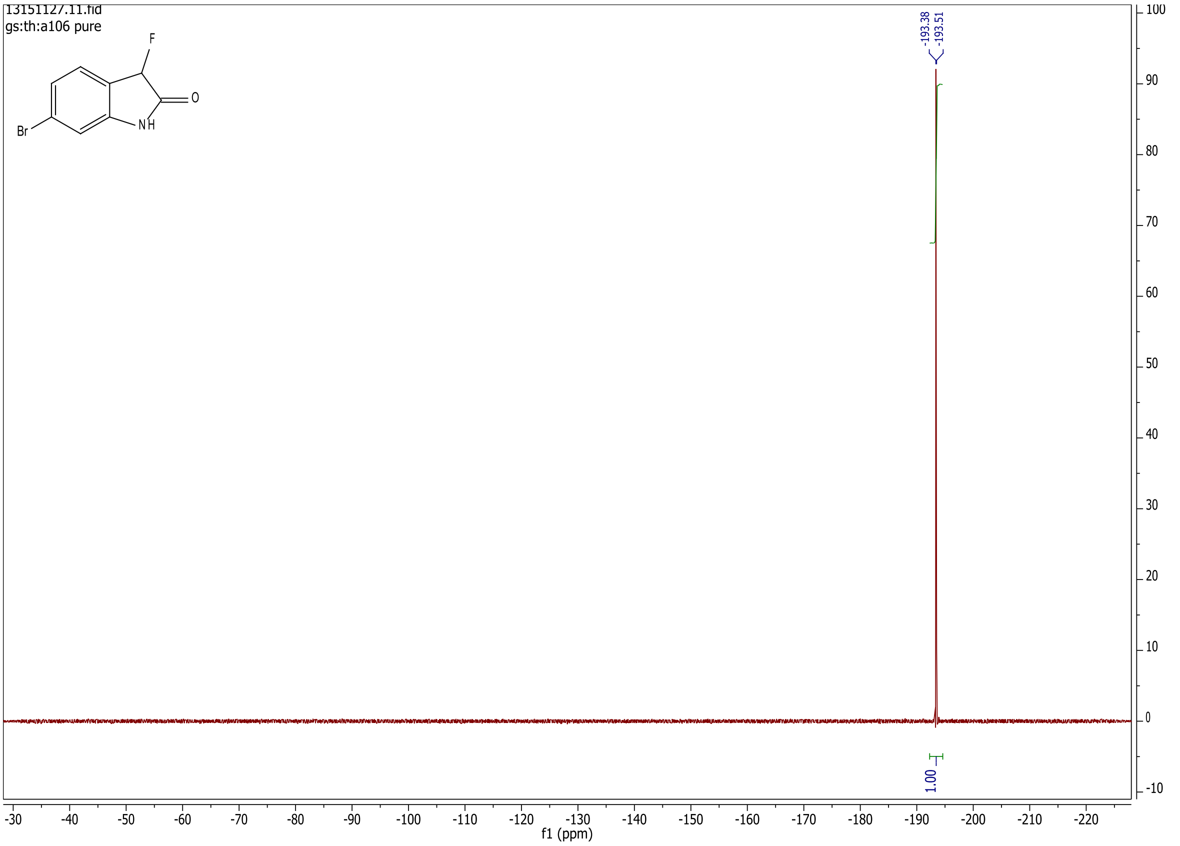

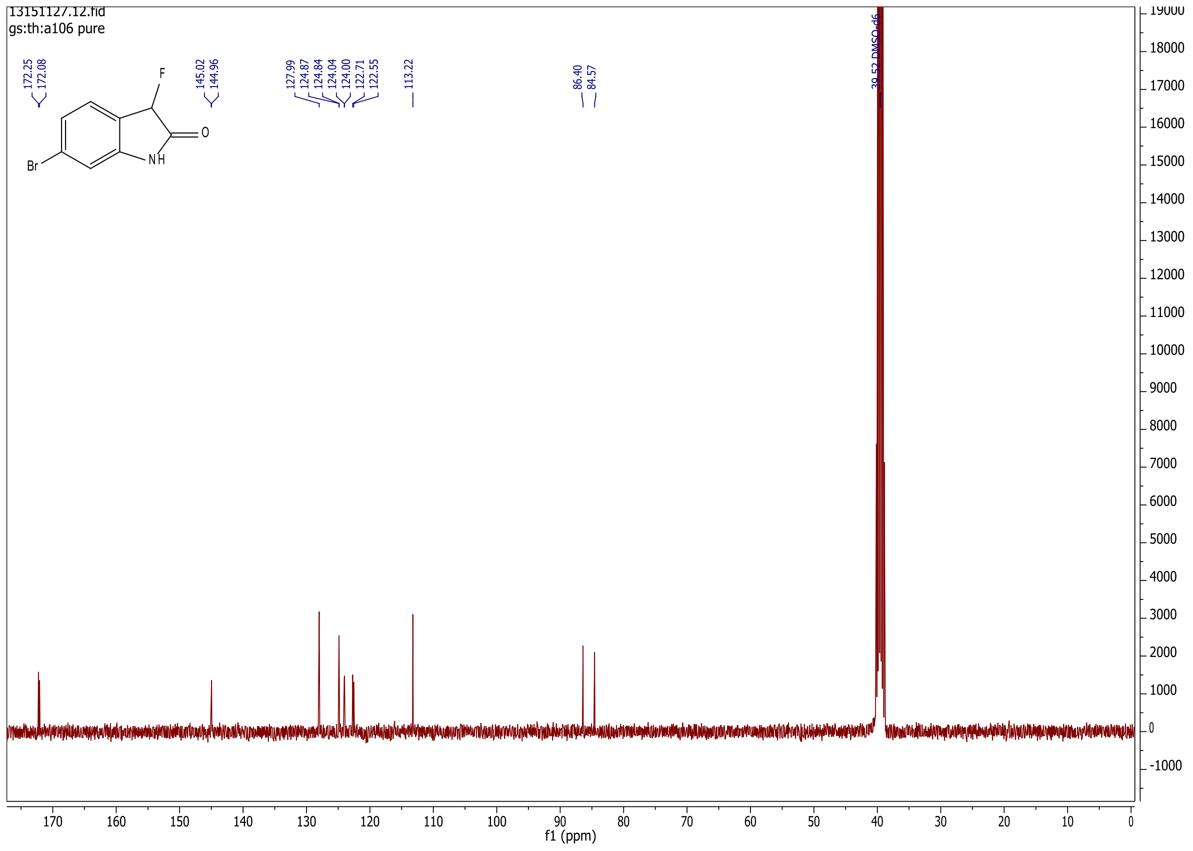

13151127.12.fid
gs:th:a106 pure
172.25
172.08
145.02
144.96
127.99
124.87
124.84
124.04
124.00
122.71
122.55
113.22
86.40
84.57
39.52 DMSO-d6
F
O
NH
Br
f1 (ppm)

3-Fluoro-5-methoxyoxindole (**8e**)

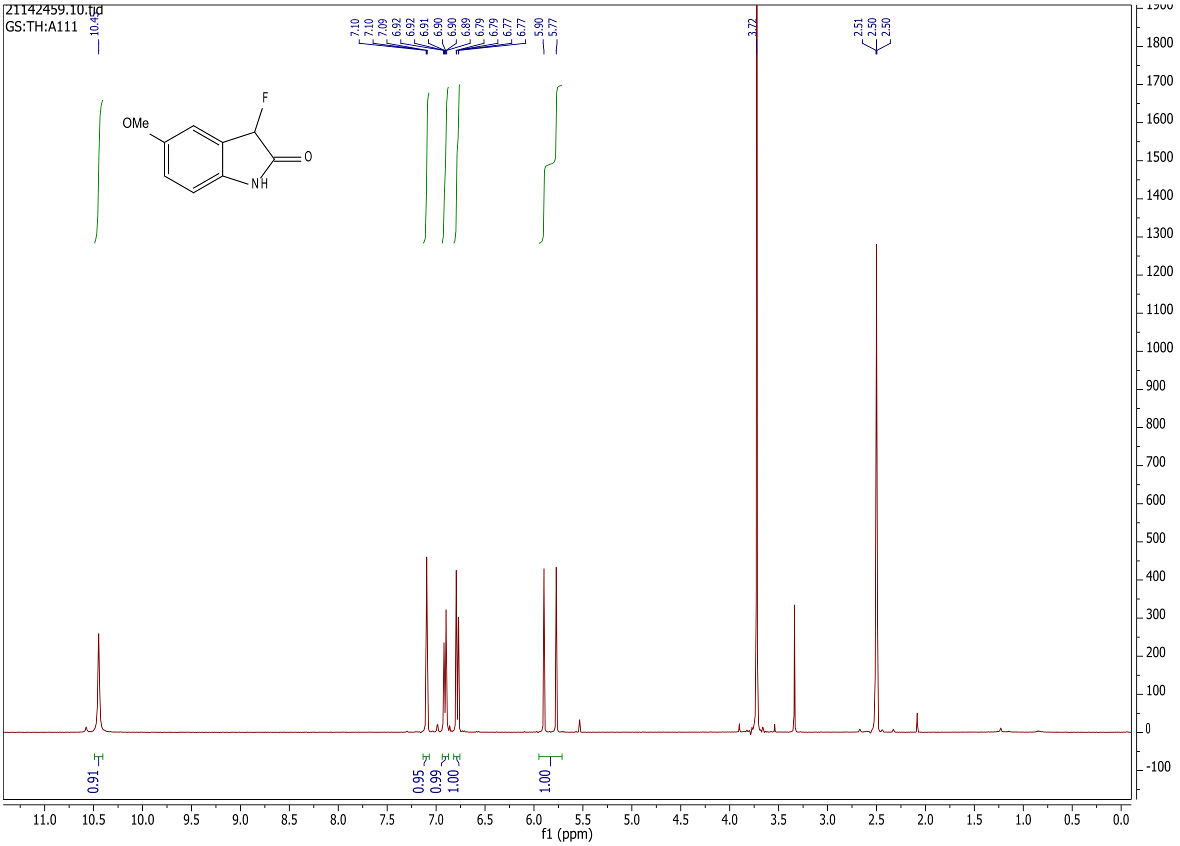

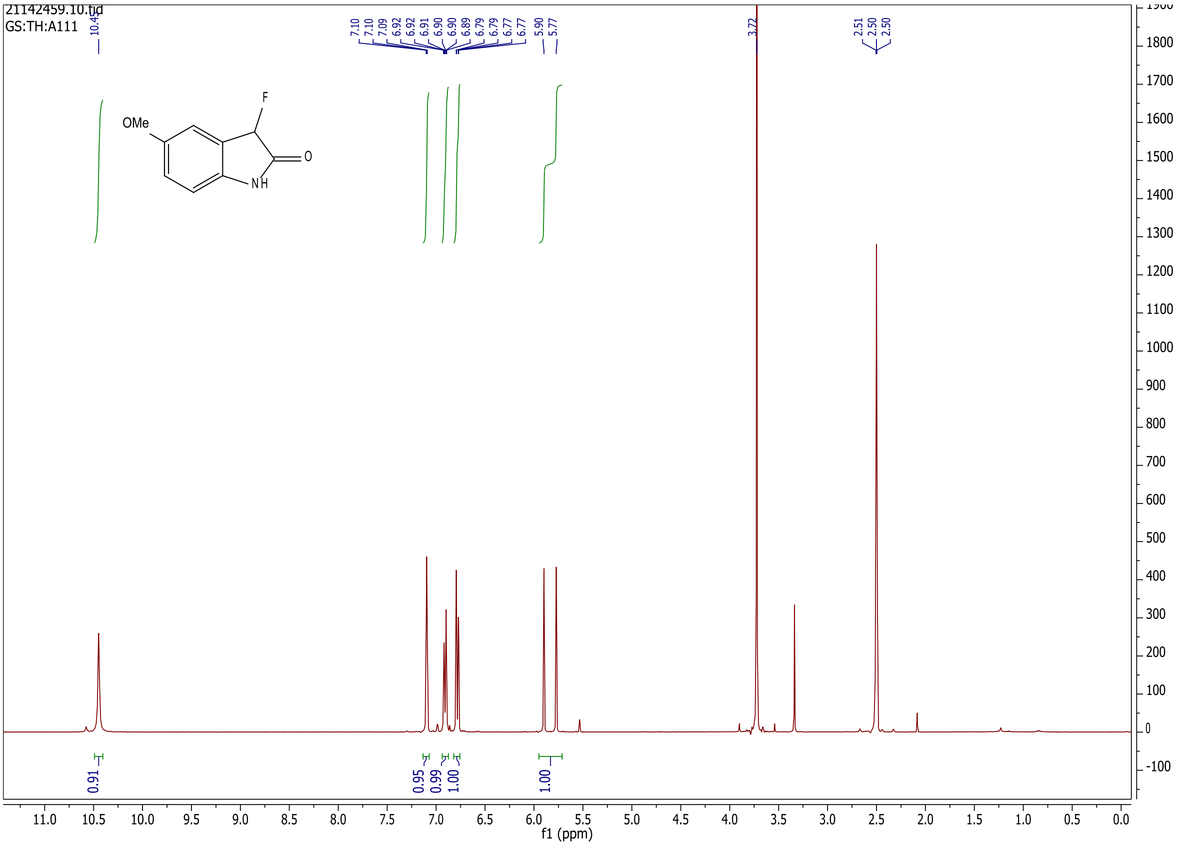

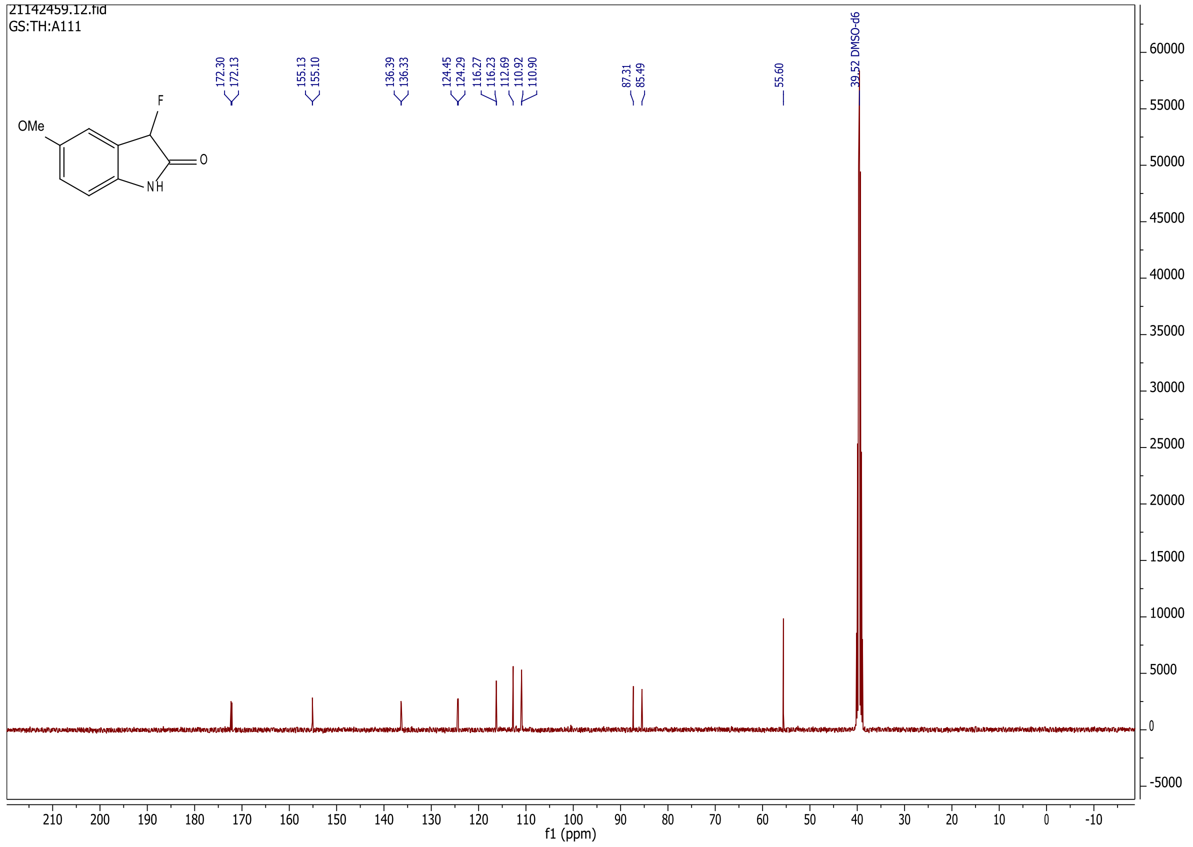
21142459.12.fid
GS:TH:A111
172.30
172.13
155.13
155.10
136.39
136.33
124.45
124.29
116.27
116.23
112.69
110.92
110.90
87.31
85.49
55.60
39.52 DMSO-d6
OMe
F
O
NH
f1 (ppm)

2,4-Dinitrobenzyl fluoride **5**

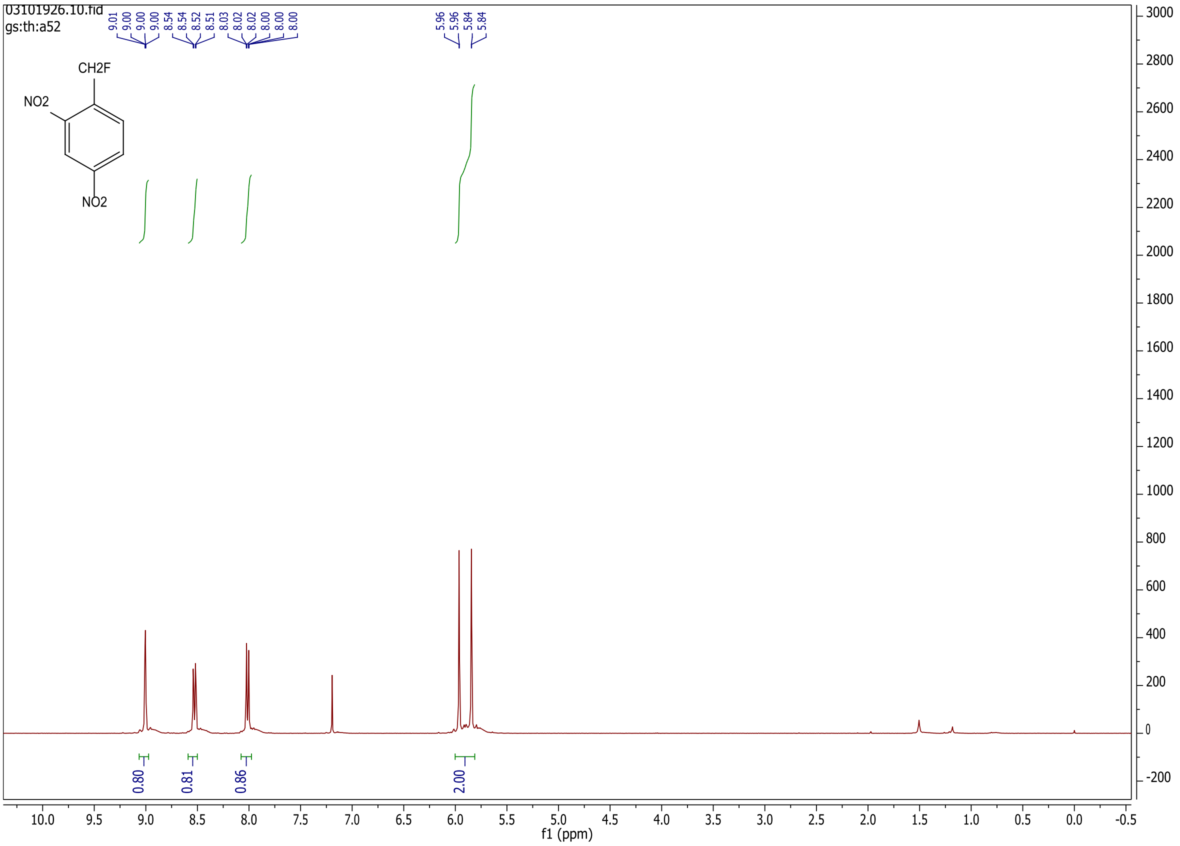

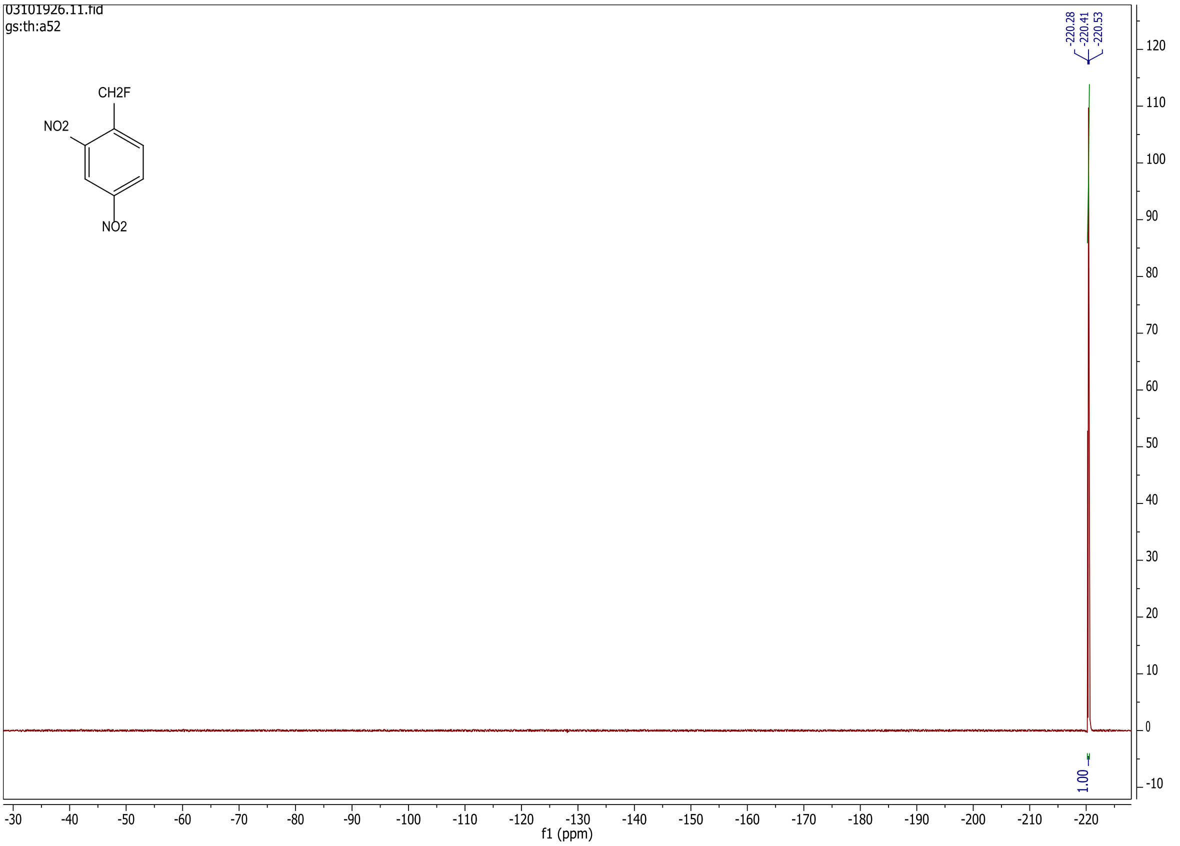

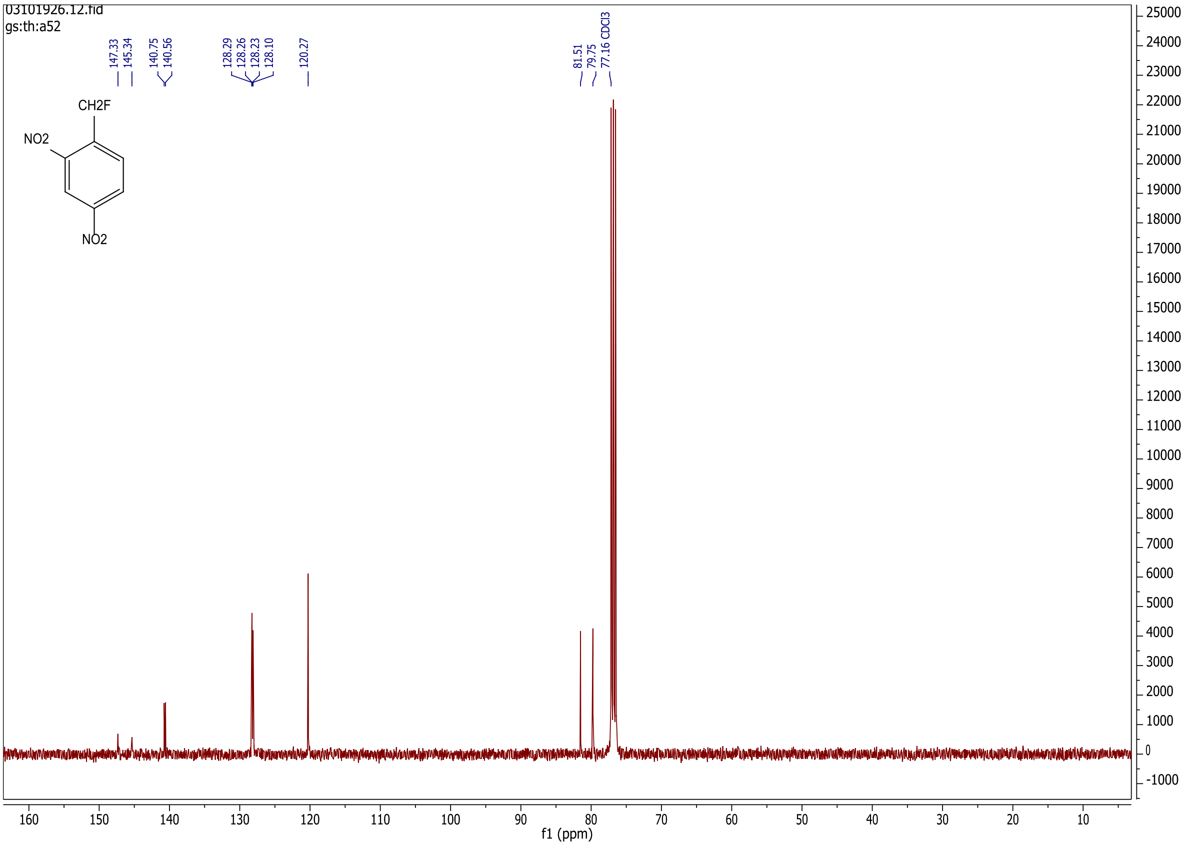

03101926.12.fid
gs:th:a52
147.33
145.34
140.75
140.56
128.29
128.26
128.23
128.10
120.27
81.51
79.75
77.16 CDCl3
CH2F
NO2
NO2
f1 (ppm)

2-Fluoromethyl-3-nitropyridine (**7**)

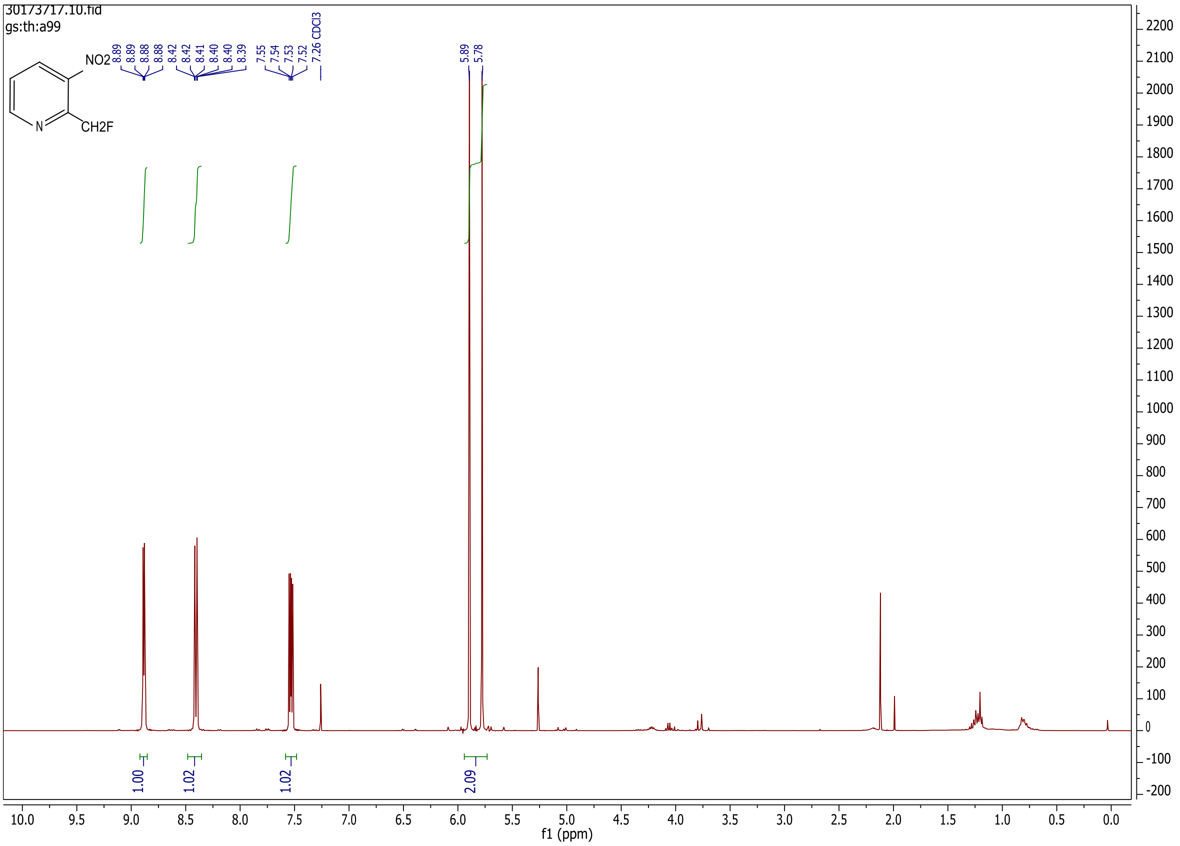

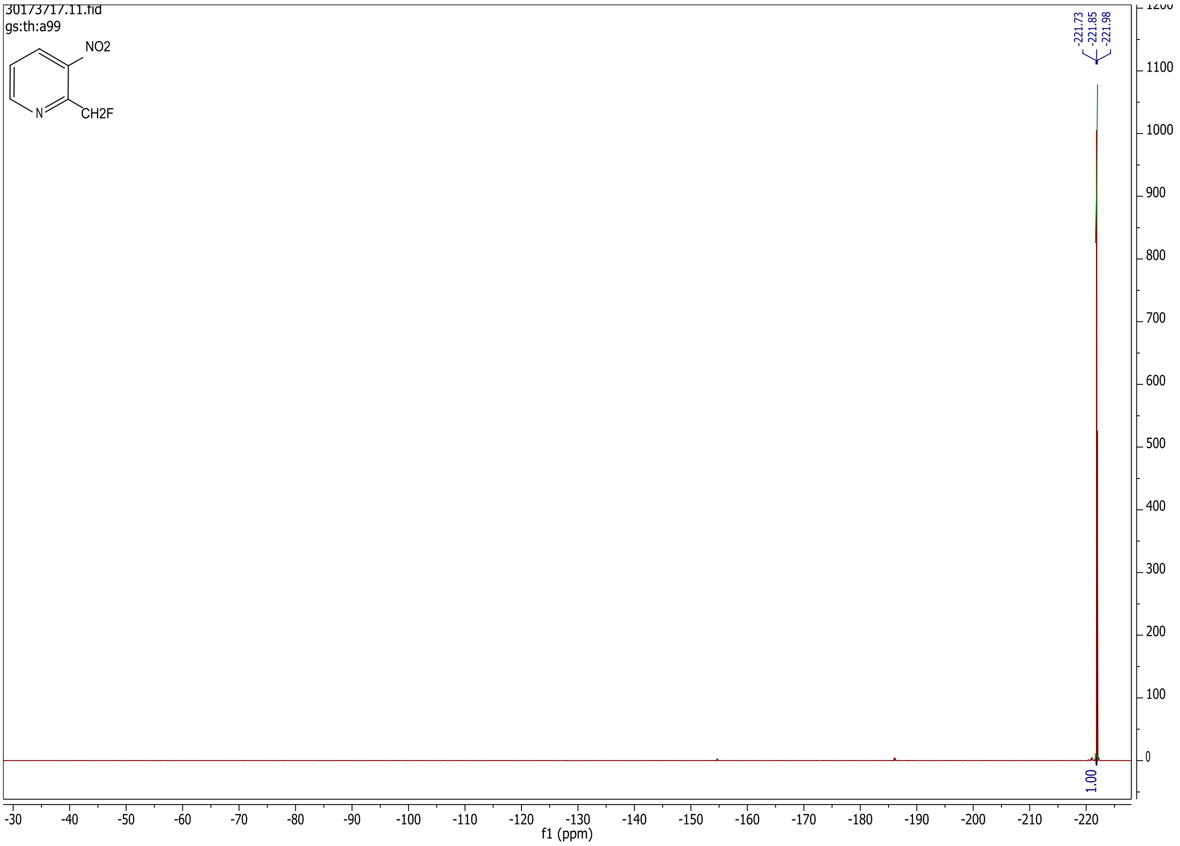

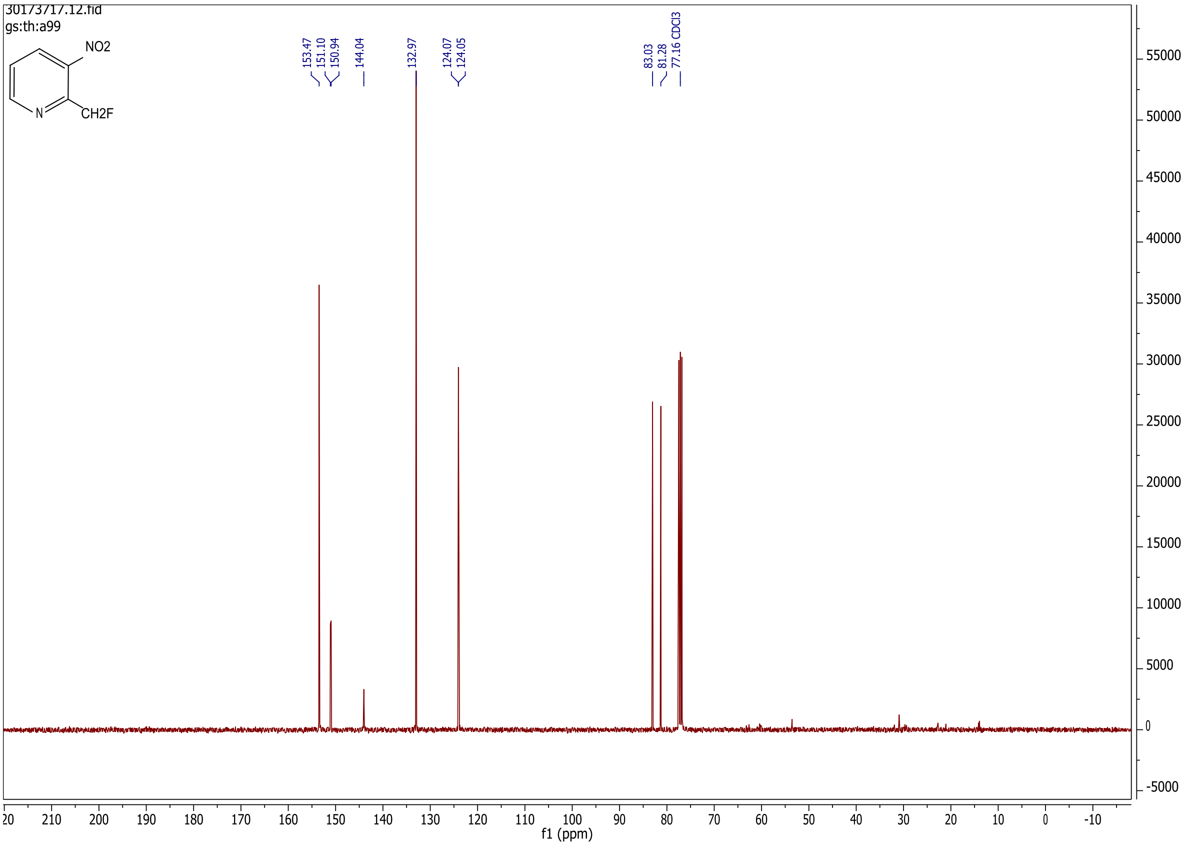

30173717.12.fid
gs:th:a99
NO2
N
CH2F
153.47
151.10
150.94
144.04
132.97
124.07
124.05
83.03
81.28
77.16 CDCl3
f1 (ppm)
